# Supplementary material for: Optimizing spatial equity of urban park cooling services: Integrating landscape metrics with K-means and PSO algorithms in Nanchang, China
Source: PLoS One. 2026 Mar 19;21(3):e0344026. doi: 10.1371/journal.pone.0344026 (PMC13001981; doi:10.1371/journal.pone.0344026)
Supplement: S1 File — (ZIP) [file pone.0344026.s001.zip › Supplementary material/Data sources and their processing tables.docx]

**Data sources and their processing tables**

| **Broad categories of data** | **Data subcategory** | **Data sources** | **Software and pre-processing** |
| --- | --- | --- | --- |
| Basic geographic data | Data on the administrative division of Nanchang | Geospatial data cloud | ArcGIS 10.6 |
|  | Remote sensing image data | Geospatial data cloud | Surface temperature inversion in ENVI 5.6 software |
|  | Land classification data | Database of national land cover maps at 1m resolution | ArcGIS 10.6 (mask extraction, raster to surface, feature classification extraction), Fragstats 4.2 software to extract six landscape pattern indices in UPGS |
|  | Road network data | OpenStreetMap Open Shared Database (www.openstreetmap.org) | ArcGIS 10.6 (merge, intersect) |
| Community and demographic data | Name, latitude and longitude, number of households, house price | Using Python to Crawl Community POI Data from Anjuke (https://wuhan.anjuke.com/) in Nanchang Study Area | ArcGIS 10.6 (Kriging interpolation, partitioning statistics, creation of fishing nets, attribute table joins and associations, labelling, extraction of coordinate points with corresponding latitude and longitude, manual method of classification, intersections) |
|  | Demographic data | The product of the number of households in the community and the population of the administrative unit (SCMS) is used to calculate the population size of the community | ArcGIS 10.6 (Kriging interpolation, partitioning statistics, creation of fishing nets, attribute table joins and associations, labelling) |
| UPGS data | Name, latitude, longitude, area | Baidu's online map | Python crawls the Point of Interest (POI) directory of Nanchang UPGS, and then accesses the Baidu map API to crawl the Area of Interest (AOI) data of UPGS and import it into the map to generate vector layers. |
