# Supplementary material for: Optimizing spatial equity of urban park cooling services: Integrating landscape metrics with K-means and PSO algorithms in Nanchang, China
Source: PLoS One. 2026 Mar 19;21(3):e0344026. doi: 10.1371/journal.pone.0344026 (PMC13001981; doi:10.1371/journal.pone.0344026)
Supplement: S1 File — (ZIP) [file pone.0344026.s001.zip › Supplementary material/Exploration of Inequity.docx]

In the first paragraph of Section 3.4 of the paper, we added descriptions of the Gini coefficient and Moran’s I. However, due to space limitations, we have placed the supporting materials in the supplementary section, as detailed below：We first used the Thiessen polygon tool in ArcGIS to convert park green spaces into point data, and then applied the Thiessen polygon tool again to perform spatial division, obtaining the Thiessen polygon pattern of park green spaces in the study area (Figure 1). Next, we used the field calculator to compute the coefficient of variation for the spatial distribution of park green spaces, which measures the degree of spatial variation of park points within the Thiessen polygons.The calculated coefficient of variation (CV) was 167.96%, which is greater than 64% (Table 1). This indicates that the park green spaces in the main urban area of Nanchang exhibit a clustered spatial distribution, confirming an imbalanced distribution of park green spaces across the area.

Table 1 Coefficient of variation

| **Formula for coefficient of variation** | **Standard deviation (S)** | **Mean (V)** | **Coefficient of variation (CV)** |
| --- | --- | --- | --- |
| CV=S/V | 20584392.33m^2^ | 12255473.92m^2^ | 167.96% |


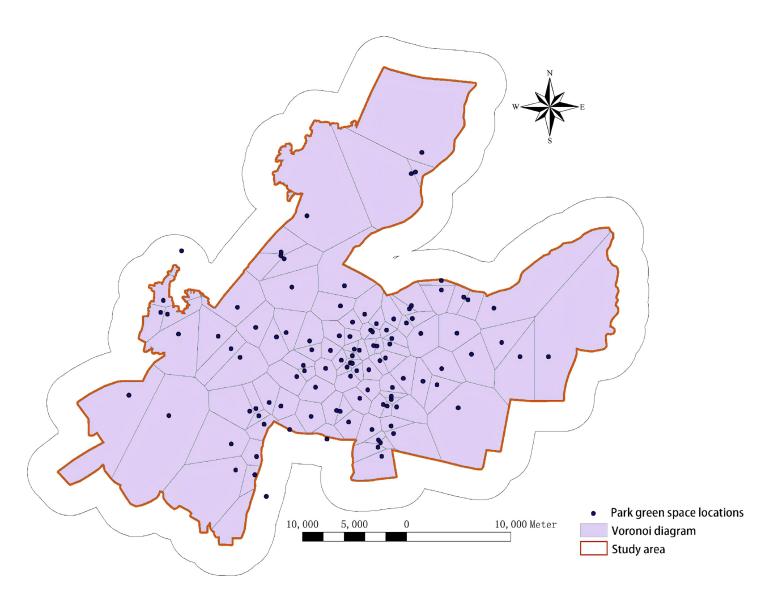


Fig 1 Tyson polygon of green space in parks in the main urban area of Nanchang City

Secondly, using the global spatial autocorrelation tool in ArcGIS, we calculated the global Moran’s I for 67 neighborhoods in the main urban area of Nanchang to analyze the spatial correlation characteristics of park green spaces at the neighborhood level. The results show that Moran’s I is 0.086847, which is greater than 0, indicating a positive spatial correlation in the distribution of park green spaces within the study area. Additionally, the z-score is 1.704590, and the p-value is 0.088271, meaning the probability of this clustered pattern occurring randomly is less than 10%. This further confirms that the distribution of park green spaces exhibits a clustering trend.

Table 2 Global Moran I Summary.

| **Moran's I index** | **Expected index** | **Variance** | **Z-score** | **P-value** |
| --- | --- | --- | --- | --- |
| 0.086847 | -0.015152 | 0.003581 | 1.704590 | 0.088271 |


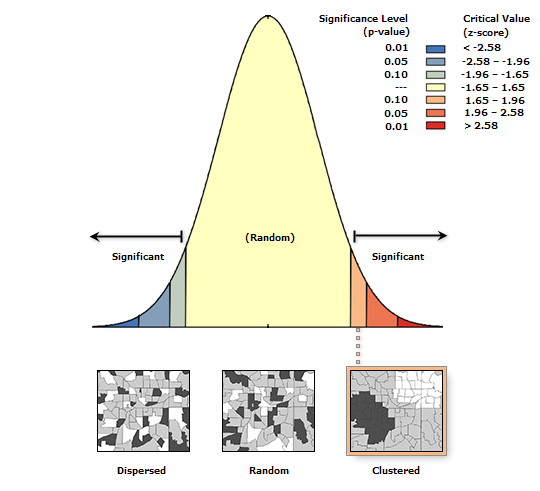


Fig2 Moran's index of green space in parks in the main urban area of Nanchang City

Finally, we introduced the Lorenz curve to visualize the equity of the distribution of green space resources relative to the population in each neighborhood. A flatter curve suggests a more equal distribution of green space resources, while a steeper curve indicates greater inequality (Figure 3). As shown in the figure, the Lorenz curve is relatively steep, with Area A being significantly larger than Area B, indicating a poor supply-demand balance between park green spaces and population at the neighborhood level.Furthermore, based on the Lorenz curve, we applied the Gini coefficient formula $G=1-\sum_{K=1}^{n} (p_{k}-p_{k-1})(R_{K}-R_{K-1})$ to calculate the area ratio of different regions represented by the curve.  The Gini coefficient was used to assess the fairness of park green space distribution among the neighborhood populations. The calculated Gini coefficient for green space resource distribution across neighborhoods in Nanchang’s central urban area is 0.58, which is greater than 0.5. This suggests a considerable disparity in the allocation of park green space resources among the neighborhoods in Nanchang’s central urban area.


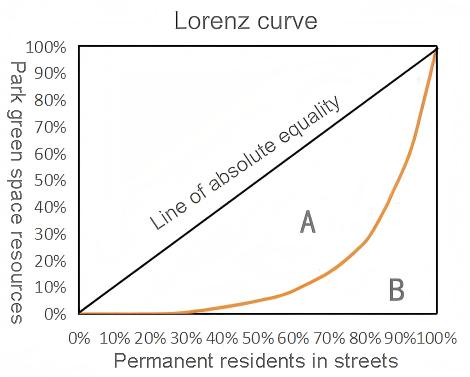


Fig 3 Lorentz curve

**Part 3):** *The optimization results (18 new parks) lack quantitative validation of improvement — e.g., before/after comparison of accessibility or spatial equity indices: We sincerely thank the reviewer for this crucial suggestion. In direct response, we have now conducted a comprehensive quantitative validation of the optimization results through a before-and-after comparison, which is detailed in the revised manuscript as follows:A new quantitative validation has been added to Section 3.4. We have integrated a paragraph describing the simulation process, where the 18 proposed UPGS were assigned the optimal cooling service range (PCA) of 2853 meters derived from our algorithms. The results of this simulation demonstrate a substantial improvement: the population coverage within a 15-minute walking distance increased from the original 71.2% to 92.5%, an absolute gain of 21.3%. This provides the direct quantitative comparison requested. The key finding of this validation has been incorporated into the Conclusion. To highlight the significance of this result, we have now stated the coverage improvement in the conclusion, reinforcing the practical efficacy of our optimization framework. A new figure (Fig. 10b) has been provided to visualize this improvement. The original Fig. 10b has been replaced with a new version that visually illustrates the enhanced coverage after optimization, offering an intuitive complement to the quantitative data.

(4) Discussion and Conclusion are largely descriptive

**Response:** Discussion and Conclusion are largely descriptive... We thank the reviewer for these insightful comments. In response, we have thoroughly revised the manuscript to enhance its critical depth. Regarding the comparison with other optimization methods: We have added a critical discussion in Section 4.3 that explicitly compares our PSO algorithm with traditional methods like GA and ACO, justifying our methodological choice. Regarding policy implications: We have acknowledged the value of this suggestion. However, to maintain a concise focus on the core scientific contributions and within the constraints of the article's length, we have opted to integrate the key practical insights into the scientific discussion rather than expand them into a dedicated policy section. We believe the current version effectively bridges our findings with planning practice. Regarding the deeper analysis of limitations: We have significantly strengthened Section 4.4 ("Research Limitations and Future Directions") by providing a more profound analysis of the influence of seasonality (single-date imagery), population heterogeneity, and land-use constraints on our results and their interpretations.
