# Supplementary material for: Optimizing spatial equity of urban park cooling services: Integrating landscape metrics with K-means and PSO algorithms in Nanchang, China
Source: PLoS One. 2026 Mar 19;21(3):e0344026. doi: 10.1371/journal.pone.0344026 (PMC13001981; doi:10.1371/journal.pone.0344026)
Supplement: S1 File — (ZIP) [file pone.0344026.s001.zip › Supplementary material/k-w test and correlation analysis/Correlation analysis of landscape pattern indices.doc]

One-sample Kolmogorov-Smirnov test	
	Average Surface Temperature		Cooling Range		Cooling range		Gradient of cooling		Impervious surface
 PD																		
Number of cases	42	42	42	42	42																		
Normal parameters	Mean value	39.45450600	423.571	1.224692142857143	.004169683585892	901.250888																		
	Standard deviation	2.535403553	332.3664	.929808126232231	.003854540328412	665.4919609																		
most extreme difference	Absolute	.076	.123	.119	.169	.162																		
	¥¿	.076	.123	.119	.169	.162																		
	负	-.069	-.118	-.098	-.146	-.101																		
inspection statistics	.076	.123	.119	.169	.162																		
Asymptotic significance (two-tailed)	.200c,d	.110c	.149c	.004c	.007c																		

One-sample Kolmogorov-Smirnov test	
	绿¦aPD	¤ôÊ^PD	¤£³z¤ô­±LPI	绿¦aLPI	¤ôÊ^LPI													
个®×数	42	42	42	42	42													
¥¿态参数a,b	¥­§¡­È	578.900281	367.848045	5.288400	56.391929	22.018762													
	标­ã®t	564.9157815	416.6902729	10.0370544	31.3454044	27.8812483													
³ÌÌåºÝ®t­È	绝对	.165	.197	.309	.198	.245													
	¥¿	.165	.197	.309	.155	.245													
	负	-.157	-.189	-.300	-.198	-.215													
检验统计	.165	.197	.309	.198	.245													
渐ªñ显µÛ©Ê¡]双§À¡^	.006c	.000c	.000c	.000c	.000c													

One-sample Kolmogorov-Smirnov test	
	¤£³z¤ô­±LSI	绿¦aLSI	¤ôÊ^LSI	¤£³z¤ô­±DIVISION	绿¦aDIVISION								
个®×数	42	42	42	42	42								
¥¿态参数a,b	¥­§¡­È	15.349933	9.958388	6.246055	.986319	.575443								
	标­ã®t	11.6036745	7.7271888	4.4449146	.0485000	.3344819								
³ÌÌåºÝ®t­È	绝对	.160	.192	.170	.428	.182								
	¥¿	.160	.192	.170	.389	.160								
	负	-.140	-.146	-.119	-.428	-.182								
检验统计	.160	.192	.170	.428	.182								
渐ªñ显µÛ©Ê¡]双§À¡^	.008c	.000c	.004c	.000c	.001c								

One-sample Kolmogorov-Smirnov test	
	¤ôÊ^DIVISION	¤£³z¤ô­±SPLIT	绿¦aSPLIT	¤ôÊ^SPLIT	¤£³z¤ô­±AI			
个®×数	42	42	42	42	42			
¥¿态参数a,b	¥­§¡­È	1.086931	505897.213354761800000	11.517395	879721.592678571600000	89.266979			
	标­ã®t	1.4649135	3229009.711272453000000	24.7815091	3010803.868616980500000	4.3264209			
³ÌÌåºÝ®t­È	绝对	.500	.530	.336	.403	.111			
	¥¿	.500	.530	.244	.403	.103			
	负	-.299	-.438	-.336	-.385	-.111			
检验统计	.500	.530	.336	.403	.111			
渐ªñ显µÛ©Ê¡]双§À¡^	.000c	.000c	.000c	.000c	.200c,d			

One-sample Kolmogorov-Smirnov test	
	绿¦aAI	¤ôÊ^AI	
个®×数	42	42	
¥¿态参数a,b	¥­§¡­È	97.608474	87.721510	
	标­ã®t	1.4412057	18.4697541	
³ÌÌåºÝ®t­È	绝对	.148	.256	
	¥¿	.070	.256	
	负	-.148	-.213	
检验统计	.148	.256	
渐ªñ显µÛ©Ê¡]双§À¡^	.022c	.000c	

a. The distribution was tested to be normal.	
b. Calculated from the data.	
c.Riley's significance correction.	
d. This is the lower limit of true significance.	
