# Supplementary material for: Optimizing spatial equity of urban park cooling services: Integrating landscape metrics with K-means and PSO algorithms in Nanchang, China
Source: PLoS One. 2026 Mar 19;21(3):e0344026. doi: 10.1371/journal.pone.0344026 (PMC13001981; doi:10.1371/journal.pone.0344026)
Supplement: S1 File — (ZIP) [file pone.0344026.s001.zip › Supplementary material/k-w test and correlation analysis/K-W test and correlation analysis results.pdf]

```
 GET DATA /TYPE=XLSX
 /FILE='D:\          – –       .xlsx'
 /SHEET=name 'Sheet1'
 /CELLRANGE=full
 /READNAMES=on
 /ASSUMEDSTRWIDTH=32767.
EXECUTE.
DATASET NAME       3 WINDOW=FRONT.
DATASET CLOSE       2.
NPAR TESTS
 /K-S(NORMAL)=Shape_Leng Shape_Area
                                PD
 /STATISTICS DESCRIPTIVES
 /MISSING ANALYSIS.
```

N P a r

	19-OCT-2023 14:49:03
	3
	< >
	< >
	< >
	42
	NPAR TESTS<br>/K-S(NORMAL)=Shape_Leng<br>S h a p e _ A r e a<br><br>P D<br>/STATISTICS DESCRIPTIVES<br>/MISSING ANALYSIS.
	00:00:00.00
	00:00:00.01
a	224694

a.

Shape_Leng	42	3473.961925	3422.197397	396.7970767	14451.52283
Shape_Area	42	690997.0792	1493506.980	7963.236683	7753416.988
	42	39.45450600	2.535403553	33.812284	44.095441
	42	423.571	332.3664	30.0	1200.0
	42	1.224692143	.9298081262	.0198000000	3.347500000
	42	23855.071	41505.9990	348.0	192453.0
	42	201122.452	300091.6989	1861.0	1409276.0
	42	201866.571	625575.3981	.0	3473567.0
	42	.0041696836	.0038545403	.0001100000	.0200925000
	42	.0174131446	.0167449146	.0018638908	.0933802254
P D	42	901.250888	665.4919609	52.9998	2428.1792

-

	Shape_Leng	Shape_Area									P D
	42	42	42	42	42	42	42	42	42	42	42
a,b	3473.961925	690997.0792	39.45450600	423.571	1.224692143	23855.071	201122.452	201866.571	.0041696836	.0174131446	901.250888
	3422.197397	1493506.980	2.535403553	332.3664	.9298081262	41505.9990	300091.6989	625575.3981	.0038545403	.0167449146	665.4919609
	.205	.324	.076	.123	.119	.331	.253	.386	.169	.177	.162
	.205	.291	.076	.123	.119	.331	.207	.386	.169	.156	.162
	-.184	-.324	-.069	-.118	-.098	-.286	-.253	-.373	-.146	-.177	-.101
	.205	.324	.076	.123	.119	.331	.253	.386	.169	.177	.162
	.000[c]	.000[c]	.200[c,d]	.110[c]	.149[c]	.000[c]	.000[c]	.000[c]	.004[c]	.002[c]	.007[c]

a.

b.

c.

d.

```
 GET DATA /TYPE=XLSX
 /FILE='D:\ — — .xlsx'
 /SHEET=name 'Sheet1'
 /CELLRANGE=full
 /READNAMES=on
 /ASSUMEDSTRWIDTH=32767.
EXECUTE.
DATASET NAME 4 WINDOW=FRONT.
GET DATA /TYPE=XLSX
 /FILE='D:\ — — .xlsx'
 /SHEET=name 'Sheet1'
 /CELLRANGE=full
 /READNAMES=on
 /ASSUMEDSTRWIDTH=32767.
EXECUTE.
DATASET NAME 5 WINDOW=FRONT.
DATASET CLOSE 3.
DATASET CLOSE 4.
NPAR TESTS
 /K-S(NORMAL)=Shape_Leng Shape_Area
```

```
/STATISTICS DESCRIPTIVES
/MISSING ANALYSIS.
```

N P a r

	19-OCT-2023 15:33:57
	5
	< >
	< >
	< >
	45
	NPAR TESTS<br>/K-S(NORMAL)=Shape_Leng<br>S h a p e _ A r e a<br><br>/STATISTICS DESCRIPTIVES<br>/MISSING ANALYSIS.
	00:00:00.02
	00:00:00.00
a	241979

a.

Shape_Leng	42	3473.961925	3422.197397	396.7970767	14451.52283
Shape_Area	42	690997.0792	1493506.980	7963.236683	7753416.988
	42	39.45450600	2.535403553	33.812284	44.095441
	42	423.571	332.3664	30.0	1200.0
	42	1.224692143	.9298081262	.0198000000	3.347500000
	42	.0041696836	.0038545403	.0001100000	.0200925000
	42	.0174131446	.0167449146	.0018638908	.0933802254
	42	11.63007180	11.58927173	.3740222550	59.13005211
	42	64.27533839	24.50769792	10.94162037	98.06731591
	42	24.01853128	27.16948264	.0000000000	88.00623842

-

	Shape_Leng	Shape_Area								
[a,b]	42	42	42	42	42	42	42	42	42	42
	3473.961925	690997.0792	39.45450600	423.571	1.224692143	.0041696836	.0174131446	11.63007180	64.27533839	24.01853128
	3422.197397	1493506.980	2.535403553	332.3664	.9298081262	.0038545403	.0167449146	11.58927173	24.50769792	27.16948264
	.205	.324	.076	.123	.119	.169	.177	.202	.197	.238
	.205	.291	.076	.123	.119	.169	.156	.202	.085	.238
	-.184	-.324	-.069	-.118	-.098	-.146	-.177	-.166	-.197	-.188
	.205	.324	.076	.123	.119	.169	.177	.202	.197	.238
	.000[c]	.000[c]	.200[c,d]	.110[c]	.149[c]	.004[c]	.002[c]	.000[c]	.000[c]	.000[c]

a.

b.

c.

d.

```
NONPAR CORR
/VARIABLES= Shape_Leng
/PRINT= SPEARMAN TWOTAIL NOSIG
/MISSING= PAIRWISE .
```

	19-OCT-2023 15:36:34
	5
	< >
	< >
	< >
	45
	NONPAR CORR<br>/VARIABLES=Shape_Leng<br><br>/PRINT=SPEARMAN TWOTAIL NOSIG<br>/MISSING=PAIRWISE.
	00:00:00.00
	00:00:00.00
	6 2 9 1 4 5 [a]

a.

		Shape_Leng	
R h o	Shape_Leng	1.000	-.577**
		.	.000
		42	42
		-.577**	1.000
		.000	.
		42	42

**. 0 . 0 1

```
NONPAR CORR
/VARIABLES=              Shape_Area
/PRINT= SPEARMAN TWOTAIL NOSIG
/MISSING= PAIRWISE .
```

	19-OCT-2023 15:37:41
	5
	< >
	< >
	< >
	45
	NONPAR CORR<br>/ V A R I A B L E S =<br>Shape_Area<br>/PRINT=SPEARMAN TWOTAIL NOSIG<br>/MISSING=PAIRWISE.
	00:00:00.02
	00:00:00.00
	6 2 9 1 4 5 [a]

a.

			Shape_Area
R h o		1.000	-.597**
		.	.000
		42	42
	Shape_Area	-.597**	1.000
		.000	.
		42	42

**. 0 . 0 1

```
NONPAR CORR
/VARIABLES=
/PRINT= SPEARMAN TWOTAIL NOSIG
/MISSING= PAIRWISE .
```

	19-OCT-2023 15:38:47
	5
	< >
	< >
	< >
	45
	NONPAR CORR<br>/ V A R I A B L E S =<br><br>/PRINT=SPEARMAN TWOTAIL NOSIG<br>/MISSING=PAIRWISE.
	00:00:00.00
	00:00:00.00
	6 2 9 1 4 5 [a]

a.

R h o	1.000	.584**
	.	.000
	42	42
	.584**	1.000
	.000	.
	42	42

**. 0 . 0 1

```
CORRELATIONS
/VARIABLES=Shape_Leng Shape_Area
/PRINT=TWOTAIL NOSIG
/MISSING=PAIRWISE.
```

	19-OCT-2023 15:40:00
	5
	< >
	< >
	< >
	45
	CORRELATIONS<br>/VARIABLES=Shape_Leng<br>Shape_Area<br>/PRINT=TWOTAIL NOSIG<br>/MISSING=PAIRWISE.
	00:00:00.02
	00:00:00.00

	Shape_Leng	Shape_Area
Shape_Leng	1	.824**
		.000
	42	42
Shape_Area	.824**	1
	.000	
	42	42

**. 0 . 0 1

```
CORRELATIONS
/VARIABLES= Shape_Area
/PRINT= TWOTAIL NOSIG
/MISSING= PAIRWISE.
```

	19-OCT-2023 15:41:27
	5
	< >
	< >
	< >
	45
	CORRELATIONS<br>/VARIABLES=Shape_Area<br>/PRINT=TWOTAIL NOSIG<br>/MISSING=PAIRWISE.
	00:00:00.02
	00:00:00.00

	Shape_Area	
Shape_Area	1	-.351*
		.023
	42	42
	-.351*	1
	.023	
	42	42

*. 0 . 0 5

```
NONPAR CORR
/VARIABLES= Shape_Area
/PRINT= SPEARMAN TWOTAIL NOSIG
/MISSING= PAIRWISE .
```

	19-OCT-2023 15:42:12
	5
	< >
	< >
	< >
	45
	NONPAR CORR<br>/VARIABLES=Shape_Area<br><br>/PRINT=SPEARMAN TWOTAIL NOSIG<br>/MISSING=PAIRWISE.
	00:00:00.00
	00:00:00.00
	6 2 9 1 4 5 [a]

a.

		Shape_Area	
R h o	Shape_Area	1.000	-.337*
		.	.029
		42	42
		-.337*	1.000
		.029	.
		42	42

*. 0 . 0 5

```
NONPAR CORR
/VARIABLES= Shape_Area
/PRINT= SPEARMAN TWOTAIL NOSIG
/MISSING= PAIRWISE .
```

	19-OCT-2023 15:42:53
	5
	< >
	< >
	< >
	45
	NONPAR CORR<br>/VARIABLES=Shape_Area<br><br>/PRINT=SPEARMAN TWOTAIL NOSIG<br>/MISSING=PAIRWISE.
	00:00:00.00
	00:00:00.00
	6 2 9 1 4 5 [a]

a.

		Shape_Area	
R h o	Shape_Area	1.000	.340*
		.	.028
		42	42
		.340*	1.000
		.028	.
		42	42

*. 0 . 0 5

```
CORRELATIONS
/VARIABLES= Shape_Area
/PRINT= TWOTAIL NOSIG
/MISSING= PAIRWISE .
```

	19-OCT-2023 15:48:39
	5
	< >
	< >
	< >
	45
	CORRELATIONS<br>/VARIABLES=Shape_Area<br><br>/PRINT=TWOTAIL NOSIG<br>/MISSING=PAIRWISE.
	00:00:00.00
	00:00:00.01

	Shape_Area	
Shape_Area	1<br><br>42	-.232<br>.140<br>42
	-.232<br>.140<br>42	1<br><br>42

```
CORRELATIONS
/VARIABLES= Shape_Area
/PRINT= TWOTAIL NOSIG
/MISSING= PAIRWISE .
```

	19-OCT-2023 15:49:09<br><br>5<br>< ><br>< ><br>< ><br>45<br><br>CORRELATIONS<br>/VARIABLES=Shape_Area<br><br>/PRINT=TWOTAIL NOSIG<br>/MISSING=PAIRWISE.<br>00:00:00.02<br>00:00:00.01

	Shape_Area	
Shape_Area	1<br><br>42	-.242<br>.123<br>42
	-.242<br>.123<br>42	1<br><br>42

```
CORRELATIONS
/VARIABLES=Shape_Area
/PRINT=TWOTAIL NOSIG
/MISSING=PAIRWISE.
```

	19-OCT-2023 15:49:33
	5
	< >
	< >
	< >
	45
	CORRELATIONS /VARIABLES=Shape_Area /PRINT=TWOTAIL NOSIG /MISSING=PAIRWISE.
	00:00:00.00
	00:00:00.00

	Shape_Area	
Shape_Area	1	-.332*
		.032
	42	42
	-.332*	1
	.032	
	42	42

*. 0 . 0 5


```
CORRELATIONS
/VARIABLES=Shape_Area
/PRINT=TWOTAIL NOSIG
/MISSING=PAIRWISE.
```

	19-OCT-2023 15:49:55
	5
	< >
	< >
	< >
	45
	CORRELATIONS<br>/VARIABLES=Shape_Area<br><br>/PRINT=TWOTAIL NOSIG<br>/MISSING=PAIRWISE.
	00:00:00.02
	00:00:00.01

	Shape_Area	
Shape_Area	1	.408**
		.007
	42	42
	.408**	1
	.007	
	42	42

**. 0 . 0 1

```
CORRELATIONS
/VARIABLES= Shape_Leng
/PRINT= TWOTAIL NOSIG
/MISSING= PAIRWISE .
```

	19-OCT-2023 15:50:48
	5
	< >
	< >
	< >
	45
	CORRELATIONS<br>/VARIABLES=Shape_Leng<br><br>/PRINT=TWOTAIL NOSIG<br>/MISSING=PAIRWISE.
	00:00:00.00
	00:00:00.00

	Shape_Leng	
Shape_Leng	1	-.464**
		.002
	42	42
	-.464**	1
	.002	
	42	42

**. 0 . 0 1

```
NONPAR CORR
/VARIABLES= Shape_Leng
/PRINT= SPEARMAN TWOTAIL NOSIG
/MISSING= PAIRWISE .
```

	19-OCT-2023 15:51:16
	5
	< >
	< >
	< >
	45
	NONPAR CORR<br>/VARIABLES=Shape_Leng<br><br>/PRINT=SPEARMAN TWOTAIL NOSIG<br>/MISSING=PAIRWISE.
	00:00:00.00
	00:00:00.00
	6 2 9 1 4 5 [a]

a.

		Shape_Leng	
R h o	Shape_Leng	1.000	-.050
		.	.756
		42	42
		-.050	1.000
		.756	.
		42	42

```
NONPAR CORR
/VARIABLES= Shape_Leng
/PRINT= SPEARMAN TWOTAIL NOSIG
/MISSING= PAIRWISE .
```

	19-OCT-2023 15:51:47
	5
	< >
	< >
	< >
	45
	NONPAR CORR<br>/VARIABLES=Shape_Leng<br><br>/PRINT=SPEARMAN TWOTAIL NOSIG<br>/MISSING=PAIRWISE.
	00:00:00.02
	00:00:00.00
	6 2 9 1 4 5 [a]

a.

		Shape_Leng	
R h o	Shape_Leng	1.000	.333*
		.	.031
		42	42
		.333*	1.000
		.031	.
		42	42

*. 0 . 0 5

```
CORRELATIONS
/VARIABLES= Shape_Leng
/PRINT= TWOTAIL NOSIG
/MISSING= PAIRWISE .
```

	19-OCT-2023 15:52:17
	5
	< >
	< >
	< >
	45
	CORRELATIONS /VARIABLES=Shape_Leng /PRINT=TWOTAIL NOSIG /MISSING=PAIRWISE.
	00:00:00.00
	00:00:00.01

	Shape_Leng	
Shape_Leng	1	-.299
		.055
	42	42
	-.299	1
	.055	
	42	42

```
CORRELATIONS
/VARIABLES=Shape_Leng
/PRINT=TWOTAIL NOSIG
/MISSING=PAIRWISE.
```

	19-OCT-2023 15:52:36
	5
	< >
	< >
	< >
	45
	CORRELATIONS<br>/VARIABLES=Shape_Leng<br><br>/PRINT=TWOTAIL NOSIG<br>/MISSING=PAIRWISE.
	00:00:00.02
	00:00:00.01

	Shape_Leng	
Shape_Leng	1	-.309*
		.047
	42	42
	-.309*	1
	.047	
	42	42

*. 0 . 0 5

```
CORRELATIONS
/VARIABLES= Shape_Leng
/PRINT= TWOTAIL NOSIG
/MISSING= PAIRWISE .
```

	19-OCT-2023 15:52:55
	5
	< >
	< >
	< >
	45
	CORRELATIONS /VARIABLES=Shape_Leng /PRINT=TWOTAIL NOSIG /MISSING=PAIRWISE.
	00:00:00.00
	00:00:00.00

	Shape_Leng	
Shape_Leng	1	-.281
		.071
	42	42
	-.281	1
	.071	
	42	42

```
CORRELATIONS
/VARIABLES=Shape_Leng
/PRINT=TWOTAIL NOSIG
/MISSING=PAIRWISE.
```

	19-OCT-2023 15:53:11
	5
	< >
	< >
	< >
	45
	CORRELATIONS<br>/VARIABLES=Shape_Leng<br><br>/PRINT=TWOTAIL NOSIG<br>/MISSING=PAIRWISE.
	00:00:00.02
	00:00:00.00

	Shape_Leng	
Shape_Leng	1	.392*
		.010
	42	42
	.392*	1
	.010	
	42	42

*. 0 . 0 5

```
NONPAR CORR
/VARIABLES=
/PRINT= SPEARMAN TWOTAIL NOSIG
/MISSING= PAIRWISE .
```

	19-OCT-2023 15:53:58
	5
	< >
	< >
	< >
	45
	NONPAR CORR<br>/ V A R I A B L E S =<br>/PRINT=SPEARMAN TWOTAIL NOSIG<br>/MISSING=PAIRWISE.
	00:00:00.00
	00:00:00.00
	6 2 9 1 4 5 [a]

a.

R h o		1.000	.077
		.	.630
		42	42
		.077	1.000
		.630	.
		42	42

```
NONPAR CORR
/VARIABLES=
/PRINT= SPEARMAN TWOTAIL NOSIG
/MISSING= PAIRWISE .
```

	19-OCT-2023 15:54:16
	5
	< >
	< >
	< >
	45
	NONPAR CORR<br>/ V A R I A B L E S =<br>/PRINT=SPEARMAN TWOTAIL NOSIG<br>/MISSING=PAIRWISE.
	00:00:00.00
	00:00:00.00
	6 2 9 1 4 5 [a]

a.

R h o	1.000	-.276
	.	.077
	42	42
	-.276	1.000
	.077	.
	42	42

```
CORRELATIONS
/VARIABLES=
/PRINT= TWOTAIL NOSIG
/MISSING= PAIRWISE.
```

	19-OCT-2023 15:54:32
	5
	< >
	< >
	< >
	45
	CORRELATIONS<br>/ V A R I A B L E S =<br>/PRINT=TWOTAIL NOSIG<br>/MISSING=PAIRWISE.
	00:00:00.00
	00:00:00.01

	1	.314*
		.043
	42	42
	.314*	1
	.043	
	42	42

*. 0 . 0 5

```
CORRELATIONS
/VARIABLES=
/PRINT= TWOTAIL NOSIG
/MISSING= PAIRWISE .
```

	19-OCT-2023 15:54:54
	5
	< >
	< >
	< >
	45
	CORRELATIONS<br>/ V A R I A B L E S =<br><br>/PRINT=TWOTAIL NOSIG<br>/MISSING=PAIRWISE.
	00:00:00.00
	00:00:00.00

	1	.784**
		.000
	42	42
	.784**	1
	.000	
	42	42

**. 0 . 0 1

```
CORRELATIONS
/VARIABLES=
/PRINT=TWOTAIL NOSIG
/MISSING=PAIRWISE.
```

	19-OCT-2023 15:55:13
	5
	< >
	< >
	< >
	45
	CORRELATIONS<br>/ V A R I A B L E S =<br>/PRINT=TWOTAIL NOSIG<br>/MISSING=PAIRWISE.
	00:00:00.03
	00:00:00.01

	1	-.058
		.714
	42	42
	-.058	1
	.714	
	42	42

```
CORRELATIONS
/VARIABLES=
/PRINT= TWOTAIL NOSIG
/MISSING= PAIRWISE .
```

	19-OCT-2023 15:55:27
	5
	< >
	< >
	< >
	45
	CORRELATIONS<br>/ V A R I A B L E S =<br>/PRINT=TWOTAIL NOSIG<br>/MISSING=PAIRWISE.
	00:00:00.00
	00:00:00.00

	1	-.299
		.054
	42	42
	-.299	1
	.054	
	42	42

```
NONPAR CORR
/VARIABLES=
/PRINT= SPEARMAN TWOTAIL NOSIG
/MISSING= PAIRWISE .
```

	19-OCT-2023 15:56:03
	5
	< >
	< >
	< >
	45
	NONPAR CORR<br>/ V A R I A B L E S =<br><br>/PRINT=SPEARMAN TWOTAIL NOSIG<br>/MISSING=PAIRWISE.
	00:00:00.00
	00:00:00.00
	6 2 9 1 4 5 [a]

a.

R h o		1.000	-.154
		.	.330
		42	42
		-.154	1.000
		.330	.
		42	42

	19-OCT-2023 15:57:13
	5
	< >
	< >
	< >
	45
	CORRELATIONS<br>/ V A R I A B L E S =<br><br>/PRINT=TWOTAIL NOSIG<br>/MISSING=PAIRWISE.
	00:00:00.00
	00:00:00.00

	19-OCT-2023 15:57:13
	5
	< >
	< >
	< >
	45
	NONPAR CORR<br>/ V A R I A B L E S =<br><br>/PRINT=SPEARMAN TWOTAIL NOSIG<br>/MISSING=PAIRWISE.
	00:00:00.00
	00:00:00.00
	6 2 9 1 4 5 [a]

a.

```
NONPAR CORR
/VARIABLES=
/PRINT= SPEARMAN TWOTAIL NOSIG
/MISSING= PAIRWISE .
```

	19-OCT-2023 15:57:43
	5
	< >
	< >
	< >
	45
	NONPAR CORR<br>/ V A R I A B L E S =<br><br>/PRINT=SPEARMAN TWOTAIL NOSIG<br>/MISSING=PAIRWISE.
	00:00:00.00
	00:00:00.00
	6 2 9 1 4 5 [a]

a.

R h o		1.000	-.133
		.	.401
		42	42
		-.133	1.000
		.401	.
		42	42

```
NONPAR CORR
/VARIABLES=
/PRINT=SPEARMAN TWOTAIL NOSIG
/MISSING=PAIRWISE.
```

	19-OCT-2023 15:58:08
	5
	< >
	< >
	< >
	45
	NONPAR CORR<br>/ V A R I A B L E S =<br><br>/PRINT=SPEARMAN TWOTAIL NOSIG<br>/MISSING=PAIRWISE.
	00:00:00.00
	00:00:00.00
	6 2 9 1 4 5 [a]

a.

R h o	1.000	-.021
	.	.895
	42	42
	-.021	1.000
	.895	.
	42	42

```
NONPAR CORR
/VARIABLES=
/PRINT=SPEARMAN TWOTAIL NOSIG
/MISSING=PAIRWISE.
```

	19-OCT-2023 15:58:23
	5
	< >
	< >
	< >
	45
	NONPAR CORR<br>/ V A R I A B L E S =<br><br>/PRINT=SPEARMAN TWOTAIL NOSIG<br>/MISSING=PAIRWISE.
	00:00:00.00
	00:00:00.00
	6 2 9 1 4 5 [a]

a.

R h o	1.000	.692**
	.	.000
	42	42
	.692**	1.000
	.000	.
	42	42

**. 0 . 0 1

```
NONPAR CORR
/VARIABLES=
/PRINT= SPEARMAN TWOTAIL NOSIG
/MISSING= PAIRWISE.
```

	19-OCT-2023 15:58:44
	5
	< >
	< >
	< >
	45
	NONPAR CORR<br>/ V A R I A B L E S =<br><br>/PRINT=SPEARMAN TWOTAIL NOSIG<br>/MISSING=PAIRWISE.
	00:00:00.00
	00:00:00.00
	6 2 9 1 4 5 [a]

a.

R h o	1.000	.199
	.	.206
	42	42
	.199	1.000
	.206	.
	42	42

```
NONPAR CORR
/VARIABLES=
/PRINT=SPEARMAN TWOTAIL NOSIG
/MISSING=PAIRWISE.
```

	19-OCT-2023 15:59:10
	5
	< >
	< >
	< >
	45
	NONPAR CORR<br>/ V A R I A B L E S =<br><br>/PRINT=SPEARMAN TWOTAIL NOSIG<br>/MISSING=PAIRWISE.
	00:00:00.02
	00:00:00.00
	6 2 9 1 4 5 [a]

a.

R h o		1.000	-.560**
		.	.000
		42	42
		-.560**	1.000
		.000	.
		42	42

**. 0 . 0 1

```
NONPAR CORR
/VARIABLES=
/PRINT= SPEARMAN TWOTAIL NOSIG
/MISSING= PAIRWISE .
```

	19-OCT-2023 15:59:48
	5
	< >
	< >
	< >
	45
	NONPAR CORR<br>/ V A R I A B L E S =<br>/PRINT=SPEARMAN TWOTAIL NOSIG<br>/MISSING=PAIRWISE.
	00:00:00.00
	00:00:00.00
	6 2 9 1 4 5 [a]

a.

R h o	1.000	.420**
	.	.006
	42	42
	.420**	1.000
	.006	.
	42	42

**. 0 . 0 1

```
NONPAR CORR
/VARIABLES=
/PRINT= SPEARMAN TWOTAIL NOSIG
/MISSING= PAIRWISE.
```

	19-OCT-2023 16:00:13
	5
	< >
	< >
	< >
	45
	NONPAR CORR<br>/ V A R I A B L E S =<br>/PRINT=SPEARMAN TWOTAIL NOSIG<br>/MISSING=PAIRWISE.
	00:00:00.00
	00:00:00.00
	6 2 9 1 4 5 [a]

a.

R h o	1.000	.558**
	.	.000
	42	42
	.558**	1.000
	.000	.
	42	42

**. 0 . 0 1

```
NONPAR CORR
/VARIABLES=
/PRINT= SPEARMAN TWOTAIL NOSIG
/MISSING= PAIRWISE .
```

	19-OCT-2023 16:00:37
	5
	< >
	< >
	< >
	45
	NONPAR CORR<br>/ V A R I A B L E S =<br><br>/PRINT=SPEARMAN TWOTAIL NOSIG<br>/MISSING=PAIRWISE.
	00:00:00.00
	00:00:00.00
	6 2 9 1 4 5 [a]

a.

R h o	1.000	-.044
	.	.784
	42	42
	-.044	1.000
	.784	.
	42	42

```
NONPAR CORR
/VARIABLES=
/PRINT=SPEARMAN TWOTAIL NOSIG
/MISSING=PAIRWISE.
```

	19-OCT-2023 16:00:56
	5
	< >
	< >
	< >
	45
	NONPAR CORR<br>/ V A R I A B L E S =<br>/PRINT=SPEARMAN TWOTAIL NOSIG<br>/MISSING=PAIRWISE.
	00:00:00.00
	00:00:00.00
	6 2 9 1 4 5 [a]

a.

R h o	1.000	-.188
	.	.234
	42	42
	-.188	1.000
	.234	.
	42	42

```
NONPAR CORR
/VARIABLES=
/PRINT=SPEARMAN TWOTAIL NOSIG
/MISSING=PAIRWISE.
```

	19-OCT-2023 16:01:11
	5
	< >
	< >
	< >
	45
	NONPAR CORR<br>/ V A R I A B L E S =<br>/PRINT=SPEARMAN TWOTAIL NOSIG<br>/MISSING=PAIRWISE.
	00:00:00.00
	00:00:00.00
	6 2 9 1 4 5 [a]

a.

R h o		1.000	.247
		.	.115
		42	42
		.247	1.000
		.115	.
		42	42

```
NONPAR CORR
/VARIABLES=
/PRINT=SPEARMAN TWOTAIL NOSIG
/MISSING=PAIRWISE.
```

	19-OCT-2023 16:02:09
	5
	< >
	< >
	< >
	45
	NONPAR CORR<br>/ V A R I A B L E S =<br>/PRINT=SPEARMAN TWOTAIL NOSIG<br>/MISSING=PAIRWISE.
	00:00:00.00
	00:00:00.00
	6 2 9 1 4 5 [a]

a.

R h o	1.000	-.462**
	.	.002
	42	42
	-.462**	1.000
	.002	.
	42	42

**. 0 . 0 1

```
NONPAR CORR
/VARIABLES=
/PRINT= SPEARMAN TWOTAIL NOSIG
/MISSING= PAIRWISE.
```

	19-OCT-2023 16:02:33
	5
	< >
	< >
	< >
	45
	NONPAR CORR<br>/ V A R I A B L E S =<br><br>/PRINT=SPEARMAN TWOTAIL NOSIG<br>/MISSING=PAIRWISE.
	00:00:00.00
	00:00:00.00
	6 2 9 1 4 5 [a]

a.

R h o	1.000	-.208
	.	.185
	42	42
	-.208	1.000
	.185	.
	42	42

```
NONPAR CORR
/VARIABLES=
/PRINT=SPEARMAN TWOTAIL NOSIG
/MISSING=PAIRWISE.
```

	19-OCT-2023 16:02:56
	5
	< >
	< >
	< >
	45
	NONPAR CORR<br>/ V A R I A B L E S =<br>/PRINT=SPEARMAN TWOTAIL NOSIG<br>/MISSING=PAIRWISE.
	00:00:00.00
	00:00:00.00
	6 2 9 1 4 5 [a]

a.

R h o	1.000	-.104
	.	.513
	42	42
	-.104	1.000
	.513	.
	42	42

```
NONPAR CORR
/VARIABLES=
/PRINT=SPEARMAN TWOTAIL NOSIG
/MISSING=PAIRWISE.
```

	19-OCT-2023 16:03:11
	5
	< >
	< >
	< >
	45
	NONPAR CORR<br>/ V A R I A B L E S =<br>/PRINT=SPEARMAN TWOTAIL NOSIG<br>/MISSING=PAIRWISE.
	00:00:00.00
	00:00:00.00
	6 2 9 1 4 5 [a]

a.

R h o	1.000	.255
	.	.103
	42	42
	.255	1.000
	.103	.
	42	42

```
CORRELATIONS
/VARIABLES=
/PRINT=TWOTAIL NOSIG
/MISSING=PAIRWISE.
```

	19-OCT-2023 16:33:45
	5
	< >
	< >
	< >
	45
	CORRELATIONS<br>/ V A R I A B L E S =<br><br>/PRINT=TWOTAIL NOSIG<br>/MISSING=PAIRWISE.
	00:00:00.03
	00:00:00.01

	1	.052
		.745
	42	42
	.052	1
	.745	
	42	42

```
CORRELATIONS
/VARIABLES=
/PRINT= TWOTAIL NOSIG
/MISSING= PAIRWISE.
```

	19-OCT-2023 16:34:04
	5
	< >
	< >
	< >
	45
	CORRELATIONS<br>/ V A R I A B L E S =<br>/PRINT=TWOTAIL NOSIG<br>/MISSING=PAIRWISE.
	00:00:00.02
	00:00:00.00

	1	-.096
		.544
	42	42
	-.096	1
	.544	
	42	42

```
CORRELATIONS
/VARIABLES=
/PRINT= TWOTAIL NOSIG
/MISSING= PAIRWISE .
```

	19-OCT-2023 16:34:18
	5
	< >
	< >
	< >
	45
	CORRELATIONS<br>/ V A R I A B L E S =<br>/PRINT=TWOTAIL NOSIG<br>/MISSING=PAIRWISE.
	00:00:00.03
	00:00:00.00

	1	.064
		.688
	42	42
	.064	1
	.688	
	42	42

```
CORRELATIONS
/VARIABLES=
/PRINT= TWOTAIL NOSIG
/MISSING= PAIRWISE .
```

	19-OCT-2023 16:35:46
	5
	< >
	< >
	< >
	45
	CORRELATIONS<br>/ V A R I A B L E S =<br><br>/PRINT=TWOTAIL NOSIG<br>/MISSING=PAIRWISE.
	00:00:00.00
	00:00:00.01

	1	-.008
		.961
	42	42
	-.008	1
	.961	
	42	42

```
CORRELATIONS
/VARIABLES=
/PRINT= TWOTAIL NOSIG
/MISSING= PAIRWISE .
```

	19-OCT-2023 16:36:04
	5
	< >
	< >
	< >
	45
	CORRELATIONS<br>/VARIABLES=<br><br>/PRINT=TWOTAIL NOSIG<br>/MISSING=PAIRWISE.
	00:00:00.00
	00:00:00.00

	1	-.435**
		.004
	42	42
	-.435**	1
	.004	
	42	42

**. 0 . 0 1

```
CORRELATIONS
/VARIABLES=
/PRINT=TWOTAIL NOSIG
/MISSING=PAIRWISE.
```

	19-OCT-2023 16:36:27
	5
	< >
	< >
	< >
	45
	CORRELATIONS<br>/ V A R I A B L E S =<br><br>/PRINT=TWOTAIL NOSIG<br>/MISSING=PAIRWISE.
	00:00:00.05
	00:00:00.00

	1	-.897**
		.000
	42	42
	-.897**	1
	.000	
	42	42

**. 0 . 0 1
