# Supplementary material for: Optimizing spatial equity of urban park cooling services: Integrating landscape metrics with K-means and PSO algorithms in Nanchang, China
Source: PLoS One. 2026 Mar 19;21(3):e0344026. doi: 10.1371/journal.pone.0344026 (PMC13001981; doi:10.1371/journal.pone.0344026)
Supplement: S1 File — (ZIP) [file pone.0344026.s001.zip › Supplementary material/k-w test and correlation analysis/Pearseman analysis for all indicators.doc]

GET
  FILE='D:\ACCESSIBILITY AND PCI\SPSS\SPSS¥X图\无标题6.sav'.
DATASET NAME 数Õu¶°1 WINDOW=FRONT.
NPAR TESTS
  /K-S(NORMAL)=¥­§¡¦aªí温«× ­°温­S围 ­°温´T«× ­°温±è«× ¿Ä¦X«ü数 ¤£³z¤ô­±ÂÐ盖²v 绿¤ÆÂÐ盖²v ¤ôÊ^ÂÐ盖²v(Mean surface temperature Cooling range Cooling range Cooling gradient Convergence index Impervious surface cover Green cover Water cover)
  /STATISTICS DESCRIPTIVES
  /MISSING ANALYSIS.


NPar 检验


备ª`	
¤w创«Ø输¥X	23-OCT-2023 11:29:22	
ª`释		
输¤J	数Õu	D:\ACCESSIBILITY AND PCI\SPSS\SPSS¥X图\无标题6.sav	
	¬¡动数Õu¶°	数Õu¶°1	
	过滤¾¹	<无>	
	权­«	<无>	
	©î¤À¤å¥ó	<无>	
	¤u§@数Õu¤å¥ó¤¤ªº¦æ数	45	
¯Ê¥¢­È处²z	对¯Ê¥¢ªº©w义	将¥Î户©w义ªº¯Ê¥¢­È视为¯Ê¥¢¡C	
	¨Ï¥Îªº个®×数	¨C项检验ªº统计³£°ò¤_©Ò¦³对¤_该检验¤¤¨Ï¥Îªº变¶q¨ã¦³¦³®Ä数Õuªº个®×¡C	
语ªk	NPAR TESTS
  /K-S(NORMAL)=¥­§¡¦aªí温«× ­°温­S围 ­°温´T«× ­°温±è«× ¿Ä¦X«ü数 ¤£³z¤ô­±ÂÐ盖²v 绿¤ÆÂÐ盖²v ¤ôÊ^ÂÐ盖²v
  /STATISTICS DESCRIPTIVES
  /MISSING ANALYSIS.	
资·½	处²zµ{§Ç时间	00:00:00.02	
	¯Ó¥Î时间	00:00:00.01	
	¤¹许ªº个®×数a	285975	

a. °ò¤_¤u§@ªÅ间内¦sªº¥i¥Î©Ê¡C	


[数Õu¶°1] D:\ACCESSIBILITY AND PCI\SPSS\SPSS¥X图\无标题6.sav


descriptive statistics	
	个®×数	¥­§¡­È	标­ã®t	³Ì¤p­È	³Ì¤j­È	
¥­§¡¦aªí温«×	42	39.45450600	2.535403553	33.812284	44.095441	
­°温­S围	42	423.571	332.3664	30.0	1200.0	
­°温´T«×	42	1.224692142857143	.929808126232231	.0197999999999965	3.3475000000000000	
­°温±è«×	42	.004169683585892	.003854540328412	.0001100000000000	.0200925000000000	
¿Ä¦X«ü数	42	.017413144621368	.016744914647473	.0018638908299955	.0933802254396813	
¤£³z¤ô­±ÂÐ盖²v	42	11.630071799195628	11.589271732568069	.37402225503349	59.13005210837930	
绿¤ÆÂÐ盖²v	42	64.275338385688490	24.507697920262252	10.9416203699165	98.0673159131800	
¤ôÊ^ÂÐ盖²v	42	24.018531276102816	27.169482643623535	.000000000000000	88.006238424195700	


One-sample Kolmogorov-Sminov test	
	¥­§¡¦aªí温«×	­°温­S围	­°温´T«×	­°温±è«×					
个®×数	42	42	42	42					
¥¿态参数a,b	¥­§¡­È	39.45450600	423.571	1.224692142857143	.004169683585892					
	标­ã®t	2.535403553	332.3664	.929808126232231	.003854540328412					
³ÌÌåºÝ®t­È	绝对	.076	.123	.119	.169					
	¥¿	.076	.123	.119	.169					
	负	-.069	-.118	-.098	-.146					
检验统计	.076	.123	.119	.169					
渐ªñ显µÛ©Ê¡]双§À¡^	.200c,d	.110c	.149c	.004c					

One-sample Kolmogorov-Sminov test	
	¿Ä¦X«ü数	¤£³z¤ô­±ÂÐ盖²v	绿¤ÆÂÐ盖²v	¤ôÊ^ÂÐ盖²v	
个®×数	42	42	42	42	
¥¿态参数a,b	¥­§¡­È	.017413144621368	11.630071799195630	64.275338385688490	24.018531276102816	
	标­ã®t	.016744914647473	11.589271732568070	24.507697920262235	27.169482643623535	
³ÌÌåºÝ®t­È	绝对	.177	.202	.197	.238	
	¥¿	.156	.202	.085	.238	
	负	-.177	-.166	-.197	-.188	
检验统计	.177	.202	.197	.238	
渐ªñ显µÛ©Ê¡]双§À¡^	.002c	.000c	.000c	.000c	

a. 检验¤À¥¬为¥¿态¤À¥¬¡C	
b. ®ÚÕu数Õu计ºâ¡C	
c. ¨½§Q¤ó显µÛ©Ê­×¥¿¡C	
d. 这¬O¯u显µÛ©Êªº¤U­­¡C	

NPAR TESTS
  /K-S(NORMAL)=¥­§¡¦aªí温«× ­°温­S围 ­°温´T«× ­°温±è«× ¤£³z¤ô­±PD 绿¦aPD ¤ôÊ^PD ¤£³z¤ô­±LPI 绿¦aLPI ¤ôÊ^LPI ¤£³z¤ô­±LSI 绿¦aLSI ¤ôÊ^LSI
    ¤£³z¤ô­±DIVISION 绿¦aDIVISION ¤ôÊ^DIVISION ¤£³z¤ô­±SPLIT 绿¦aSPLIT ¤ôÊ^SPLIT ¤£³z¤ô­±AI 绿¦aAI ¤ôÊ^AI(Mean Surface Temperature Cooling Range Cooling Range Cooling Gradient Impervious Surface PD Greenfield PD Waterbody PD Impervious Surface LPI Greenfield LPI Waterbody LPI Impervious Surface LSI Greenfield LSI Waterbody LSI
    Impervious Surface DIVISION Greenfield DIVISION Waterbody DIVISION Impervious Surface SPLIT Greenfield SPLIT Waterbody SPLIT Impervious Surface AI Greenfield AI Waterbody AI)
  /STATISTICS DESCRIPTIVES
  /MISSING ANALYSIS.


NPar 检验


备ª`	
¤w创«Ø输¥X	23-OCT-2023 12:38:42	
ª`释		
输¤J	数Õu	D:\ACCESSIBILITY AND PCI\SPSS\SPSS¥X图\无标题6.sav	
	¬¡动数Õu¶°	数Õu¶°1	
	过滤¾¹	<无>	
	权­«	<无>	
	©î¤À¤å¥ó	<无>	
	¤u§@数Õu¤å¥ó¤¤ªº¦æ数	45	
¯Ê¥¢­È处²z	对¯Ê¥¢ªº©w义	将¥Î户©w义ªº¯Ê¥¢­È视为¯Ê¥¢¡C	
	¨Ï¥Îªº个®×数	¨C项检验ªº统计³£°ò¤_©Ò¦³对¤_该检验¤¤¨Ï¥Îªº变¶q¨ã¦³¦³®Ä数Õuªº个®×¡C	
语ªk	NPAR TESTS
  /K-S(NORMAL)=¥­§¡¦aªí温«× ­°温­S围 ­°温´T«× ­°温±è«× ¤£³z¤ô­±PD 绿¦aPD ¤ôÊ^PD ¤£³z¤ô­±LPI 绿¦aLPI ¤ôÊ^LPI ¤£³z¤ô­±LSI 绿¦aLSI ¤ôÊ^LSI
    ¤£³z¤ô­±DIVISION 绿¦aDIVISION ¤ôÊ^DIVISION ¤£³z¤ô­±SPLIT 绿¦aSPLIT ¤ôÊ^SPLIT ¤£³z¤ô­±AI 绿¦aAI ¤ôÊ^AI
  /STATISTICS DESCRIPTIVES
  /MISSING ANALYSIS.	
资·½	处²zµ{§Ç时间	00:00:00.02	
	¯Ó¥Î时间	00:00:00.01	
	¤¹许ªº个®×数a	125829	

a. °ò¤_¤u§@ªÅ间内¦sªº¥i¥Î©Ê¡C	


´y­z统计	
	个®×数	¥­§¡­È	标­ã®t	³Ì¤p­È	³Ì¤j­È	
¥­§¡¦aªí温«×	42	39.45450600	2.535403553	33.812284	44.095441	
­°温­S围	42	423.571	332.3664	30.0	1200.0	
­°温´T«×	42	1.224692142857143	.929808126232231	.0197999999999965	3.3475000000000000	
­°温±è«×	42	.004169683585892	.003854540328412	.0001100000000000	.0200925000000000	
¤£³z¤ô­±PD	42	901.250888	665.4919609	52.9998	2428.1792	
绿¦aPD	42	578.900281	564.9157815	9.4642	3294.8929	
¤ôÊ^PD	42	367.848045	416.6902729	.0000	2335.2625	
¤£³z¤ô­±LPI	42	5.288400	10.0370544	.0165	47.2158	
绿¦aLPI	42	56.391929	31.3454044	7.2552	98.2312	
¤ôÊ^LPI	42	22.018762	27.8812483	.0000	87.9389	
¤£³z¤ô­±LSI	42	15.349933	11.6036745	2.7917	53.5470	
绿¦aLSI	42	9.958388	7.7271888	1.8224	31.9715	
¤ôÊ^LSI	42	6.246055	4.4449146	.0000	19.8223	
¤£³z¤ô­±DIVISION	42	.986319	.0485000	.7715	1.0000	
绿¦aDIVISION	42	.575443	.3344819	.0351	.9936	
¤ôÊ^DIVISION	42	1.086931	1.4649135	.0000	10.2404	
¤£³z¤ô­±SPLIT	42	505897.213354761900000	3229009.711272453000000	4.3761000000000	20933799.5849000000000	
绿¦aSPLIT	42	11.517395	24.7815091	1.0363	156.6127	
¤ôÊ^SPLIT	42	879721.592678571400000	3010803.868616980500000	.0000000000000	16754861.5538000000000	
¤£³z¤ô­±AI	42	89.266979	4.3264209	71.3743	97.1928	
绿¦aAI	42	97.608474	1.4412057	92.6843	99.9192	
¤ôÊ^AI	42	87.721510	18.4697541	.0000	99.8612	


单样¥»¬_尔²ö¤à¬¥¤Ò-´µ¦Ì诺¤Ò检验	
	¥­§¡¦aªí温«×	­°温­S围	­°温´T«×	­°温±è«×	¤£³z¤ô­±PD																		
个®×数	42	42	42	42	42																		
¥¿态参数a,b	¥­§¡­È	39.45450600	423.571	1.224692142857143	.004169683585892	901.250888																		
	标­ã®t	2.535403553	332.3664	.929808126232231	.003854540328412	665.4919609																		
³ÌÌåºÝ®t­È	绝对	.076	.123	.119	.169	.162																		
	¥¿	.076	.123	.119	.169	.162																		
	负	-.069	-.118	-.098	-.146	-.101																		
检验统计	.076	.123	.119	.169	.162																		
渐ªñ显µÛ©Ê¡]双§À¡^	.200c,d	.110c	.149c	.004c	.007c																		

单样¥»¬_尔²ö¤à¬¥¤Ò-´µ¦Ì诺¤Ò检验	
	绿¦aPD	¤ôÊ^PD	¤£³z¤ô­±LPI	绿¦aLPI	¤ôÊ^LPI													
个®×数	42	42	42	42	42													
¥¿态参数a,b	¥­§¡­È	578.900281	367.848045	5.288400	56.391929	22.018762													
	标­ã®t	564.9157815	416.6902729	10.0370544	31.3454044	27.8812483													
³ÌÌåºÝ®t­È	绝对	.165	.197	.309	.198	.245													
	¥¿	.165	.197	.309	.155	.245													
	负	-.157	-.189	-.300	-.198	-.215													
检验统计	.165	.197	.309	.198	.245													
渐ªñ显µÛ©Ê¡]双§À¡^	.006c	.000c	.000c	.000c	.000c													

单样¥»¬_尔²ö¤à¬¥¤Ò-´µ¦Ì诺¤Ò检验	
	¤£³z¤ô­±LSI	绿¦aLSI	¤ôÊ^LSI	¤£³z¤ô­±DIVISION	绿¦aDIVISION								
个®×数	42	42	42	42	42								
¥¿态参数a,b	¥­§¡­È	15.349933	9.958388	6.246055	.986319	.575443								
	标­ã®t	11.6036745	7.7271888	4.4449146	.0485000	.3344819								
³ÌÌåºÝ®t­È	绝对	.160	.192	.170	.428	.182								
	¥¿	.160	.192	.170	.389	.160								
	负	-.140	-.146	-.119	-.428	-.182								
检验统计	.160	.192	.170	.428	.182								
渐ªñ显µÛ©Ê¡]双§À¡^	.008c	.000c	.004c	.000c	.001c								

单样¥»¬_尔²ö¤à¬¥¤Ò-´µ¦Ì诺¤Ò检验	
	¤ôÊ^DIVISION	¤£³z¤ô­±SPLIT	绿¦aSPLIT	¤ôÊ^SPLIT	¤£³z¤ô­±AI			
个®×数	42	42	42	42	42			
¥¿态参数a,b	¥­§¡­È	1.086931	505897.213354761800000	11.517395	879721.592678571600000	89.266979			
	标­ã®t	1.4649135	3229009.711272453000000	24.7815091	3010803.868616980500000	4.3264209			
³ÌÌåºÝ®t­È	绝对	.500	.530	.336	.403	.111			
	¥¿	.500	.530	.244	.403	.103			
	负	-.299	-.438	-.336	-.385	-.111			
检验统计	.500	.530	.336	.403	.111			
渐ªñ显µÛ©Ê¡]双§À¡^	.000c	.000c	.000c	.000c	.200c,d			

单样¥»¬_尔²ö¤à¬¥¤Ò-´µ¦Ì诺¤Ò检验	
	绿¦aAI	¤ôÊ^AI	
个®×数	42	42	
¥¿态参数a,b	¥­§¡­È	97.608474	87.721510	
	标­ã®t	1.4412057	18.4697541	
³ÌÌåºÝ®t­È	绝对	.148	.256	
	¥¿	.070	.256	
	负	-.148	-.213	
检验统计	.148	.256	
渐ªñ显µÛ©Ê¡]双§À¡^	.022c	.000c	

a. 检验¤À¥¬为¥¿态¤À¥¬¡C	
b. ®ÚÕu数Õu计ºâ¡C	
c. ¨½§Q¤ó显µÛ©Ê­×¥¿¡C	
d. 这¬O¯u显µÛ©Êªº¤U­­¡C	

NONPAR CORR
  /VARIABLES=¥­§¡¦aªí温«× ¤£³z¤ô­±PD
  /PRINT=SPEARMAN TWOTAIL NOSIG
  /MISSING=PAIRWISE.


«D参数¬Û关©Ê


备ª`	
¤w创«Ø输¥X	23-OCT-2023 12:41:33	
ª`释		
输¤J	数Õu	D:\ACCESSIBILITY AND PCI\SPSS\SPSS¥X图\无标题6.sav	
	¬¡动数Õu¶°	数Õu¶°1	
	过滤¾¹	<无>	
	权­«	<无>	
	©î¤À¤å¥ó	<无>	
	¤u§@数Õu¤å¥ó¤¤ªº¦æ数	45	
¯Ê¥¢­È处²z	对¯Ê¥¢ªº©w义	将¥Î户©w义ªº¯Ê¥¢­È视为¯Ê¥¢¡C	
	¨Ï¥Îªº个®×数	¨C对变¶qªº统计³£°ò¤_©Ò¦³对¤_该对变¶q¨ã¦³¦³®Ä数Õuªº个®×¡C	
语ªk	NONPAR CORR
  /VARIABLES=¥­§¡¦aªí温«× ¤£³z¤ô­±PD
  /PRINT=SPEARMAN TWOTAIL NOSIG
  /MISSING=PAIRWISE.	
资·½	处²zµ{§Ç时间	00:00:00.02	
	¯Ó¥Î时间	00:00:00.00	
	¤¹许ªº个®×数	629145 个个®×a	

a. °ò¤_¤u§@ªÅ间内¦sªº¥i¥Î©Ê	


¬Û关©Ê	
	¥­§¡¦aªí温«×	¤£³z¤ô­±PD	
´µ¥Ö尔°Ò Rho	¥­§¡¦aªí温«×	¬Û关¨t数	1.000	.727**	
		显µÛ©Ê¡]双§À¡^	.	.000	
		个®×数	42	42	
	¤£³z¤ô­±PD	¬Û关¨t数	.727**	1.000	
		显µÛ©Ê¡]双§À¡^	.000	.	
		个®×数	42	42	

**. ¦b 0.01 级别¡]双§À¡^¡A¬Û关©Ê显µÛ¡C	

NONPAR CORR
  /VARIABLES=¥­§¡¦aªí温«× 绿¦aPD
  /PRINT=SPEARMAN TWOTAIL NOSIG
  /MISSING=PAIRWISE.


«D参数¬Û关©Ê


备ª`	
¤w创«Ø输¥X	23-OCT-2023 12:42:18	
ª`释		
输¤J	数Õu	D:\ACCESSIBILITY AND PCI\SPSS\SPSS¥X图\无标题6.sav	
	¬¡动数Õu¶°	数Õu¶°1	
	过滤¾¹	<无>	
	权­«	<无>	
	©î¤À¤å¥ó	<无>	
	¤u§@数Õu¤å¥ó¤¤ªº¦æ数	45	
¯Ê¥¢­È处²z	对¯Ê¥¢ªº©w义	将¥Î户©w义ªº¯Ê¥¢­È视为¯Ê¥¢¡C	
	¨Ï¥Îªº个®×数	¨C对变¶qªº统计³£°ò¤_©Ò¦³对¤_该对变¶q¨ã¦³¦³®Ä数Õuªº个®×¡C	
语ªk	NONPAR CORR
  /VARIABLES=¥­§¡¦aªí温«× 绿¦aPD
  /PRINT=SPEARMAN TWOTAIL NOSIG
  /MISSING=PAIRWISE.	
资·½	处²zµ{§Ç时间	00:00:00.00	
	¯Ó¥Î时间	00:00:00.00	
	¤¹许ªº个®×数	629145 个个®×a	

a. °ò¤_¤u§@ªÅ间内¦sªº¥i¥Î©Ê	


¬Û关©Ê	
	¥­§¡¦aªí温«×	绿¦aPD	
´µ¥Ö尔°Ò Rho	¥­§¡¦aªí温«×	¬Û关¨t数	1.000	.653**	
		显µÛ©Ê¡]双§À¡^	.	.000	
		个®×数	42	42	
	绿¦aPD	¬Û关¨t数	.653**	1.000	
		显µÛ©Ê¡]双§À¡^	.000	.	
		个®×数	42	42	

**. ¦b 0.01 级别¡]双§À¡^¡A¬Û关©Ê显µÛ¡C	

NONPAR CORR
  /VARIABLES=¥­§¡¦aªí温«× ¤ôÊ^PD
  /PRINT=SPEARMAN TWOTAIL NOSIG
  /MISSING=PAIRWISE.


«D参数¬Û关©Ê


备ª`	
¤w创«Ø输¥X	23-OCT-2023 12:42:37	
ª`释		
输¤J	数Õu	D:\ACCESSIBILITY AND PCI\SPSS\SPSS¥X图\无标题6.sav	
	¬¡动数Õu¶°	数Õu¶°1	
	过滤¾¹	<无>	
	权­«	<无>	
	©î¤À¤å¥ó	<无>	
	¤u§@数Õu¤å¥ó¤¤ªº¦æ数	45	
¯Ê¥¢­È处²z	对¯Ê¥¢ªº©w义	将¥Î户©w义ªº¯Ê¥¢­È视为¯Ê¥¢¡C	
	¨Ï¥Îªº个®×数	¨C对变¶qªº统计³£°ò¤_©Ò¦³对¤_该对变¶q¨ã¦³¦³®Ä数Õuªº个®×¡C	
语ªk	NONPAR CORR
  /VARIABLES=¥­§¡¦aªí温«× ¤ôÊ^PD
  /PRINT=SPEARMAN TWOTAIL NOSIG
  /MISSING=PAIRWISE.	
资·½	处²zµ{§Ç时间	00:00:00.02	
	¯Ó¥Î时间	00:00:00.00	
	¤¹许ªº个®×数	629145 个个®×a	

a. °ò¤_¤u§@ªÅ间内¦sªº¥i¥Î©Ê	


¬Û关©Ê	
	¥­§¡¦aªí温«×	¤ôÊ^PD	
´µ¥Ö尔°Ò Rho	¥­§¡¦aªí温«×	¬Û关¨t数	1.000	.410**	
		显µÛ©Ê¡]双§À¡^	.	.007	
		个®×数	42	42	
	¤ôÊ^PD	¬Û关¨t数	.410**	1.000	
		显µÛ©Ê¡]双§À¡^	.007	.	
		个®×数	42	42	

**. ¦b 0.01 级别¡]双§À¡^¡A¬Û关©Ê显µÛ¡C	

NONPAR CORR
  /VARIABLES=¥­§¡¦aªí温«× ¤£³z¤ô­±LPI
  /PRINT=SPEARMAN TWOTAIL NOSIG
  /MISSING=PAIRWISE.


«D参数¬Û关©Ê


备ª`	
¤w创«Ø输¥X	23-OCT-2023 12:42:55	
ª`释		
输¤J	数Õu	D:\ACCESSIBILITY AND PCI\SPSS\SPSS¥X图\无标题6.sav	
	¬¡动数Õu¶°	数Õu¶°1	
	过滤¾¹	<无>	
	权­«	<无>	
	©î¤À¤å¥ó	<无>	
	¤u§@数Õu¤å¥ó¤¤ªº¦æ数	45	
¯Ê¥¢­È处²z	对¯Ê¥¢ªº©w义	将¥Î户©w义ªº¯Ê¥¢­È视为¯Ê¥¢¡C	
	¨Ï¥Îªº个®×数	¨C对变¶qªº统计³£°ò¤_©Ò¦³对¤_该对变¶q¨ã¦³¦³®Ä数Õuªº个®×¡C	
语ªk	NONPAR CORR
  /VARIABLES=¥­§¡¦aªí温«× ¤£³z¤ô­±LPI
  /PRINT=SPEARMAN TWOTAIL NOSIG
  /MISSING=PAIRWISE.	
资·½	处²zµ{§Ç时间	00:00:00.00	
	¯Ó¥Î时间	00:00:00.00	
	¤¹许ªº个®×数	629145 个个®×a	

a. °ò¤_¤u§@ªÅ间内¦sªº¥i¥Î©Ê	


¬Û关©Ê	
	¥­§¡¦aªí温«×	¤£³z¤ô­±LPI	
´µ¥Ö尔°Ò Rho	¥­§¡¦aªí温«×	¬Û关¨t数	1.000	.684**	
		显µÛ©Ê¡]双§À¡^	.	.000	
		个®×数	42	42	
	¤£³z¤ô­±LPI	¬Û关¨t数	.684**	1.000	
		显µÛ©Ê¡]双§À¡^	.000	.	
		个®×数	42	42	

**. ¦b 0.01 级别¡]双§À¡^¡A¬Û关©Ê显µÛ¡C	

NONPAR CORR
  /VARIABLES=¥­§¡¦aªí温«× 绿¦aLPI
  /PRINT=SPEARMAN TWOTAIL NOSIG
  /MISSING=PAIRWISE.


«D参数¬Û关©Ê


备ª`	
¤w创«Ø输¥X	23-OCT-2023 12:43:13	
ª`释		
输¤J	数Õu	D:\ACCESSIBILITY AND PCI\SPSS\SPSS¥X图\无标题6.sav	
	¬¡动数Õu¶°	数Õu¶°1	
	过滤¾¹	<无>	
	权­«	<无>	
	©î¤À¤å¥ó	<无>	
	¤u§@数Õu¤å¥ó¤¤ªº¦æ数	45	
¯Ê¥¢­È处²z	对¯Ê¥¢ªº©w义	将¥Î户©w义ªº¯Ê¥¢­È视为¯Ê¥¢¡C	
	¨Ï¥Îªº个®×数	¨C对变¶qªº统计³£°ò¤_©Ò¦³对¤_该对变¶q¨ã¦³¦³®Ä数Õuªº个®×¡C	
语ªk	NONPAR CORR
  /VARIABLES=¥­§¡¦aªí温«× 绿¦aLPI
  /PRINT=SPEARMAN TWOTAIL NOSIG
  /MISSING=PAIRWISE.	
资·½	处²zµ{§Ç时间	00:00:00.00	
	¯Ó¥Î时间	00:00:00.00	
	¤¹许ªº个®×数	629145 个个®×a	

a. °ò¤_¤u§@ªÅ间内¦sªº¥i¥Î©Ê	


¬Û关©Ê	
	¥­§¡¦aªí温«×	绿¦aLPI	
´µ¥Ö尔°Ò Rho	¥­§¡¦aªí温«×	¬Û关¨t数	1.000	.166	
		显µÛ©Ê¡]双§À¡^	.	.293	
		个®×数	42	42	
	绿¦aLPI	¬Û关¨t数	.166	1.000	
		显µÛ©Ê¡]双§À¡^	.293	.	
		个®×数	42	42	

NONPAR CORR
  /VARIABLES=¥­§¡¦aªí温«× ¤ôÊ^LPI
  /PRINT=SPEARMAN TWOTAIL NOSIG
  /MISSING=PAIRWISE.


«D参数¬Û关©Ê


备ª`	
¤w创«Ø输¥X	23-OCT-2023 12:43:31	
ª`释		
输¤J	数Õu	D:\ACCESSIBILITY AND PCI\SPSS\SPSS¥X图\无标题6.sav	
	¬¡动数Õu¶°	数Õu¶°1	
	过滤¾¹	<无>	
	权­«	<无>	
	©î¤À¤å¥ó	<无>	
	¤u§@数Õu¤å¥ó¤¤ªº¦æ数	45	
¯Ê¥¢­È处²z	对¯Ê¥¢ªº©w义	将¥Î户©w义ªº¯Ê¥¢­È视为¯Ê¥¢¡C	
	¨Ï¥Îªº个®×数	¨C对变¶qªº统计³£°ò¤_©Ò¦³对¤_该对变¶q¨ã¦³¦³®Ä数Õuªº个®×¡C	
语ªk	NONPAR CORR
  /VARIABLES=¥­§¡¦aªí温«× ¤ôÊ^LPI
  /PRINT=SPEARMAN TWOTAIL NOSIG
  /MISSING=PAIRWISE.	
资·½	处²zµ{§Ç时间	00:00:00.00	
	¯Ó¥Î时间	00:00:00.00	
	¤¹许ªº个®×数	629145 个个®×a	

a. °ò¤_¤u§@ªÅ间内¦sªº¥i¥Î©Ê	


¬Û关©Ê	
	¥­§¡¦aªí温«×	¤ôÊ^LPI	
´µ¥Ö尔°Ò Rho	¥­§¡¦aªí温«×	¬Û关¨t数	1.000	-.444**	
		显µÛ©Ê¡]双§À¡^	.	.003	
		个®×数	42	42	
	¤ôÊ^LPI	¬Û关¨t数	-.444**	1.000	
		显µÛ©Ê¡]双§À¡^	.003	.	
		个®×数	42	42	

**. ¦b 0.01 级别¡]双§À¡^¡A¬Û关©Ê显µÛ¡C	

NONPAR CORR
  /VARIABLES=¥­§¡¦aªí温«× ¤£³z¤ô­±LSI
  /PRINT=SPEARMAN TWOTAIL NOSIG
  /MISSING=PAIRWISE.


«D参数¬Û关©Ê


备ª`	
¤w创«Ø输¥X	23-OCT-2023 12:43:55	
ª`释		
输¤J	数Õu	D:\ACCESSIBILITY AND PCI\SPSS\SPSS¥X图\无标题6.sav	
	¬¡动数Õu¶°	数Õu¶°1	
	过滤¾¹	<无>	
	权­«	<无>	
	©î¤À¤å¥ó	<无>	
	¤u§@数Õu¤å¥ó¤¤ªº¦æ数	45	
¯Ê¥¢­È处²z	对¯Ê¥¢ªº©w义	将¥Î户©w义ªº¯Ê¥¢­È视为¯Ê¥¢¡C	
	¨Ï¥Îªº个®×数	¨C对变¶qªº统计³£°ò¤_©Ò¦³对¤_该对变¶q¨ã¦³¦³®Ä数Õuªº个®×¡C	
语ªk	NONPAR CORR
  /VARIABLES=¥­§¡¦aªí温«× ¤£³z¤ô­±LSI
  /PRINT=SPEARMAN TWOTAIL NOSIG
  /MISSING=PAIRWISE.	
资·½	处²zµ{§Ç时间	00:00:00.00	
	¯Ó¥Î时间	00:00:00.00	
	¤¹许ªº个®×数	629145 个个®×a	

a. °ò¤_¤u§@ªÅ间内¦sªº¥i¥Î©Ê	


¬Û关©Ê	
	¥­§¡¦aªí温«×	¤£³z¤ô­±LSI	
´µ¥Ö尔°Ò Rho	¥­§¡¦aªí温«×	¬Û关¨t数	1.000	-.236	
		显µÛ©Ê¡]双§À¡^	.	.133	
		个®×数	42	42	
	¤£³z¤ô­±LSI	¬Û关¨t数	-.236	1.000	
		显µÛ©Ê¡]双§À¡^	.133	.	
		个®×数	42	42	

NONPAR CORR
  /VARIABLES=¥­§¡¦aªí温«× 绿¦aLSI
  /PRINT=SPEARMAN TWOTAIL NOSIG
  /MISSING=PAIRWISE.


«D参数¬Û关©Ê


备ª`	
¤w创«Ø输¥X	23-OCT-2023 12:44:19	
ª`释		
输¤J	数Õu	D:\ACCESSIBILITY AND PCI\SPSS\SPSS¥X图\无标题6.sav	
	¬¡动数Õu¶°	数Õu¶°1	
	过滤¾¹	<无>	
	权­«	<无>	
	©î¤À¤å¥ó	<无>	
	¤u§@数Õu¤å¥ó¤¤ªº¦æ数	45	
¯Ê¥¢­È处²z	对¯Ê¥¢ªº©w义	将¥Î户©w义ªº¯Ê¥¢­È视为¯Ê¥¢¡C	
	¨Ï¥Îªº个®×数	¨C对变¶qªº统计³£°ò¤_©Ò¦³对¤_该对变¶q¨ã¦³¦³®Ä数Õuªº个®×¡C	
语ªk	NONPAR CORR
  /VARIABLES=¥­§¡¦aªí温«× 绿¦aLSI
  /PRINT=SPEARMAN TWOTAIL NOSIG
  /MISSING=PAIRWISE.	
资·½	处²zµ{§Ç时间	00:00:00.00	
	¯Ó¥Î时间	00:00:00.01	
	¤¹许ªº个®×数	629145 个个®×a	

a. °ò¤_¤u§@ªÅ间内¦sªº¥i¥Î©Ê	


¬Û关©Ê	
	¥­§¡¦aªí温«×	绿¦aLSI	
´µ¥Ö尔°Ò Rho	¥­§¡¦aªí温«×	¬Û关¨t数	1.000	-.161	
		显µÛ©Ê¡]双§À¡^	.	.308	
		个®×数	42	42	
	绿¦aLSI	¬Û关¨t数	-.161	1.000	
		显µÛ©Ê¡]双§À¡^	.308	.	
		个®×数	42	42	

NONPAR CORR
  /VARIABLES=¥­§¡¦aªí温«× ¤ôÊ^LSI
  /PRINT=SPEARMAN TWOTAIL NOSIG
  /MISSING=PAIRWISE.


«D参数¬Û关©Ê


备ª`	
¤w创«Ø输¥X	23-OCT-2023 12:44:39	
ª`释		
输¤J	数Õu	D:\ACCESSIBILITY AND PCI\SPSS\SPSS¥X图\无标题6.sav	
	¬¡动数Õu¶°	数Õu¶°1	
	过滤¾¹	<无>	
	权­«	<无>	
	©î¤À¤å¥ó	<无>	
	¤u§@数Õu¤å¥ó¤¤ªº¦æ数	45	
¯Ê¥¢­È处²z	对¯Ê¥¢ªº©w义	将¥Î户©w义ªº¯Ê¥¢­È视为¯Ê¥¢¡C	
	¨Ï¥Îªº个®×数	¨C对变¶qªº统计³£°ò¤_©Ò¦³对¤_该对变¶q¨ã¦³¦³®Ä数Õuªº个®×¡C	
语ªk	NONPAR CORR
  /VARIABLES=¥­§¡¦aªí温«× ¤ôÊ^LSI
  /PRINT=SPEARMAN TWOTAIL NOSIG
  /MISSING=PAIRWISE.	
资·½	处²zµ{§Ç时间	00:00:00.02	
	¯Ó¥Î时间	00:00:00.00	
	¤¹许ªº个®×数	629145 个个®×a	

a. °ò¤_¤u§@ªÅ间内¦sªº¥i¥Î©Ê	


¬Û关©Ê	
	¥­§¡¦aªí温«×	¤ôÊ^LSI	
´µ¥Ö尔°Ò Rho	¥­§¡¦aªí温«×	¬Û关¨t数	1.000	-.096	
		显µÛ©Ê¡]双§À¡^	.	.545	
		个®×数	42	42	
	¤ôÊ^LSI	¬Û关¨t数	-.096	1.000	
		显µÛ©Ê¡]双§À¡^	.545	.	
		个®×数	42	42	

NONPAR CORR
  /VARIABLES=¥­§¡¦aªí温«× ¤£³z¤ô­±DIVISION
  /PRINT=SPEARMAN TWOTAIL NOSIG
  /MISSING=PAIRWISE.


«D参数¬Û关©Ê


备ª`	
¤w创«Ø输¥X	23-OCT-2023 12:44:57	
ª`释		
输¤J	数Õu	D:\ACCESSIBILITY AND PCI\SPSS\SPSS¥X图\无标题6.sav	
	¬¡动数Õu¶°	数Õu¶°1	
	过滤¾¹	<无>	
	权­«	<无>	
	©î¤À¤å¥ó	<无>	
	¤u§@数Õu¤å¥ó¤¤ªº¦æ数	45	
¯Ê¥¢­È处²z	对¯Ê¥¢ªº©w义	将¥Î户©w义ªº¯Ê¥¢­È视为¯Ê¥¢¡C	
	¨Ï¥Îªº个®×数	¨C对变¶qªº统计³£°ò¤_©Ò¦³对¤_该对变¶q¨ã¦³¦³®Ä数Õuªº个®×¡C	
语ªk	NONPAR CORR
  /VARIABLES=¥­§¡¦aªí温«× ¤£³z¤ô­±DIVISION
  /PRINT=SPEARMAN TWOTAIL NOSIG
  /MISSING=PAIRWISE.	
资·½	处²zµ{§Ç时间	00:00:00.00	
	¯Ó¥Î时间	00:00:00.00	
	¤¹许ªº个®×数	629145 个个®×a	

a. °ò¤_¤u§@ªÅ间内¦sªº¥i¥Î©Ê	


¬Û关©Ê	
	¥­§¡¦aªí温«×	¤£³z¤ô­±DIVISION	
´µ¥Ö尔°Ò Rho	¥­§¡¦aªí温«×	¬Û关¨t数	1.000	-.703**	
		显µÛ©Ê¡]双§À¡^	.	.000	
		个®×数	42	42	
	¤£³z¤ô­±DIVISION	¬Û关¨t数	-.703**	1.000	
		显µÛ©Ê¡]双§À¡^	.000	.	
		个®×数	42	42	

**. ¦b 0.01 级别¡]双§À¡^¡A¬Û关©Ê显µÛ¡C	

NONPAR CORR
  /VARIABLES=¥­§¡¦aªí温«× 绿¦aDIVISION
  /PRINT=SPEARMAN TWOTAIL NOSIG
  /MISSING=PAIRWISE.


«D参数¬Û关©Ê


备ª`	
¤w创«Ø输¥X	23-OCT-2023 12:45:22	
ª`释		
输¤J	数Õu	D:\ACCESSIBILITY AND PCI\SPSS\SPSS¥X图\无标题6.sav	
	¬¡动数Õu¶°	数Õu¶°1	
	过滤¾¹	<无>	
	权­«	<无>	
	©î¤À¤å¥ó	<无>	
	¤u§@数Õu¤å¥ó¤¤ªº¦æ数	45	
¯Ê¥¢­È处²z	对¯Ê¥¢ªº©w义	将¥Î户©w义ªº¯Ê¥¢­È视为¯Ê¥¢¡C	
	¨Ï¥Îªº个®×数	¨C对变¶qªº统计³£°ò¤_©Ò¦³对¤_该对变¶q¨ã¦³¦³®Ä数Õuªº个®×¡C	
语ªk	NONPAR CORR
  /VARIABLES=¥­§¡¦aªí温«× 绿¦aDIVISION
  /PRINT=SPEARMAN TWOTAIL NOSIG
  /MISSING=PAIRWISE.	
资·½	处²zµ{§Ç时间	00:00:00.00	
	¯Ó¥Î时间	00:00:00.00	
	¤¹许ªº个®×数	629145 个个®×a	

a. °ò¤_¤u§@ªÅ间内¦sªº¥i¥Î©Ê	


¬Û关©Ê	
	¥­§¡¦aªí温«×	绿¦aDIVISION	
´µ¥Ö尔°Ò Rho	¥­§¡¦aªí温«×	¬Û关¨t数	1.000	-.208	
		显µÛ©Ê¡]双§À¡^	.	.186	
		个®×数	42	42	
	绿¦aDIVISION	¬Û关¨t数	-.208	1.000	
		显µÛ©Ê¡]双§À¡^	.186	.	
		个®×数	42	42	

NONPAR CORR
  /VARIABLES=¥­§¡¦aªí温«× ¤ôÊ^DIVISION
  /PRINT=SPEARMAN TWOTAIL NOSIG
  /MISSING=PAIRWISE.


«D参数¬Û关©Ê


备ª`	
¤w创«Ø输¥X	23-OCT-2023 12:45:38	
ª`释		
输¤J	数Õu	D:\ACCESSIBILITY AND PCI\SPSS\SPSS¥X图\无标题6.sav	
	¬¡动数Õu¶°	数Õu¶°1	
	过滤¾¹	<无>	
	权­«	<无>	
	©î¤À¤å¥ó	<无>	
	¤u§@数Õu¤å¥ó¤¤ªº¦æ数	45	
¯Ê¥¢­È处²z	对¯Ê¥¢ªº©w义	将¥Î户©w义ªº¯Ê¥¢­È视为¯Ê¥¢¡C	
	¨Ï¥Îªº个®×数	¨C对变¶qªº统计³£°ò¤_©Ò¦³对¤_该对变¶q¨ã¦³¦³®Ä数Õuªº个®×¡C	
语ªk	NONPAR CORR
  /VARIABLES=¥­§¡¦aªí温«× ¤ôÊ^DIVISION
  /PRINT=SPEARMAN TWOTAIL NOSIG
  /MISSING=PAIRWISE.	
资·½	处²zµ{§Ç时间	00:00:00.02	
	¯Ó¥Î时间	00:00:00.01	
	¤¹许ªº个®×数	629145 个个®×a	

a. °ò¤_¤u§@ªÅ间内¦sªº¥i¥Î©Ê	


¬Û关©Ê	
	¥­§¡¦aªí温«×	¤ôÊ^DIVISION	
´µ¥Ö尔°Ò Rho	¥­§¡¦aªí温«×	¬Û关¨t数	1.000	.443**	
		显µÛ©Ê¡]双§À¡^	.	.003	
		个®×数	42	42	
	¤ôÊ^DIVISION	¬Û关¨t数	.443**	1.000	
		显µÛ©Ê¡]双§À¡^	.003	.	
		个®×数	42	42	

**. ¦b 0.01 级别¡]双§À¡^¡A¬Û关©Ê显µÛ¡C	

NONPAR CORR
  /VARIABLES=¥­§¡¦aªí温«× ¤£³z¤ô­±SPLIT
  /PRINT=SPEARMAN TWOTAIL NOSIG
  /MISSING=PAIRWISE.


«D参数¬Û关©Ê


备ª`	
¤w创«Ø输¥X	23-OCT-2023 12:45:57	
ª`释		
输¤J	数Õu	D:\ACCESSIBILITY AND PCI\SPSS\SPSS¥X图\无标题6.sav	
	¬¡动数Õu¶°	数Õu¶°1	
	过滤¾¹	<无>	
	权­«	<无>	
	©î¤À¤å¥ó	<无>	
	¤u§@数Õu¤å¥ó¤¤ªº¦æ数	45	
¯Ê¥¢­È处²z	对¯Ê¥¢ªº©w义	将¥Î户©w义ªº¯Ê¥¢­È视为¯Ê¥¢¡C	
	¨Ï¥Îªº个®×数	¨C对变¶qªº统计³£°ò¤_©Ò¦³对¤_该对变¶q¨ã¦³¦³®Ä数Õuªº个®×¡C	
语ªk	NONPAR CORR
  /VARIABLES=¥­§¡¦aªí温«× ¤£³z¤ô­±SPLIT
  /PRINT=SPEARMAN TWOTAIL NOSIG
  /MISSING=PAIRWISE.	
资·½	处²zµ{§Ç时间	00:00:00.00	
	¯Ó¥Î时间	00:00:00.00	
	¤¹许ªº个®×数	629145 个个®×a	

a. °ò¤_¤u§@ªÅ间内¦sªº¥i¥Î©Ê	


¬Û关©Ê	
	¥­§¡¦aªí温«×	¤£³z¤ô­±SPLIT	
´µ¥Ö尔°Ò Rho	¥­§¡¦aªí温«×	¬Û关¨t数	1.000	-.698**	
		显µÛ©Ê¡]双§À¡^	.	.000	
		个®×数	42	42	
	¤£³z¤ô­±SPLIT	¬Û关¨t数	-.698**	1.000	
		显µÛ©Ê¡]双§À¡^	.000	.	
		个®×数	42	42	

**. ¦b 0.01 级别¡]双§À¡^¡A¬Û关©Ê显µÛ¡C	

NONPAR CORR
  /VARIABLES=¥­§¡¦aªí温«× 绿¦aSPLIT
  /PRINT=SPEARMAN TWOTAIL NOSIG
  /MISSING=PAIRWISE.


«D参数¬Û关©Ê


备ª`	
¤w创«Ø输¥X	23-OCT-2023 12:46:22	
ª`释		
输¤J	数Õu	D:\ACCESSIBILITY AND PCI\SPSS\SPSS¥X图\无标题6.sav	
	¬¡动数Õu¶°	数Õu¶°1	
	过滤¾¹	<无>	
	权­«	<无>	
	©î¤À¤å¥ó	<无>	
	¤u§@数Õu¤å¥ó¤¤ªº¦æ数	45	
¯Ê¥¢­È处²z	对¯Ê¥¢ªº©w义	将¥Î户©w义ªº¯Ê¥¢­È视为¯Ê¥¢¡C	
	¨Ï¥Îªº个®×数	¨C对变¶qªº统计³£°ò¤_©Ò¦³对¤_该对变¶q¨ã¦³¦³®Ä数Õuªº个®×¡C	
语ªk	NONPAR CORR
  /VARIABLES=¥­§¡¦aªí温«× 绿¦aSPLIT
  /PRINT=SPEARMAN TWOTAIL NOSIG
  /MISSING=PAIRWISE.	
资·½	处²zµ{§Ç时间	00:00:00.00	
	¯Ó¥Î时间	00:00:00.00	
	¤¹许ªº个®×数	629145 个个®×a	

a. °ò¤_¤u§@ªÅ间内¦sªº¥i¥Î©Ê	


¬Û关©Ê	
	¥­§¡¦aªí温«×	绿¦aSPLIT	
´µ¥Ö尔°Ò Rho	¥­§¡¦aªí温«×	¬Û关¨t数	1.000	-.207	
		显µÛ©Ê¡]双§À¡^	.	.188	
		个®×数	42	42	
	绿¦aSPLIT	¬Û关¨t数	-.207	1.000	
		显µÛ©Ê¡]双§À¡^	.188	.	
		个®×数	42	42	

NONPAR CORR
  /VARIABLES=¥­§¡¦aªí温«× ¤ôÊ^SPLIT
  /PRINT=SPEARMAN TWOTAIL NOSIG
  /MISSING=PAIRWISE.


«D参数¬Û关©Ê


备ª`	
¤w创«Ø输¥X	23-OCT-2023 12:46:38	
ª`释		
输¤J	数Õu	D:\ACCESSIBILITY AND PCI\SPSS\SPSS¥X图\无标题6.sav	
	¬¡动数Õu¶°	数Õu¶°1	
	过滤¾¹	<无>	
	权­«	<无>	
	©î¤À¤å¥ó	<无>	
	¤u§@数Õu¤å¥ó¤¤ªº¦æ数	45	
¯Ê¥¢­È处²z	对¯Ê¥¢ªº©w义	将¥Î户©w义ªº¯Ê¥¢­È视为¯Ê¥¢¡C	
	¨Ï¥Îªº个®×数	¨C对变¶qªº统计³£°ò¤_©Ò¦³对¤_该对变¶q¨ã¦³¦³®Ä数Õuªº个®×¡C	
语ªk	NONPAR CORR
  /VARIABLES=¥­§¡¦aªí温«× ¤ôÊ^SPLIT
  /PRINT=SPEARMAN TWOTAIL NOSIG
  /MISSING=PAIRWISE.	
资·½	处²zµ{§Ç时间	00:00:00.00	
	¯Ó¥Î时间	00:00:00.00	
	¤¹许ªº个®×数	629145 个个®×a	

a. °ò¤_¤u§@ªÅ间内¦sªº¥i¥Î©Ê	


¬Û关©Ê	
	¥­§¡¦aªí温«×	¤ôÊ^SPLIT	
´µ¥Ö尔°Ò Rho	¥­§¡¦aªí温«×	¬Û关¨t数	1.000	.451**	
		显µÛ©Ê¡]双§À¡^	.	.003	
		个®×数	42	42	
	¤ôÊ^SPLIT	¬Û关¨t数	.451**	1.000	
		显µÛ©Ê¡]双§À¡^	.003	.	
		个®×数	42	42	

**. ¦b 0.01 级别¡]双§À¡^¡A¬Û关©Ê显µÛ¡C	

NONPAR CORR
  /VARIABLES=¥­§¡¦aªí温«× ¤£³z¤ô­±AI
  /PRINT=SPEARMAN TWOTAIL NOSIG
  /MISSING=PAIRWISE.


«D参数¬Û关©Ê


备ª`	
¤w创«Ø输¥X	23-OCT-2023 12:47:00	
ª`释		
输¤J	数Õu	D:\ACCESSIBILITY AND PCI\SPSS\SPSS¥X图\无标题6.sav	
	¬¡动数Õu¶°	数Õu¶°1	
	过滤¾¹	<无>	
	权­«	<无>	
	©î¤À¤å¥ó	<无>	
	¤u§@数Õu¤å¥ó¤¤ªº¦æ数	45	
¯Ê¥¢­È处²z	对¯Ê¥¢ªº©w义	将¥Î户©w义ªº¯Ê¥¢­È视为¯Ê¥¢¡C	
	¨Ï¥Îªº个®×数	¨C对变¶qªº统计³£°ò¤_©Ò¦³对¤_该对变¶q¨ã¦³¦³®Ä数Õuªº个®×¡C	
语ªk	NONPAR CORR
  /VARIABLES=¥­§¡¦aªí温«× ¤£³z¤ô­±AI
  /PRINT=SPEARMAN TWOTAIL NOSIG
  /MISSING=PAIRWISE.	
资·½	处²zµ{§Ç时间	00:00:00.02	
	¯Ó¥Î时间	00:00:00.00	
	¤¹许ªº个®×数	629145 个个®×a	

a. °ò¤_¤u§@ªÅ间内¦sªº¥i¥Î©Ê	


¬Û关©Ê	
	¥­§¡¦aªí温«×	¤£³z¤ô­±AI	
´µ¥Ö尔°Ò Rho	¥­§¡¦aªí温«×	¬Û关¨t数	1.000	.164	
		显µÛ©Ê¡]双§À¡^	.	.298	
		个®×数	42	42	
	¤£³z¤ô­±AI	¬Û关¨t数	.164	1.000	
		显µÛ©Ê¡]双§À¡^	.298	.	
		个®×数	42	42	

NONPAR CORR
  /VARIABLES=¥­§¡¦aªí温«× 绿¦aAI
  /PRINT=SPEARMAN TWOTAIL NOSIG
  /MISSING=PAIRWISE.


«D参数¬Û关©Ê


备ª`	
¤w创«Ø输¥X	23-OCT-2023 12:47:14	
ª`释		
输¤J	数Õu	D:\ACCESSIBILITY AND PCI\SPSS\SPSS¥X图\无标题6.sav	
	¬¡动数Õu¶°	数Õu¶°1	
	过滤¾¹	<无>	
	权­«	<无>	
	©î¤À¤å¥ó	<无>	
	¤u§@数Õu¤å¥ó¤¤ªº¦æ数	45	
¯Ê¥¢­È处²z	对¯Ê¥¢ªº©w义	将¥Î户©w义ªº¯Ê¥¢­È视为¯Ê¥¢¡C	
	¨Ï¥Îªº个®×数	¨C对变¶qªº统计³£°ò¤_©Ò¦³对¤_该对变¶q¨ã¦³¦³®Ä数Õuªº个®×¡C	
语ªk	NONPAR CORR
  /VARIABLES=¥­§¡¦aªí温«× 绿¦aAI
  /PRINT=SPEARMAN TWOTAIL NOSIG
  /MISSING=PAIRWISE.	
资·½	处²zµ{§Ç时间	00:00:00.00	
	¯Ó¥Î时间	00:00:00.00	
	¤¹许ªº个®×数	629145 个个®×a	

a. °ò¤_¤u§@ªÅ间内¦sªº¥i¥Î©Ê	


¬Û关©Ê	
	¥­§¡¦aªí温«×	绿¦aAI	
´µ¥Ö尔°Ò Rho	¥­§¡¦aªí温«×	¬Û关¨t数	1.000	-.376*	
		显µÛ©Ê¡]双§À¡^	.	.014	
		个®×数	42	42	
	绿¦aAI	¬Û关¨t数	-.376*	1.000	
		显µÛ©Ê¡]双§À¡^	.014	.	
		个®×数	42	42	

*. ¦b 0.05 级别¡]双§À¡^¡A¬Û关©Ê显µÛ¡C	

NONPAR CORR
  /VARIABLES=¥­§¡¦aªí温«× ¤ôÊ^AI
  /PRINT=SPEARMAN TWOTAIL NOSIG
  /MISSING=PAIRWISE.


«D参数¬Û关©Ê


备ª`	
¤w创«Ø输¥X	23-OCT-2023 12:47:41	
ª`释		
输¤J	数Õu	D:\ACCESSIBILITY AND PCI\SPSS\SPSS¥X图\无标题6.sav	
	¬¡动数Õu¶°	数Õu¶°1	
	过滤¾¹	<无>	
	权­«	<无>	
	©î¤À¤å¥ó	<无>	
	¤u§@数Õu¤å¥ó¤¤ªº¦æ数	45	
¯Ê¥¢­È处²z	对¯Ê¥¢ªº©w义	将¥Î户©w义ªº¯Ê¥¢­È视为¯Ê¥¢¡C	
	¨Ï¥Îªº个®×数	¨C对变¶qªº统计³£°ò¤_©Ò¦³对¤_该对变¶q¨ã¦³¦³®Ä数Õuªº个®×¡C	
语ªk	NONPAR CORR
  /VARIABLES=¥­§¡¦aªí温«× ¤ôÊ^AI
  /PRINT=SPEARMAN TWOTAIL NOSIG
  /MISSING=PAIRWISE.	
资·½	处²zµ{§Ç时间	00:00:00.02	
	¯Ó¥Î时间	00:00:00.00	
	¤¹许ªº个®×数	629145 个个®×a	

a. °ò¤_¤u§@ªÅ间内¦sªº¥i¥Î©Ê	


¬Û关©Ê	
	¥­§¡¦aªí温«×	¤ôÊ^AI	
´µ¥Ö尔°Ò Rho	¥­§¡¦aªí温«×	¬Û关¨t数	1.000	-.653**	
		显µÛ©Ê¡]双§À¡^	.	.000	
		个®×数	42	42	
	¤ôÊ^AI	¬Û关¨t数	-.653**	1.000	
		显µÛ©Ê¡]双§À¡^	.000	.	
		个®×数	42	42	

**. ¦b 0.01 级别¡]双§À¡^¡A¬Û关©Ê显µÛ¡C	

NONPAR CORR
  /VARIABLES=­°温­S围 ¤£³z¤ô­±PD
  /PRINT=SPEARMAN TWOTAIL NOSIG
  /MISSING=PAIRWISE.


«D参数¬Û关©Ê


备ª`	
¤w创«Ø输¥X	23-OCT-2023 21:12:00	
ª`释		
输¤J	数Õu	D:\ACCESSIBILITY AND PCI\SPSS\SPSS¥X图\无标题6.sav	
	¬¡动数Õu¶°	数Õu¶°1	
	过滤¾¹	<无>	
	权­«	<无>	
	©î¤À¤å¥ó	<无>	
	¤u§@数Õu¤å¥ó¤¤ªº¦æ数	45	
¯Ê¥¢­È处²z	对¯Ê¥¢ªº©w义	将¥Î户©w义ªº¯Ê¥¢­È视为¯Ê¥¢¡C	
	¨Ï¥Îªº个®×数	¨C对变¶qªº统计³£°ò¤_©Ò¦³对¤_该对变¶q¨ã¦³¦³®Ä数Õuªº个®×¡C	
语ªk	NONPAR CORR
  /VARIABLES=­°温­S围 ¤£³z¤ô­±PD
  /PRINT=SPEARMAN TWOTAIL NOSIG
  /MISSING=PAIRWISE.	
资·½	处²zµ{§Ç时间	00:00:00.00	
	¯Ó¥Î时间	00:00:00.01	
	¤¹许ªº个®×数	629145 个个®×a	

a. °ò¤_¤u§@ªÅ间内¦sªº¥i¥Î©Ê	


¬Û关©Ê	
	­°温­S围	¤£³z¤ô­±PD	
´µ¥Ö尔°Ò Rho	­°温­S围	¬Û关¨t数	1.000	-.108	
		显µÛ©Ê¡]双§À¡^	.	.497	
		个®×数	42	42	
	¤£³z¤ô­±PD	¬Û关¨t数	-.108	1.000	
		显µÛ©Ê¡]双§À¡^	.497	.	
		个®×数	42	42	

NONPAR CORR
  /VARIABLES=­°温­S围 绿¦aPD
  /PRINT=SPEARMAN TWOTAIL NOSIG
  /MISSING=PAIRWISE.


«D参数¬Û关©Ê


备ª`	
¤w创«Ø输¥X	23-OCT-2023 21:12:29	
ª`释		
输¤J	数Õu	D:\ACCESSIBILITY AND PCI\SPSS\SPSS¥X图\无标题6.sav	
	¬¡动数Õu¶°	数Õu¶°1	
	过滤¾¹	<无>	
	权­«	<无>	
	©î¤À¤å¥ó	<无>	
	¤u§@数Õu¤å¥ó¤¤ªº¦æ数	45	
¯Ê¥¢­È处²z	对¯Ê¥¢ªº©w义	将¥Î户©w义ªº¯Ê¥¢­È视为¯Ê¥¢¡C	
	¨Ï¥Îªº个®×数	¨C对变¶qªº统计³£°ò¤_©Ò¦³对¤_该对变¶q¨ã¦³¦³®Ä数Õuªº个®×¡C	
语ªk	NONPAR CORR
  /VARIABLES=­°温­S围 绿¦aPD
  /PRINT=SPEARMAN TWOTAIL NOSIG
  /MISSING=PAIRWISE.	
资·½	处²zµ{§Ç时间	00:00:00.00	
	¯Ó¥Î时间	00:00:00.00	
	¤¹许ªº个®×数	629145 个个®×a	

a. °ò¤_¤u§@ªÅ间内¦sªº¥i¥Î©Ê	


¬Û关©Ê	
	­°温­S围	绿¦aPD	
´µ¥Ö尔°Ò Rho	­°温­S围	¬Û关¨t数	1.000	-.048	
		显µÛ©Ê¡]双§À¡^	.	.763	
		个®×数	42	42	
	绿¦aPD	¬Û关¨t数	-.048	1.000	
		显µÛ©Ê¡]双§À¡^	.763	.	
		个®×数	42	42	

NONPAR CORR
  /VARIABLES=­°温­S围 ¤ôÊ^PD
  /PRINT=SPEARMAN TWOTAIL NOSIG
  /MISSING=PAIRWISE.


«D参数¬Û关©Ê


备ª`	
¤w创«Ø输¥X	23-OCT-2023 21:12:41	
ª`释		
输¤J	数Õu	D:\ACCESSIBILITY AND PCI\SPSS\SPSS¥X图\无标题6.sav	
	¬¡动数Õu¶°	数Õu¶°1	
	过滤¾¹	<无>	
	权­«	<无>	
	©î¤À¤å¥ó	<无>	
	¤u§@数Õu¤å¥ó¤¤ªº¦æ数	45	
¯Ê¥¢­È处²z	对¯Ê¥¢ªº©w义	将¥Î户©w义ªº¯Ê¥¢­È视为¯Ê¥¢¡C	
	¨Ï¥Îªº个®×数	¨C对变¶qªº统计³£°ò¤_©Ò¦³对¤_该对变¶q¨ã¦³¦³®Ä数Õuªº个®×¡C	
语ªk	NONPAR CORR
  /VARIABLES=­°温­S围 ¤ôÊ^PD
  /PRINT=SPEARMAN TWOTAIL NOSIG
  /MISSING=PAIRWISE.	
资·½	处²zµ{§Ç时间	00:00:00.00	
	¯Ó¥Î时间	00:00:00.00	
	¤¹许ªº个®×数	629145 个个®×a	

a. °ò¤_¤u§@ªÅ间内¦sªº¥i¥Î©Ê	


¬Û关©Ê	
	­°温­S围	¤ôÊ^PD	
´µ¥Ö尔°Ò Rho	­°温­S围	¬Û关¨t数	1.000	.013	
		显µÛ©Ê¡]双§À¡^	.	.934	
		个®×数	42	42	
	¤ôÊ^PD	¬Û关¨t数	.013	1.000	
		显µÛ©Ê¡]双§À¡^	.934	.	
		个®×数	42	42	

NONPAR CORR
  /VARIABLES=­°温­S围 ¤£³z¤ô­±LPI
  /PRINT=SPEARMAN TWOTAIL NOSIG
  /MISSING=PAIRWISE.


«D参数¬Û关©Ê


备ª`	
¤w创«Ø输¥X	23-OCT-2023 21:12:52	
ª`释		
输¤J	数Õu	D:\ACCESSIBILITY AND PCI\SPSS\SPSS¥X图\无标题6.sav	
	¬¡动数Õu¶°	数Õu¶°1	
	过滤¾¹	<无>	
	权­«	<无>	
	©î¤À¤å¥ó	<无>	
	¤u§@数Õu¤å¥ó¤¤ªº¦æ数	45	
¯Ê¥¢­È处²z	对¯Ê¥¢ªº©w义	将¥Î户©w义ªº¯Ê¥¢­È视为¯Ê¥¢¡C	
	¨Ï¥Îªº个®×数	¨C对变¶qªº统计³£°ò¤_©Ò¦³对¤_该对变¶q¨ã¦³¦³®Ä数Õuªº个®×¡C	
语ªk	NONPAR CORR
  /VARIABLES=­°温­S围 ¤£³z¤ô­±LPI
  /PRINT=SPEARMAN TWOTAIL NOSIG
  /MISSING=PAIRWISE.	
资·½	处²zµ{§Ç时间	00:00:00.00	
	¯Ó¥Î时间	00:00:00.00	
	¤¹许ªº个®×数	629145 个个®×a	

a. °ò¤_¤u§@ªÅ间内¦sªº¥i¥Î©Ê	


¬Û关©Ê	
	­°温­S围	¤£³z¤ô­±LPI	
´µ¥Ö尔°Ò Rho	­°温­S围	¬Û关¨t数	1.000	-.169	
		显µÛ©Ê¡]双§À¡^	.	.285	
		个®×数	42	42	
	¤£³z¤ô­±LPI	¬Û关¨t数	-.169	1.000	
		显µÛ©Ê¡]双§À¡^	.285	.	
		个®×数	42	42	

NONPAR CORR
  /VARIABLES=­°温­S围 绿¦aLPI
  /PRINT=SPEARMAN TWOTAIL NOSIG
  /MISSING=PAIRWISE.


«D参数¬Û关©Ê


备ª`	
¤w创«Ø输¥X	23-OCT-2023 21:13:03	
ª`释		
输¤J	数Õu	D:\ACCESSIBILITY AND PCI\SPSS\SPSS¥X图\无标题6.sav	
	¬¡动数Õu¶°	数Õu¶°1	
	过滤¾¹	<无>	
	权­«	<无>	
	©î¤À¤å¥ó	<无>	
	¤u§@数Õu¤å¥ó¤¤ªº¦æ数	45	
¯Ê¥¢­È处²z	对¯Ê¥¢ªº©w义	将¥Î户©w义ªº¯Ê¥¢­È视为¯Ê¥¢¡C	
	¨Ï¥Îªº个®×数	¨C对变¶qªº统计³£°ò¤_©Ò¦³对¤_该对变¶q¨ã¦³¦³®Ä数Õuªº个®×¡C	
语ªk	NONPAR CORR
  /VARIABLES=­°温­S围 绿¦aLPI
  /PRINT=SPEARMAN TWOTAIL NOSIG
  /MISSING=PAIRWISE.	
资·½	处²zµ{§Ç时间	00:00:00.00	
	¯Ó¥Î时间	00:00:00.00	
	¤¹许ªº个®×数	629145 个个®×a	

a. °ò¤_¤u§@ªÅ间内¦sªº¥i¥Î©Ê	


¬Û关©Ê	
	­°温­S围	绿¦aLPI	
´µ¥Ö尔°Ò Rho	­°温­S围	¬Û关¨t数	1.000	-.182	
		显µÛ©Ê¡]双§À¡^	.	.250	
		个®×数	42	42	
	绿¦aLPI	¬Û关¨t数	-.182	1.000	
		显µÛ©Ê¡]双§À¡^	.250	.	
		个®×数	42	42	

NONPAR CORR
  /VARIABLES=­°温­S围 ¤ôÊ^LPI
  /PRINT=SPEARMAN TWOTAIL NOSIG
  /MISSING=PAIRWISE.


«D参数¬Û关©Ê


备ª`	
¤w创«Ø输¥X	23-OCT-2023 21:13:18	
ª`释		
输¤J	数Õu	D:\ACCESSIBILITY AND PCI\SPSS\SPSS¥X图\无标题6.sav	
	¬¡动数Õu¶°	数Õu¶°1	
	过滤¾¹	<无>	
	权­«	<无>	
	©î¤À¤å¥ó	<无>	
	¤u§@数Õu¤å¥ó¤¤ªº¦æ数	45	
¯Ê¥¢­È处²z	对¯Ê¥¢ªº©w义	将¥Î户©w义ªº¯Ê¥¢­È视为¯Ê¥¢¡C	
	¨Ï¥Îªº个®×数	¨C对变¶qªº统计³£°ò¤_©Ò¦³对¤_该对变¶q¨ã¦³¦³®Ä数Õuªº个®×¡C	
语ªk	NONPAR CORR
  /VARIABLES=­°温­S围 ¤ôÊ^LPI
  /PRINT=SPEARMAN TWOTAIL NOSIG
  /MISSING=PAIRWISE.	
资·½	处²zµ{§Ç时间	00:00:00.00	
	¯Ó¥Î时间	00:00:00.00	
	¤¹许ªº个®×数	629145 个个®×a	

a. °ò¤_¤u§@ªÅ间内¦sªº¥i¥Î©Ê	


¬Û关©Ê	
	­°温­S围	¤ôÊ^LPI	
´µ¥Ö尔°Ò Rho	­°温­S围	¬Û关¨t数	1.000	.157	
		显µÛ©Ê¡]双§À¡^	.	.320	
		个®×数	42	42	
	¤ôÊ^LPI	¬Û关¨t数	.157	1.000	
		显µÛ©Ê¡]双§À¡^	.320	.	
		个®×数	42	42	

NONPAR CORR
  /VARIABLES=­°温­S围 ¤£³z¤ô­±LSI
  /PRINT=SPEARMAN TWOTAIL NOSIG
  /MISSING=PAIRWISE.


«D参数¬Û关©Ê


备ª`	
¤w创«Ø输¥X	23-OCT-2023 21:13:32	
ª`释		
输¤J	数Õu	D:\ACCESSIBILITY AND PCI\SPSS\SPSS¥X图\无标题6.sav	
	¬¡动数Õu¶°	数Õu¶°1	
	过滤¾¹	<无>	
	权­«	<无>	
	©î¤À¤å¥ó	<无>	
	¤u§@数Õu¤å¥ó¤¤ªº¦æ数	45	
¯Ê¥¢­È处²z	对¯Ê¥¢ªº©w义	将¥Î户©w义ªº¯Ê¥¢­È视为¯Ê¥¢¡C	
	¨Ï¥Îªº个®×数	¨C对变¶qªº统计³£°ò¤_©Ò¦³对¤_该对变¶q¨ã¦³¦³®Ä数Õuªº个®×¡C	
语ªk	NONPAR CORR
  /VARIABLES=­°温­S围 ¤£³z¤ô­±LSI
  /PRINT=SPEARMAN TWOTAIL NOSIG
  /MISSING=PAIRWISE.	
资·½	处²zµ{§Ç时间	00:00:00.00	
	¯Ó¥Î时间	00:00:00.00	
	¤¹许ªº个®×数	629145 个个®×a	

a. °ò¤_¤u§@ªÅ间内¦sªº¥i¥Î©Ê	


¬Û关©Ê	
	­°温­S围	¤£³z¤ô­±LSI	
´µ¥Ö尔°Ò Rho	­°温­S围	¬Û关¨t数	1.000	.304	
		显µÛ©Ê¡]双§À¡^	.	.050	
		个®×数	42	42	
	¤£³z¤ô­±LSI	¬Û关¨t数	.304	1.000	
		显µÛ©Ê¡]双§À¡^	.050	.	
		个®×数	42	42	

NONPAR CORR
  /VARIABLES=­°温­S围 绿¦aLSI
  /PRINT=SPEARMAN TWOTAIL NOSIG
  /MISSING=PAIRWISE.


«D参数¬Û关©Ê


备ª`	
¤w创«Ø输¥X	23-OCT-2023 21:13:46	
ª`释		
输¤J	数Õu	D:\ACCESSIBILITY AND PCI\SPSS\SPSS¥X图\无标题6.sav	
	¬¡动数Õu¶°	数Õu¶°1	
	过滤¾¹	<无>	
	权­«	<无>	
	©î¤À¤å¥ó	<无>	
	¤u§@数Õu¤å¥ó¤¤ªº¦æ数	45	
¯Ê¥¢­È处²z	对¯Ê¥¢ªº©w义	将¥Î户©w义ªº¯Ê¥¢­È视为¯Ê¥¢¡C	
	¨Ï¥Îªº个®×数	¨C对变¶qªº统计³£°ò¤_©Ò¦³对¤_该对变¶q¨ã¦³¦³®Ä数Õuªº个®×¡C	
语ªk	NONPAR CORR
  /VARIABLES=­°温­S围 绿¦aLSI
  /PRINT=SPEARMAN TWOTAIL NOSIG
  /MISSING=PAIRWISE.	
资·½	处²zµ{§Ç时间	00:00:00.00	
	¯Ó¥Î时间	00:00:00.01	
	¤¹许ªº个®×数	629145 个个®×a	

a. °ò¤_¤u§@ªÅ间内¦sªº¥i¥Î©Ê	


¬Û关©Ê	
	­°温­S围	绿¦aLSI	
´µ¥Ö尔°Ò Rho	­°温­S围	¬Û关¨t数	1.000	.385*	
		显µÛ©Ê¡]双§À¡^	.	.012	
		个®×数	42	42	
	绿¦aLSI	¬Û关¨t数	.385*	1.000	
		显µÛ©Ê¡]双§À¡^	.012	.	
		个®×数	42	42	

*. ¦b 0.05 级别¡]双§À¡^¡A¬Û关©Ê显µÛ¡C	

NONPAR CORR
  /VARIABLES=­°温­S围 ¤ôÊ^LSI
  /PRINT=SPEARMAN TWOTAIL NOSIG
  /MISSING=PAIRWISE.


«D参数¬Û关©Ê


备ª`	
¤w创«Ø输¥X	23-OCT-2023 21:14:02	
ª`释		
输¤J	数Õu	D:\ACCESSIBILITY AND PCI\SPSS\SPSS¥X图\无标题6.sav	
	¬¡动数Õu¶°	数Õu¶°1	
	过滤¾¹	<无>	
	权­«	<无>	
	©î¤À¤å¥ó	<无>	
	¤u§@数Õu¤å¥ó¤¤ªº¦æ数	45	
¯Ê¥¢­È处²z	对¯Ê¥¢ªº©w义	将¥Î户©w义ªº¯Ê¥¢­È视为¯Ê¥¢¡C	
	¨Ï¥Îªº个®×数	¨C对变¶qªº统计³£°ò¤_©Ò¦³对¤_该对变¶q¨ã¦³¦³®Ä数Õuªº个®×¡C	
语ªk	NONPAR CORR
  /VARIABLES=­°温­S围 ¤ôÊ^LSI
  /PRINT=SPEARMAN TWOTAIL NOSIG
  /MISSING=PAIRWISE.	
资·½	处²zµ{§Ç时间	00:00:00.00	
	¯Ó¥Î时间	00:00:00.00	
	¤¹许ªº个®×数	629145 个个®×a	

a. °ò¤_¤u§@ªÅ间内¦sªº¥i¥Î©Ê	


¬Û关©Ê	
	­°温­S围	¤ôÊ^LSI	
´µ¥Ö尔°Ò Rho	­°温­S围	¬Û关¨t数	1.000	.283	
		显µÛ©Ê¡]双§À¡^	.	.069	
		个®×数	42	42	
	¤ôÊ^LSI	¬Û关¨t数	.283	1.000	
		显µÛ©Ê¡]双§À¡^	.069	.	
		个®×数	42	42	

NONPAR CORR
  /VARIABLES=­°温­S围 ¤£³z¤ô­±DIVISION
  /PRINT=SPEARMAN TWOTAIL NOSIG
  /MISSING=PAIRWISE.


«D参数¬Û关©Ê


备ª`	
¤w创«Ø输¥X	23-OCT-2023 21:14:12	
ª`释		
输¤J	数Õu	D:\ACCESSIBILITY AND PCI\SPSS\SPSS¥X图\无标题6.sav	
	¬¡动数Õu¶°	数Õu¶°1	
	过滤¾¹	<无>	
	权­«	<无>	
	©î¤À¤å¥ó	<无>	
	¤u§@数Õu¤å¥ó¤¤ªº¦æ数	45	
¯Ê¥¢­È处²z	对¯Ê¥¢ªº©w义	将¥Î户©w义ªº¯Ê¥¢­È视为¯Ê¥¢¡C	
	¨Ï¥Îªº个®×数	¨C对变¶qªº统计³£°ò¤_©Ò¦³对¤_该对变¶q¨ã¦³¦³®Ä数Õuªº个®×¡C	
语ªk	NONPAR CORR
  /VARIABLES=­°温­S围 ¤£³z¤ô­±DIVISION
  /PRINT=SPEARMAN TWOTAIL NOSIG
  /MISSING=PAIRWISE.	
资·½	处²zµ{§Ç时间	00:00:00.00	
	¯Ó¥Î时间	00:00:00.01	
	¤¹许ªº个®×数	629145 个个®×a	

a. °ò¤_¤u§@ªÅ间内¦sªº¥i¥Î©Ê	


¬Û关©Ê	
	­°温­S围	¤£³z¤ô­±DIVISION	
´µ¥Ö尔°Ò Rho	­°温­S围	¬Û关¨t数	1.000	.179	
		显µÛ©Ê¡]双§À¡^	.	.258	
		个®×数	42	42	
	¤£³z¤ô­±DIVISION	¬Û关¨t数	.179	1.000	
		显µÛ©Ê¡]双§À¡^	.258	.	
		个®×数	42	42	

NONPAR CORR
  /VARIABLES=­°温­S围 绿¦aDIVISION
  /PRINT=SPEARMAN TWOTAIL NOSIG
  /MISSING=PAIRWISE.


«D参数¬Û关©Ê


备ª`	
¤w创«Ø输¥X	23-OCT-2023 21:14:25	
ª`释		
输¤J	数Õu	D:\ACCESSIBILITY AND PCI\SPSS\SPSS¥X图\无标题6.sav	
	¬¡动数Õu¶°	数Õu¶°1	
	过滤¾¹	<无>	
	权­«	<无>	
	©î¤À¤å¥ó	<无>	
	¤u§@数Õu¤å¥ó¤¤ªº¦æ数	45	
¯Ê¥¢­È处²z	对¯Ê¥¢ªº©w义	将¥Î户©w义ªº¯Ê¥¢­È视为¯Ê¥¢¡C	
	¨Ï¥Îªº个®×数	¨C对变¶qªº统计³£°ò¤_©Ò¦³对¤_该对变¶q¨ã¦³¦³®Ä数Õuªº个®×¡C	
语ªk	NONPAR CORR
  /VARIABLES=­°温­S围 绿¦aDIVISION
  /PRINT=SPEARMAN TWOTAIL NOSIG
  /MISSING=PAIRWISE.	
资·½	处²zµ{§Ç时间	00:00:00.00	
	¯Ó¥Î时间	00:00:00.00	
	¤¹许ªº个®×数	629145 个个®×a	

a. °ò¤_¤u§@ªÅ间内¦sªº¥i¥Î©Ê	


¬Û关©Ê	
	­°温­S围	绿¦aDIVISION	
´µ¥Ö尔°Ò Rho	­°温­S围	¬Û关¨t数	1.000	.195	
		显µÛ©Ê¡]双§À¡^	.	.216	
		个®×数	42	42	
	绿¦aDIVISION	¬Û关¨t数	.195	1.000	
		显µÛ©Ê¡]双§À¡^	.216	.	
		个®×数	42	42	

NONPAR CORR
  /VARIABLES=­°温­S围 ¤ôÊ^DIVISION
  /PRINT=SPEARMAN TWOTAIL NOSIG
  /MISSING=PAIRWISE.


«D参数¬Û关©Ê


备ª`	
¤w创«Ø输¥X	23-OCT-2023 21:14:43	
ª`释		
输¤J	数Õu	D:\ACCESSIBILITY AND PCI\SPSS\SPSS¥X图\无标题6.sav	
	¬¡动数Õu¶°	数Õu¶°1	
	过滤¾¹	<无>	
	权­«	<无>	
	©î¤À¤å¥ó	<无>	
	¤u§@数Õu¤å¥ó¤¤ªº¦æ数	45	
¯Ê¥¢­È处²z	对¯Ê¥¢ªº©w义	将¥Î户©w义ªº¯Ê¥¢­È视为¯Ê¥¢¡C	
	¨Ï¥Îªº个®×数	¨C对变¶qªº统计³£°ò¤_©Ò¦³对¤_该对变¶q¨ã¦³¦³®Ä数Õuªº个®×¡C	
语ªk	NONPAR CORR
  /VARIABLES=­°温­S围 ¤ôÊ^DIVISION
  /PRINT=SPEARMAN TWOTAIL NOSIG
  /MISSING=PAIRWISE.	
资·½	处²zµ{§Ç时间	00:00:00.00	
	¯Ó¥Î时间	00:00:00.00	
	¤¹许ªº个®×数	629145 个个®×a	

a. °ò¤_¤u§@ªÅ间内¦sªº¥i¥Î©Ê	


¬Û关©Ê	
	­°温­S围	¤ôÊ^DIVISION	
´µ¥Ö尔°Ò Rho	­°温­S围	¬Û关¨t数	1.000	-.100	
		显µÛ©Ê¡]双§À¡^	.	.528	
		个®×数	42	42	
	¤ôÊ^DIVISION	¬Û关¨t数	-.100	1.000	
		显µÛ©Ê¡]双§À¡^	.528	.	
		个®×数	42	42	

NONPAR CORR
  /VARIABLES=­°温­S围 ¤£³z¤ô­±SPLIT
  /PRINT=SPEARMAN TWOTAIL NOSIG
  /MISSING=PAIRWISE.


«D参数¬Û关©Ê


备ª`	
¤w创«Ø输¥X	23-OCT-2023 21:15:01	
ª`释		
输¤J	数Õu	D:\ACCESSIBILITY AND PCI\SPSS\SPSS¥X图\无标题6.sav	
	¬¡动数Õu¶°	数Õu¶°1	
	过滤¾¹	<无>	
	权­«	<无>	
	©î¤À¤å¥ó	<无>	
	¤u§@数Õu¤å¥ó¤¤ªº¦æ数	45	
¯Ê¥¢­È处²z	对¯Ê¥¢ªº©w义	将¥Î户©w义ªº¯Ê¥¢­È视为¯Ê¥¢¡C	
	¨Ï¥Îªº个®×数	¨C对变¶qªº统计³£°ò¤_©Ò¦³对¤_该对变¶q¨ã¦³¦³®Ä数Õuªº个®×¡C	
语ªk	NONPAR CORR
  /VARIABLES=­°温­S围 ¤£³z¤ô­±SPLIT
  /PRINT=SPEARMAN TWOTAIL NOSIG
  /MISSING=PAIRWISE.	
资·½	处²zµ{§Ç时间	00:00:00.00	
	¯Ó¥Î时间	00:00:00.00	
	¤¹许ªº个®×数	629145 个个®×a	

a. °ò¤_¤u§@ªÅ间内¦sªº¥i¥Î©Ê	


¬Û关©Ê	
	­°温­S围	¤£³z¤ô­±SPLIT	
´µ¥Ö尔°Ò Rho	­°温­S围	¬Û关¨t数	1.000	.148	
		显µÛ©Ê¡]双§À¡^	.	.348	
		个®×数	42	42	
	¤£³z¤ô­±SPLIT	¬Û关¨t数	.148	1.000	
		显µÛ©Ê¡]双§À¡^	.348	.	
		个®×数	42	42	

NONPAR CORR
  /VARIABLES=­°温­S围 绿¦aSPLIT
  /PRINT=SPEARMAN TWOTAIL NOSIG
  /MISSING=PAIRWISE.


«D参数¬Û关©Ê


备ª`	
¤w创«Ø输¥X	23-OCT-2023 21:15:13	
ª`释		
输¤J	数Õu	D:\ACCESSIBILITY AND PCI\SPSS\SPSS¥X图\无标题6.sav	
	¬¡动数Õu¶°	数Õu¶°1	
	过滤¾¹	<无>	
	权­«	<无>	
	©î¤À¤å¥ó	<无>	
	¤u§@数Õu¤å¥ó¤¤ªº¦æ数	45	
¯Ê¥¢­È处²z	对¯Ê¥¢ªº©w义	将¥Î户©w义ªº¯Ê¥¢­È视为¯Ê¥¢¡C	
	¨Ï¥Îªº个®×数	¨C对变¶qªº统计³£°ò¤_©Ò¦³对¤_该对变¶q¨ã¦³¦³®Ä数Õuªº个®×¡C	
语ªk	NONPAR CORR
  /VARIABLES=­°温­S围 绿¦aSPLIT
  /PRINT=SPEARMAN TWOTAIL NOSIG
  /MISSING=PAIRWISE.	
资·½	处²zµ{§Ç时间	00:00:00.00	
	¯Ó¥Î时间	00:00:00.00	
	¤¹许ªº个®×数	629145 个个®×a	

a. °ò¤_¤u§@ªÅ间内¦sªº¥i¥Î©Ê	


¬Û关©Ê	
	­°温­S围	绿¦aSPLIT	
´µ¥Ö尔°Ò Rho	­°温­S围	¬Û关¨t数	1.000	.192	
		显µÛ©Ê¡]双§À¡^	.	.223	
		个®×数	42	42	
	绿¦aSPLIT	¬Û关¨t数	.192	1.000	
		显µÛ©Ê¡]双§À¡^	.223	.	
		个®×数	42	42	

NONPAR CORR
  /VARIABLES=­°温­S围 ¤ôÊ^SPLIT
  /PRINT=SPEARMAN TWOTAIL NOSIG
  /MISSING=PAIRWISE.


«D参数¬Û关©Ê


备ª`	
¤w创«Ø输¥X	23-OCT-2023 21:15:24	
ª`释		
输¤J	数Õu	D:\ACCESSIBILITY AND PCI\SPSS\SPSS¥X图\无标题6.sav	
	¬¡动数Õu¶°	数Õu¶°1	
	过滤¾¹	<无>	
	权­«	<无>	
	©î¤À¤å¥ó	<无>	
	¤u§@数Õu¤å¥ó¤¤ªº¦æ数	45	
¯Ê¥¢­È处²z	对¯Ê¥¢ªº©w义	将¥Î户©w义ªº¯Ê¥¢­È视为¯Ê¥¢¡C	
	¨Ï¥Îªº个®×数	¨C对变¶qªº统计³£°ò¤_©Ò¦³对¤_该对变¶q¨ã¦³¦³®Ä数Õuªº个®×¡C	
语ªk	NONPAR CORR
  /VARIABLES=­°温­S围 ¤ôÊ^SPLIT
  /PRINT=SPEARMAN TWOTAIL NOSIG
  /MISSING=PAIRWISE.	
资·½	处²zµ{§Ç时间	00:00:00.00	
	¯Ó¥Î时间	00:00:00.00	
	¤¹许ªº个®×数	629145 个个®×a	

a. °ò¤_¤u§@ªÅ间内¦sªº¥i¥Î©Ê	


¬Û关©Ê	
	­°温­S围	¤ôÊ^SPLIT	
´µ¥Ö尔°Ò Rho	­°温­S围	¬Û关¨t数	1.000	-.132	
		显µÛ©Ê¡]双§À¡^	.	.406	
		个®×数	42	42	
	¤ôÊ^SPLIT	¬Û关¨t数	-.132	1.000	
		显µÛ©Ê¡]双§À¡^	.406	.	
		个®×数	42	42	

NONPAR CORR
  /VARIABLES=­°温­S围 ¤£³z¤ô­±AI
  /PRINT=SPEARMAN TWOTAIL NOSIG
  /MISSING=PAIRWISE.


«D参数¬Û关©Ê


备ª`	
¤w创«Ø输¥X	23-OCT-2023 21:15:34	
ª`释		
输¤J	数Õu	D:\ACCESSIBILITY AND PCI\SPSS\SPSS¥X图\无标题6.sav	
	¬¡动数Õu¶°	数Õu¶°1	
	过滤¾¹	<无>	
	权­«	<无>	
	©î¤À¤å¥ó	<无>	
	¤u§@数Õu¤å¥ó¤¤ªº¦æ数	45	
¯Ê¥¢­È处²z	对¯Ê¥¢ªº©w义	将¥Î户©w义ªº¯Ê¥¢­È视为¯Ê¥¢¡C	
	¨Ï¥Îªº个®×数	¨C对变¶qªº统计³£°ò¤_©Ò¦³对¤_该对变¶q¨ã¦³¦³®Ä数Õuªº个®×¡C	
语ªk	NONPAR CORR
  /VARIABLES=­°温­S围 ¤£³z¤ô­±AI
  /PRINT=SPEARMAN TWOTAIL NOSIG
  /MISSING=PAIRWISE.	
资·½	处²zµ{§Ç时间	00:00:00.00	
	¯Ó¥Î时间	00:00:00.00	
	¤¹许ªº个®×数	629145 个个®×a	

a. °ò¤_¤u§@ªÅ间内¦sªº¥i¥Î©Ê	


¬Û关©Ê	
	­°温­S围	¤£³z¤ô­±AI	
´µ¥Ö尔°Ò Rho	­°温­S围	¬Û关¨t数	1.000	.084	
		显µÛ©Ê¡]双§À¡^	.	.596	
		个®×数	42	42	
	¤£³z¤ô­±AI	¬Û关¨t数	.084	1.000	
		显µÛ©Ê¡]双§À¡^	.596	.	
		个®×数	42	42	

NONPAR CORR
  /VARIABLES=­°温­S围 绿¦aAI
  /PRINT=SPEARMAN TWOTAIL NOSIG
  /MISSING=PAIRWISE.


«D参数¬Û关©Ê


备ª`	
¤w创«Ø输¥X	23-OCT-2023 21:15:44	
ª`释		
输¤J	数Õu	D:\ACCESSIBILITY AND PCI\SPSS\SPSS¥X图\无标题6.sav	
	¬¡动数Õu¶°	数Õu¶°1	
	过滤¾¹	<无>	
	权­«	<无>	
	©î¤À¤å¥ó	<无>	
	¤u§@数Õu¤å¥ó¤¤ªº¦æ数	45	
¯Ê¥¢­È处²z	对¯Ê¥¢ªº©w义	将¥Î户©w义ªº¯Ê¥¢­È视为¯Ê¥¢¡C	
	¨Ï¥Îªº个®×数	¨C对变¶qªº统计³£°ò¤_©Ò¦³对¤_该对变¶q¨ã¦³¦³®Ä数Õuªº个®×¡C	
语ªk	NONPAR CORR
  /VARIABLES=­°温­S围 绿¦aAI
  /PRINT=SPEARMAN TWOTAIL NOSIG
  /MISSING=PAIRWISE.	
资·½	处²zµ{§Ç时间	00:00:00.00	
	¯Ó¥Î时间	00:00:00.00	
	¤¹许ªº个®×数	629145 个个®×a	

a. °ò¤_¤u§@ªÅ间内¦sªº¥i¥Î©Ê	


¬Û关©Ê	
	­°温­S围	绿¦aAI	
´µ¥Ö尔°Ò Rho	­°温­S围	¬Û关¨t数	1.000	-.011	
		显µÛ©Ê¡]双§À¡^	.	.943	
		个®×数	42	42	
	绿¦aAI	¬Û关¨t数	-.011	1.000	
		显µÛ©Ê¡]双§À¡^	.943	.	
		个®×数	42	42	

NONPAR CORR
  /VARIABLES=­°温­S围 ¤ôÊ^AI
  /PRINT=SPEARMAN TWOTAIL NOSIG
  /MISSING=PAIRWISE.


«D参数¬Û关©Ê


备ª`	
¤w创«Ø输¥X	23-OCT-2023 21:15:56	
ª`释		
输¤J	数Õu	D:\ACCESSIBILITY AND PCI\SPSS\SPSS¥X图\无标题6.sav	
	¬¡动数Õu¶°	数Õu¶°1	
	过滤¾¹	<无>	
	权­«	<无>	
	©î¤À¤å¥ó	<无>	
	¤u§@数Õu¤å¥ó¤¤ªº¦æ数	45	
¯Ê¥¢­È处²z	对¯Ê¥¢ªº©w义	将¥Î户©w义ªº¯Ê¥¢­È视为¯Ê¥¢¡C	
	¨Ï¥Îªº个®×数	¨C对变¶qªº统计³£°ò¤_©Ò¦³对¤_该对变¶q¨ã¦³¦³®Ä数Õuªº个®×¡C	
语ªk	NONPAR CORR
  /VARIABLES=­°温­S围 ¤ôÊ^AI
  /PRINT=SPEARMAN TWOTAIL NOSIG
  /MISSING=PAIRWISE.	
资·½	处²zµ{§Ç时间	00:00:00.00	
	¯Ó¥Î时间	00:00:00.00	
	¤¹许ªº个®×数	629145 个个®×a	

a. °ò¤_¤u§@ªÅ间内¦sªº¥i¥Î©Ê	


¬Û关©Ê	
	­°温­S围	¤ôÊ^AI	
´µ¥Ö尔°Ò Rho	­°温­S围	¬Û关¨t数	1.000	.190	
		显µÛ©Ê¡]双§À¡^	.	.228	
		个®×数	42	42	
	¤ôÊ^AI	¬Û关¨t数	.190	1.000	
		显µÛ©Ê¡]双§À¡^	.228	.	
		个®×数	42	42	

NONPAR CORR
  /VARIABLES=­°温´T«× ¤£³z¤ô­±PD
  /PRINT=SPEARMAN TWOTAIL NOSIG
  /MISSING=PAIRWISE.


«D参数¬Û关©Ê


备ª`	
¤w创«Ø输¥X	23-OCT-2023 21:16:17	
ª`释		
输¤J	数Õu	D:\ACCESSIBILITY AND PCI\SPSS\SPSS¥X图\无标题6.sav	
	¬¡动数Õu¶°	数Õu¶°1	
	过滤¾¹	<无>	
	权­«	<无>	
	©î¤À¤å¥ó	<无>	
	¤u§@数Õu¤å¥ó¤¤ªº¦æ数	45	
¯Ê¥¢­È处²z	对¯Ê¥¢ªº©w义	将¥Î户©w义ªº¯Ê¥¢­È视为¯Ê¥¢¡C	
	¨Ï¥Îªº个®×数	¨C对变¶qªº统计³£°ò¤_©Ò¦³对¤_该对变¶q¨ã¦³¦³®Ä数Õuªº个®×¡C	
语ªk	NONPAR CORR
  /VARIABLES=­°温´T«× ¤£³z¤ô­±PD
  /PRINT=SPEARMAN TWOTAIL NOSIG
  /MISSING=PAIRWISE.	
资·½	处²zµ{§Ç时间	00:00:00.00	
	¯Ó¥Î时间	00:00:00.00	
	¤¹许ªº个®×数	629145 个个®×a	

a. °ò¤_¤u§@ªÅ间内¦sªº¥i¥Î©Ê	


¬Û关©Ê	
	­°温´T«×	¤£³z¤ô­±PD	
´µ¥Ö尔°Ò Rho	­°温´T«×	¬Û关¨t数	1.000	-.009	
		显µÛ©Ê¡]双§À¡^	.	.953	
		个®×数	42	42	
	¤£³z¤ô­±PD	¬Û关¨t数	-.009	1.000	
		显µÛ©Ê¡]双§À¡^	.953	.	
		个®×数	42	42	

NONPAR CORR
  /VARIABLES=­°温´T«× 绿¦aPD
  /PRINT=SPEARMAN TWOTAIL NOSIG
  /MISSING=PAIRWISE.


«D参数¬Û关©Ê


备ª`	
¤w创«Ø输¥X	23-OCT-2023 21:16:36	
ª`释		
输¤J	数Õu	D:\ACCESSIBILITY AND PCI\SPSS\SPSS¥X图\无标题6.sav	
	¬¡动数Õu¶°	数Õu¶°1	
	过滤¾¹	<无>	
	权­«	<无>	
	©î¤À¤å¥ó	<无>	
	¤u§@数Õu¤å¥ó¤¤ªº¦æ数	45	
¯Ê¥¢­È处²z	对¯Ê¥¢ªº©w义	将¥Î户©w义ªº¯Ê¥¢­È视为¯Ê¥¢¡C	
	¨Ï¥Îªº个®×数	¨C对变¶qªº统计³£°ò¤_©Ò¦³对¤_该对变¶q¨ã¦³¦³®Ä数Õuªº个®×¡C	
语ªk	NONPAR CORR
  /VARIABLES=­°温´T«× 绿¦aPD
  /PRINT=SPEARMAN TWOTAIL NOSIG
  /MISSING=PAIRWISE.	
资·½	处²zµ{§Ç时间	00:00:00.00	
	¯Ó¥Î时间	00:00:00.00	
	¤¹许ªº个®×数	629145 个个®×a	

a. °ò¤_¤u§@ªÅ间内¦sªº¥i¥Î©Ê	


¬Û关©Ê	
	­°温´T«×	绿¦aPD	
´µ¥Ö尔°Ò Rho	­°温´T«×	¬Û关¨t数	1.000	.118	
		显µÛ©Ê¡]双§À¡^	.	.458	
		个®×数	42	42	
	绿¦aPD	¬Û关¨t数	.118	1.000	
		显µÛ©Ê¡]双§À¡^	.458	.	
		个®×数	42	42	

NONPAR CORR
  /VARIABLES=­°温´T«× ¤ôÊ^PD
  /PRINT=SPEARMAN TWOTAIL NOSIG
  /MISSING=PAIRWISE.


«D参数¬Û关©Ê


备ª`	
¤w创«Ø输¥X	23-OCT-2023 21:16:47	
ª`释		
输¤J	数Õu	D:\ACCESSIBILITY AND PCI\SPSS\SPSS¥X图\无标题6.sav	
	¬¡动数Õu¶°	数Õu¶°1	
	过滤¾¹	<无>	
	权­«	<无>	
	©î¤À¤å¥ó	<无>	
	¤u§@数Õu¤å¥ó¤¤ªº¦æ数	45	
¯Ê¥¢­È处²z	对¯Ê¥¢ªº©w义	将¥Î户©w义ªº¯Ê¥¢­È视为¯Ê¥¢¡C	
	¨Ï¥Îªº个®×数	¨C对变¶qªº统计³£°ò¤_©Ò¦³对¤_该对变¶q¨ã¦³¦³®Ä数Õuªº个®×¡C	
语ªk	NONPAR CORR
  /VARIABLES=­°温´T«× ¤ôÊ^PD
  /PRINT=SPEARMAN TWOTAIL NOSIG
  /MISSING=PAIRWISE.	
资·½	处²zµ{§Ç时间	00:00:00.00	
	¯Ó¥Î时间	00:00:00.01	
	¤¹许ªº个®×数	629145 个个®×a	

a. °ò¤_¤u§@ªÅ间内¦sªº¥i¥Î©Ê	


¬Û关©Ê	
	­°温´T«×	¤ôÊ^PD	
´µ¥Ö尔°Ò Rho	­°温´T«×	¬Û关¨t数	1.000	-.114	
		显µÛ©Ê¡]双§À¡^	.	.470	
		个®×数	42	42	
	¤ôÊ^PD	¬Û关¨t数	-.114	1.000	
		显µÛ©Ê¡]双§À¡^	.470	.	
		个®×数	42	42	

NONPAR CORR
  /VARIABLES=­°温´T«× ¤£³z¤ô­±LPI
  /PRINT=SPEARMAN TWOTAIL NOSIG
  /MISSING=PAIRWISE.


«D参数¬Û关©Ê


备ª`	
¤w创«Ø输¥X	23-OCT-2023 21:17:01	
ª`释		
输¤J	数Õu	D:\ACCESSIBILITY AND PCI\SPSS\SPSS¥X图\无标题6.sav	
	¬¡动数Õu¶°	数Õu¶°1	
	过滤¾¹	<无>	
	权­«	<无>	
	©î¤À¤å¥ó	<无>	
	¤u§@数Õu¤å¥ó¤¤ªº¦æ数	45	
¯Ê¥¢­È处²z	对¯Ê¥¢ªº©w义	将¥Î户©w义ªº¯Ê¥¢­È视为¯Ê¥¢¡C	
	¨Ï¥Îªº个®×数	¨C对变¶qªº统计³£°ò¤_©Ò¦³对¤_该对变¶q¨ã¦³¦³®Ä数Õuªº个®×¡C	
语ªk	NONPAR CORR
  /VARIABLES=­°温´T«× ¤£³z¤ô­±LPI
  /PRINT=SPEARMAN TWOTAIL NOSIG
  /MISSING=PAIRWISE.	
资·½	处²zµ{§Ç时间	00:00:00.00	
	¯Ó¥Î时间	00:00:00.00	
	¤¹许ªº个®×数	629145 个个®×a	

a. °ò¤_¤u§@ªÅ间内¦sªº¥i¥Î©Ê	


¬Û关©Ê	
	­°温´T«×	¤£³z¤ô­±LPI	
´µ¥Ö尔°Ò Rho	­°温´T«×	¬Û关¨t数	1.000	.036	
		显µÛ©Ê¡]双§À¡^	.	.819	
		个®×数	42	42	
	¤£³z¤ô­±LPI	¬Û关¨t数	.036	1.000	
		显µÛ©Ê¡]双§À¡^	.819	.	
		个®×数	42	42	

NONPAR CORR
  /VARIABLES=­°温´T«× 绿¦aLPI
  /PRINT=SPEARMAN TWOTAIL NOSIG
  /MISSING=PAIRWISE.


«D参数¬Û关©Ê


备ª`	
¤w创«Ø输¥X	23-OCT-2023 21:17:11	
ª`释		
输¤J	数Õu	D:\ACCESSIBILITY AND PCI\SPSS\SPSS¥X图\无标题6.sav	
	¬¡动数Õu¶°	数Õu¶°1	
	过滤¾¹	<无>	
	权­«	<无>	
	©î¤À¤å¥ó	<无>	
	¤u§@数Õu¤å¥ó¤¤ªº¦æ数	45	
¯Ê¥¢­È处²z	对¯Ê¥¢ªº©w义	将¥Î户©w义ªº¯Ê¥¢­È视为¯Ê¥¢¡C	
	¨Ï¥Îªº个®×数	¨C对变¶qªº统计³£°ò¤_©Ò¦³对¤_该对变¶q¨ã¦³¦³®Ä数Õuªº个®×¡C	
语ªk	NONPAR CORR
  /VARIABLES=­°温´T«× 绿¦aLPI
  /PRINT=SPEARMAN TWOTAIL NOSIG
  /MISSING=PAIRWISE.	
资·½	处²zµ{§Ç时间	00:00:00.03	
	¯Ó¥Î时间	00:00:00.01	
	¤¹许ªº个®×数	629145 个个®×a	

a. °ò¤_¤u§@ªÅ间内¦sªº¥i¥Î©Ê	


¬Û关©Ê	
	­°温´T«×	绿¦aLPI	
´µ¥Ö尔°Ò Rho	­°温´T«×	¬Û关¨t数	1.000	-.161	
		显µÛ©Ê¡]双§À¡^	.	.308	
		个®×数	42	42	
	绿¦aLPI	¬Û关¨t数	-.161	1.000	
		显µÛ©Ê¡]双§À¡^	.308	.	
		个®×数	42	42	

NONPAR CORR
  /VARIABLES=­°温´T«× ¤ôÊ^LPI
  /PRINT=SPEARMAN TWOTAIL NOSIG
  /MISSING=PAIRWISE.


«D参数¬Û关©Ê


备ª`	
¤w创«Ø输¥X	23-OCT-2023 21:17:24	
ª`释		
输¤J	数Õu	D:\ACCESSIBILITY AND PCI\SPSS\SPSS¥X图\无标题6.sav	
	¬¡动数Õu¶°	数Õu¶°1	
	过滤¾¹	<无>	
	权­«	<无>	
	©î¤À¤å¥ó	<无>	
	¤u§@数Õu¤å¥ó¤¤ªº¦æ数	45	
¯Ê¥¢­È处²z	对¯Ê¥¢ªº©w义	将¥Î户©w义ªº¯Ê¥¢­È视为¯Ê¥¢¡C	
	¨Ï¥Îªº个®×数	¨C对变¶qªº统计³£°ò¤_©Ò¦³对¤_该对变¶q¨ã¦³¦³®Ä数Õuªº个®×¡C	
语ªk	NONPAR CORR
  /VARIABLES=­°温´T«× ¤ôÊ^LPI
  /PRINT=SPEARMAN TWOTAIL NOSIG
  /MISSING=PAIRWISE.	
资·½	处²zµ{§Ç时间	00:00:00.00	
	¯Ó¥Î时间	00:00:00.01	
	¤¹许ªº个®×数	629145 个个®×a	

a. °ò¤_¤u§@ªÅ间内¦sªº¥i¥Î©Ê	


¬Û关©Ê	
	­°温´T«×	¤ôÊ^LPI	
´µ¥Ö尔°Ò Rho	­°温´T«×	¬Û关¨t数	1.000	.152	
		显µÛ©Ê¡]双§À¡^	.	.335	
		个®×数	42	42	
	¤ôÊ^LPI	¬Û关¨t数	.152	1.000	
		显µÛ©Ê¡]双§À¡^	.335	.	
		个®×数	42	42	

NONPAR CORR
  /VARIABLES=­°温´T«× ¤£³z¤ô­±LSI
  /PRINT=SPEARMAN TWOTAIL NOSIG
  /MISSING=PAIRWISE.


«D参数¬Û关©Ê


备ª`	
¤w创«Ø输¥X	23-OCT-2023 21:17:43	
ª`释		
输¤J	数Õu	D:\ACCESSIBILITY AND PCI\SPSS\SPSS¥X图\无标题6.sav	
	¬¡动数Õu¶°	数Õu¶°1	
	过滤¾¹	<无>	
	权­«	<无>	
	©î¤À¤å¥ó	<无>	
	¤u§@数Õu¤å¥ó¤¤ªº¦æ数	45	
¯Ê¥¢­È处²z	对¯Ê¥¢ªº©w义	将¥Î户©w义ªº¯Ê¥¢­È视为¯Ê¥¢¡C	
	¨Ï¥Îªº个®×数	¨C对变¶qªº统计³£°ò¤_©Ò¦³对¤_该对变¶q¨ã¦³¦³®Ä数Õuªº个®×¡C	
语ªk	NONPAR CORR
  /VARIABLES=­°温´T«× ¤£³z¤ô­±LSI
  /PRINT=SPEARMAN TWOTAIL NOSIG
  /MISSING=PAIRWISE.	
资·½	处²zµ{§Ç时间	00:00:00.02	
	¯Ó¥Î时间	00:00:00.00	
	¤¹许ªº个®×数	629145 个个®×a	

a. °ò¤_¤u§@ªÅ间内¦sªº¥i¥Î©Ê	


¬Û关©Ê	
	­°温´T«×	¤£³z¤ô­±LSI	
´µ¥Ö尔°Ò Rho	­°温´T«×	¬Û关¨t数	1.000	-.058	
		显µÛ©Ê¡]双§À¡^	.	.714	
		个®×数	42	42	
	¤£³z¤ô­±LSI	¬Û关¨t数	-.058	1.000	
		显µÛ©Ê¡]双§À¡^	.714	.	
		个®×数	42	42	

NONPAR CORR
  /VARIABLES=­°温´T«× 绿¦aLSI
  /PRINT=SPEARMAN TWOTAIL NOSIG
  /MISSING=PAIRWISE.


«D参数¬Û关©Ê


备ª`	
¤w创«Ø输¥X	23-OCT-2023 21:17:54	
ª`释		
输¤J	数Õu	D:\ACCESSIBILITY AND PCI\SPSS\SPSS¥X图\无标题6.sav	
	¬¡动数Õu¶°	数Õu¶°1	
	过滤¾¹	<无>	
	权­«	<无>	
	©î¤À¤å¥ó	<无>	
	¤u§@数Õu¤å¥ó¤¤ªº¦æ数	45	
¯Ê¥¢­È处²z	对¯Ê¥¢ªº©w义	将¥Î户©w义ªº¯Ê¥¢­È视为¯Ê¥¢¡C	
	¨Ï¥Îªº个®×数	¨C对变¶qªº统计³£°ò¤_©Ò¦³对¤_该对变¶q¨ã¦³¦³®Ä数Õuªº个®×¡C	
语ªk	NONPAR CORR
  /VARIABLES=­°温´T«× 绿¦aLSI
  /PRINT=SPEARMAN TWOTAIL NOSIG
  /MISSING=PAIRWISE.	
资·½	处²zµ{§Ç时间	00:00:00.02	
	¯Ó¥Î时间	00:00:00.01	
	¤¹许ªº个®×数	629145 个个®×a	

a. °ò¤_¤u§@ªÅ间内¦sªº¥i¥Î©Ê	


¬Û关©Ê	
	­°温´T«×	绿¦aLSI	
´µ¥Ö尔°Ò Rho	­°温´T«×	¬Û关¨t数	1.000	.126	
		显µÛ©Ê¡]双§À¡^	.	.427	
		个®×数	42	42	
	绿¦aLSI	¬Û关¨t数	.126	1.000	
		显µÛ©Ê¡]双§À¡^	.427	.	
		个®×数	42	42	

NONPAR CORR
  /VARIABLES=­°温´T«× ¤ôÊ^LSI
  /PRINT=SPEARMAN TWOTAIL NOSIG
  /MISSING=PAIRWISE.


«D参数¬Û关©Ê


备ª`	
¤w创«Ø输¥X	23-OCT-2023 21:18:04	
ª`释		
输¤J	数Õu	D:\ACCESSIBILITY AND PCI\SPSS\SPSS¥X图\无标题6.sav	
	¬¡动数Õu¶°	数Õu¶°1	
	过滤¾¹	<无>	
	权­«	<无>	
	©î¤À¤å¥ó	<无>	
	¤u§@数Õu¤å¥ó¤¤ªº¦æ数	45	
¯Ê¥¢­È处²z	对¯Ê¥¢ªº©w义	将¥Î户©w义ªº¯Ê¥¢­È视为¯Ê¥¢¡C	
	¨Ï¥Îªº个®×数	¨C对变¶qªº统计³£°ò¤_©Ò¦³对¤_该对变¶q¨ã¦³¦³®Ä数Õuªº个®×¡C	
语ªk	NONPAR CORR
  /VARIABLES=­°温´T«× ¤ôÊ^LSI
  /PRINT=SPEARMAN TWOTAIL NOSIG
  /MISSING=PAIRWISE.	
资·½	处²zµ{§Ç时间	00:00:00.02	
	¯Ó¥Î时间	00:00:00.00	
	¤¹许ªº个®×数	629145 个个®×a	

a. °ò¤_¤u§@ªÅ间内¦sªº¥i¥Î©Ê	


¬Û关©Ê	
	­°温´T«×	¤ôÊ^LSI	
´µ¥Ö尔°Ò Rho	­°温´T«×	¬Û关¨t数	1.000	-.100	
		显µÛ©Ê¡]双§À¡^	.	.528	
		个®×数	42	42	
	¤ôÊ^LSI	¬Û关¨t数	-.100	1.000	
		显µÛ©Ê¡]双§À¡^	.528	.	
		个®×数	42	42	

NONPAR CORR
  /VARIABLES=­°温´T«× ¤£³z¤ô­±DIVISION
  /PRINT=SPEARMAN TWOTAIL NOSIG
  /MISSING=PAIRWISE.


«D参数¬Û关©Ê


备ª`	
¤w创«Ø输¥X	23-OCT-2023 21:18:18	
ª`释		
输¤J	数Õu	D:\ACCESSIBILITY AND PCI\SPSS\SPSS¥X图\无标题6.sav	
	¬¡动数Õu¶°	数Õu¶°1	
	过滤¾¹	<无>	
	权­«	<无>	
	©î¤À¤å¥ó	<无>	
	¤u§@数Õu¤å¥ó¤¤ªº¦æ数	45	
¯Ê¥¢­È处²z	对¯Ê¥¢ªº©w义	将¥Î户©w义ªº¯Ê¥¢­È视为¯Ê¥¢¡C	
	¨Ï¥Îªº个®×数	¨C对变¶qªº统计³£°ò¤_©Ò¦³对¤_该对变¶q¨ã¦³¦³®Ä数Õuªº个®×¡C	
语ªk	NONPAR CORR
  /VARIABLES=­°温´T«× ¤£³z¤ô­±DIVISION
  /PRINT=SPEARMAN TWOTAIL NOSIG
  /MISSING=PAIRWISE.	
资·½	处²zµ{§Ç时间	00:00:00.02	
	¯Ó¥Î时间	00:00:00.00	
	¤¹许ªº个®×数	629145 个个®×a	

a. °ò¤_¤u§@ªÅ间内¦sªº¥i¥Î©Ê	


¬Û关©Ê	
	­°温´T«×	¤£³z¤ô­±DIVISION	
´µ¥Ö尔°Ò Rho	­°温´T«×	¬Û关¨t数	1.000	-.005	
		显µÛ©Ê¡]双§À¡^	.	.976	
		个®×数	42	42	
	¤£³z¤ô­±DIVISION	¬Û关¨t数	-.005	1.000	
		显µÛ©Ê¡]双§À¡^	.976	.	
		个®×数	42	42	

NONPAR CORR
  /VARIABLES=­°温´T«× 绿¦aDIVISION
  /PRINT=SPEARMAN TWOTAIL NOSIG
  /MISSING=PAIRWISE.


«D参数¬Û关©Ê


备ª`	
¤w创«Ø输¥X	23-OCT-2023 21:18:32	
ª`释		
输¤J	数Õu	D:\ACCESSIBILITY AND PCI\SPSS\SPSS¥X图\无标题6.sav	
	¬¡动数Õu¶°	数Õu¶°1	
	过滤¾¹	<无>	
	权­«	<无>	
	©î¤À¤å¥ó	<无>	
	¤u§@数Õu¤å¥ó¤¤ªº¦æ数	45	
¯Ê¥¢­È处²z	对¯Ê¥¢ªº©w义	将¥Î户©w义ªº¯Ê¥¢­È视为¯Ê¥¢¡C	
	¨Ï¥Îªº个®×数	¨C对变¶qªº统计³£°ò¤_©Ò¦³对¤_该对变¶q¨ã¦³¦³®Ä数Õuªº个®×¡C	
语ªk	NONPAR CORR
  /VARIABLES=­°温´T«× 绿¦aDIVISION
  /PRINT=SPEARMAN TWOTAIL NOSIG
  /MISSING=PAIRWISE.	
资·½	处²zµ{§Ç时间	00:00:00.02	
	¯Ó¥Î时间	00:00:00.00	
	¤¹许ªº个®×数	629145 个个®×a	

a. °ò¤_¤u§@ªÅ间内¦sªº¥i¥Î©Ê	


¬Û关©Ê	
	­°温´T«×	绿¦aDIVISION	
´µ¥Ö尔°Ò Rho	­°温´T«×	¬Û关¨t数	1.000	.180	
		显µÛ©Ê¡]双§À¡^	.	.255	
		个®×数	42	42	
	绿¦aDIVISION	¬Û关¨t数	.180	1.000	
		显µÛ©Ê¡]双§À¡^	.255	.	
		个®×数	42	42	

NONPAR CORR
  /VARIABLES=­°温´T«× ¤ôÊ^DIVISION
  /PRINT=SPEARMAN TWOTAIL NOSIG
  /MISSING=PAIRWISE.


«D参数¬Û关©Ê


备ª`	
¤w创«Ø输¥X	23-OCT-2023 21:18:43	
ª`释		
输¤J	数Õu	D:\ACCESSIBILITY AND PCI\SPSS\SPSS¥X图\无标题6.sav	
	¬¡动数Õu¶°	数Õu¶°1	
	过滤¾¹	<无>	
	权­«	<无>	
	©î¤À¤å¥ó	<无>	
	¤u§@数Õu¤å¥ó¤¤ªº¦æ数	45	
¯Ê¥¢­È处²z	对¯Ê¥¢ªº©w义	将¥Î户©w义ªº¯Ê¥¢­È视为¯Ê¥¢¡C	
	¨Ï¥Îªº个®×数	¨C对变¶qªº统计³£°ò¤_©Ò¦³对¤_该对变¶q¨ã¦³¦³®Ä数Õuªº个®×¡C	
语ªk	NONPAR CORR
  /VARIABLES=­°温´T«× ¤ôÊ^DIVISION
  /PRINT=SPEARMAN TWOTAIL NOSIG
  /MISSING=PAIRWISE.	
资·½	处²zµ{§Ç时间	00:00:00.02	
	¯Ó¥Î时间	00:00:00.00	
	¤¹许ªº个®×数	629145 个个®×a	

a. °ò¤_¤u§@ªÅ间内¦sªº¥i¥Î©Ê	


¬Û关©Ê	
	­°温´T«×	¤ôÊ^DIVISION	
´µ¥Ö尔°Ò Rho	­°温´T«×	¬Û关¨t数	1.000	-.264	
		显µÛ©Ê¡]双§À¡^	.	.091	
		个®×数	42	42	
	¤ôÊ^DIVISION	¬Û关¨t数	-.264	1.000	
		显µÛ©Ê¡]双§À¡^	.091	.	
		个®×数	42	42	

NONPAR CORR
  /VARIABLES=­°温´T«× ¤£³z¤ô­±SPLIT
  /PRINT=SPEARMAN TWOTAIL NOSIG
  /MISSING=PAIRWISE.


«D参数¬Û关©Ê


备ª`	
¤w创«Ø输¥X	23-OCT-2023 21:18:55	
ª`释		
输¤J	数Õu	D:\ACCESSIBILITY AND PCI\SPSS\SPSS¥X图\无标题6.sav	
	¬¡动数Õu¶°	数Õu¶°1	
	过滤¾¹	<无>	
	权­«	<无>	
	©î¤À¤å¥ó	<无>	
	¤u§@数Õu¤å¥ó¤¤ªº¦æ数	45	
¯Ê¥¢­È处²z	对¯Ê¥¢ªº©w义	将¥Î户©w义ªº¯Ê¥¢­È视为¯Ê¥¢¡C	
	¨Ï¥Îªº个®×数	¨C对变¶qªº统计³£°ò¤_©Ò¦³对¤_该对变¶q¨ã¦³¦³®Ä数Õuªº个®×¡C	
语ªk	NONPAR CORR
  /VARIABLES=­°温´T«× ¤£³z¤ô­±SPLIT
  /PRINT=SPEARMAN TWOTAIL NOSIG
  /MISSING=PAIRWISE.	
资·½	处²zµ{§Ç时间	00:00:00.00	
	¯Ó¥Î时间	00:00:00.00	
	¤¹许ªº个®×数	629145 个个®×a	

a. °ò¤_¤u§@ªÅ间内¦sªº¥i¥Î©Ê	


¬Û关©Ê	
	­°温´T«×	¤£³z¤ô­±SPLIT	
´µ¥Ö尔°Ò Rho	­°温´T«×	¬Û关¨t数	1.000	-.024	
		显µÛ©Ê¡]双§À¡^	.	.881	
		个®×数	42	42	
	¤£³z¤ô­±SPLIT	¬Û关¨t数	-.024	1.000	
		显µÛ©Ê¡]双§À¡^	.881	.	
		个®×数	42	42	

NONPAR CORR
  /VARIABLES=­°温´T«× 绿¦aSPLIT
  /PRINT=SPEARMAN TWOTAIL NOSIG
  /MISSING=PAIRWISE.


«D参数¬Û关©Ê


备ª`	
¤w创«Ø输¥X	23-OCT-2023 21:19:07	
ª`释		
输¤J	数Õu	D:\ACCESSIBILITY AND PCI\SPSS\SPSS¥X图\无标题6.sav	
	¬¡动数Õu¶°	数Õu¶°1	
	过滤¾¹	<无>	
	权­«	<无>	
	©î¤À¤å¥ó	<无>	
	¤u§@数Õu¤å¥ó¤¤ªº¦æ数	45	
¯Ê¥¢­È处²z	对¯Ê¥¢ªº©w义	将¥Î户©w义ªº¯Ê¥¢­È视为¯Ê¥¢¡C	
	¨Ï¥Îªº个®×数	¨C对变¶qªº统计³£°ò¤_©Ò¦³对¤_该对变¶q¨ã¦³¦³®Ä数Õuªº个®×¡C	
语ªk	NONPAR CORR
  /VARIABLES=­°温´T«× 绿¦aSPLIT
  /PRINT=SPEARMAN TWOTAIL NOSIG
  /MISSING=PAIRWISE.	
资·½	处²zµ{§Ç时间	00:00:00.00	
	¯Ó¥Î时间	00:00:00.00	
	¤¹许ªº个®×数	629145 个个®×a	

a. °ò¤_¤u§@ªÅ间内¦sªº¥i¥Î©Ê	


¬Û关©Ê	
	­°温´T«×	绿¦aSPLIT	
´µ¥Ö尔°Ò Rho	­°温´T«×	¬Û关¨t数	1.000	.179	
		显µÛ©Ê¡]双§À¡^	.	.256	
		个®×数	42	42	
	绿¦aSPLIT	¬Û关¨t数	.179	1.000	
		显µÛ©Ê¡]双§À¡^	.256	.	
		个®×数	42	42	

NONPAR CORR
  /VARIABLES=­°温´T«× ¤ôÊ^SPLIT
  /PRINT=SPEARMAN TWOTAIL NOSIG
  /MISSING=PAIRWISE.


«D参数¬Û关©Ê


备ª`	
¤w创«Ø输¥X	23-OCT-2023 21:19:17	
ª`释		
输¤J	数Õu	D:\ACCESSIBILITY AND PCI\SPSS\SPSS¥X图\无标题6.sav	
	¬¡动数Õu¶°	数Õu¶°1	
	过滤¾¹	<无>	
	权­«	<无>	
	©î¤À¤å¥ó	<无>	
	¤u§@数Õu¤å¥ó¤¤ªº¦æ数	45	
¯Ê¥¢­È处²z	对¯Ê¥¢ªº©w义	将¥Î户©w义ªº¯Ê¥¢­È视为¯Ê¥¢¡C	
	¨Ï¥Îªº个®×数	¨C对变¶qªº统计³£°ò¤_©Ò¦³对¤_该对变¶q¨ã¦³¦³®Ä数Õuªº个®×¡C	
语ªk	NONPAR CORR
  /VARIABLES=­°温´T«× ¤ôÊ^SPLIT
  /PRINT=SPEARMAN TWOTAIL NOSIG
  /MISSING=PAIRWISE.	
资·½	处²zµ{§Ç时间	00:00:00.00	
	¯Ó¥Î时间	00:00:00.00	
	¤¹许ªº个®×数	629145 个个®×a	

a. °ò¤_¤u§@ªÅ间内¦sªº¥i¥Î©Ê	


¬Û关©Ê	
	­°温´T«×	¤ôÊ^SPLIT	
´µ¥Ö尔°Ò Rho	­°温´T«×	¬Û关¨t数	1.000	-.224	
		显µÛ©Ê¡]双§À¡^	.	.153	
		个®×数	42	42	
	¤ôÊ^SPLIT	¬Û关¨t数	-.224	1.000	
		显µÛ©Ê¡]双§À¡^	.153	.	
		个®×数	42	42	

NONPAR CORR
  /VARIABLES=­°温´T«× ¤£³z¤ô­±AI
  /PRINT=SPEARMAN TWOTAIL NOSIG
  /MISSING=PAIRWISE.


«D参数¬Û关©Ê


备ª`	
¤w创«Ø输¥X	23-OCT-2023 21:19:33	
ª`释		
输¤J	数Õu	D:\ACCESSIBILITY AND PCI\SPSS\SPSS¥X图\无标题6.sav	
	¬¡动数Õu¶°	数Õu¶°1	
	过滤¾¹	<无>	
	权­«	<无>	
	©î¤À¤å¥ó	<无>	
	¤u§@数Õu¤å¥ó¤¤ªº¦æ数	45	
¯Ê¥¢­È处²z	对¯Ê¥¢ªº©w义	将¥Î户©w义ªº¯Ê¥¢­È视为¯Ê¥¢¡C	
	¨Ï¥Îªº个®×数	¨C对变¶qªº统计³£°ò¤_©Ò¦³对¤_该对变¶q¨ã¦³¦³®Ä数Õuªº个®×¡C	
语ªk	NONPAR CORR
  /VARIABLES=­°温´T«× ¤£³z¤ô­±AI
  /PRINT=SPEARMAN TWOTAIL NOSIG
  /MISSING=PAIRWISE.	
资·½	处²zµ{§Ç时间	00:00:00.00	
	¯Ó¥Î时间	00:00:00.00	
	¤¹许ªº个®×数	629145 个个®×a	

a. °ò¤_¤u§@ªÅ间内¦sªº¥i¥Î©Ê	


¬Û关©Ê	
	­°温´T«×	¤£³z¤ô­±AI	
´µ¥Ö尔°Ò Rho	­°温´T«×	¬Û关¨t数	1.000	.166	
		显µÛ©Ê¡]双§À¡^	.	.293	
		个®×数	42	42	
	¤£³z¤ô­±AI	¬Û关¨t数	.166	1.000	
		显µÛ©Ê¡]双§À¡^	.293	.	
		个®×数	42	42	

NONPAR CORR
  /VARIABLES=­°温´T«× 绿¦aAI
  /PRINT=SPEARMAN TWOTAIL NOSIG
  /MISSING=PAIRWISE.


«D参数¬Û关©Ê


备ª`	
¤w创«Ø输¥X	23-OCT-2023 21:19:44	
ª`释		
输¤J	数Õu	D:\ACCESSIBILITY AND PCI\SPSS\SPSS¥X图\无标题6.sav	
	¬¡动数Õu¶°	数Õu¶°1	
	过滤¾¹	<无>	
	权­«	<无>	
	©î¤À¤å¥ó	<无>	
	¤u§@数Õu¤å¥ó¤¤ªº¦æ数	45	
¯Ê¥¢­È处²z	对¯Ê¥¢ªº©w义	将¥Î户©w义ªº¯Ê¥¢­È视为¯Ê¥¢¡C	
	¨Ï¥Îªº个®×数	¨C对变¶qªº统计³£°ò¤_©Ò¦³对¤_该对变¶q¨ã¦³¦³®Ä数Õuªº个®×¡C	
语ªk	NONPAR CORR
  /VARIABLES=­°温´T«× 绿¦aAI
  /PRINT=SPEARMAN TWOTAIL NOSIG
  /MISSING=PAIRWISE.	
资·½	处²zµ{§Ç时间	00:00:00.00	
	¯Ó¥Î时间	00:00:00.00	
	¤¹许ªº个®×数	629145 个个®×a	

a. °ò¤_¤u§@ªÅ间内¦sªº¥i¥Î©Ê	


¬Û关©Ê	
	­°温´T«×	绿¦aAI	
´µ¥Ö尔°Ò Rho	­°温´T«×	¬Û关¨t数	1.000	-.170	
		显µÛ©Ê¡]双§À¡^	.	.282	
		个®×数	42	42	
	绿¦aAI	¬Û关¨t数	-.170	1.000	
		显µÛ©Ê¡]双§À¡^	.282	.	
		个®×数	42	42	

NONPAR CORR
  /VARIABLES=­°温´T«× ¤ôÊ^AI
  /PRINT=SPEARMAN TWOTAIL NOSIG
  /MISSING=PAIRWISE.


«D参数¬Û关©Ê


备ª`	
¤w创«Ø输¥X	23-OCT-2023 21:19:57	
ª`释		
输¤J	数Õu	D:\ACCESSIBILITY AND PCI\SPSS\SPSS¥X图\无标题6.sav	
	¬¡动数Õu¶°	数Õu¶°1	
	过滤¾¹	<无>	
	权­«	<无>	
	©î¤À¤å¥ó	<无>	
	¤u§@数Õu¤å¥ó¤¤ªº¦æ数	45	
¯Ê¥¢­È处²z	对¯Ê¥¢ªº©w义	将¥Î户©w义ªº¯Ê¥¢­È视为¯Ê¥¢¡C	
	¨Ï¥Îªº个®×数	¨C对变¶qªº统计³£°ò¤_©Ò¦³对¤_该对变¶q¨ã¦³¦³®Ä数Õuªº个®×¡C	
语ªk	NONPAR CORR
  /VARIABLES=­°温´T«× ¤ôÊ^AI
  /PRINT=SPEARMAN TWOTAIL NOSIG
  /MISSING=PAIRWISE.	
资·½	处²zµ{§Ç时间	00:00:00.00	
	¯Ó¥Î时间	00:00:00.00	
	¤¹许ªº个®×数	629145 个个®×a	

a. °ò¤_¤u§@ªÅ间内¦sªº¥i¥Î©Ê	


¬Û关©Ê	
	­°温´T«×	¤ôÊ^AI	
´µ¥Ö尔°Ò Rho	­°温´T«×	¬Û关¨t数	1.000	.149	
		显µÛ©Ê¡]双§À¡^	.	.347	
		个®×数	42	42	
	¤ôÊ^AI	¬Û关¨t数	.149	1.000	
		显µÛ©Ê¡]双§À¡^	.347	.	
		个®×数	42	42	

NONPAR CORR
  /VARIABLES=­°温±è«× ¤£³z¤ô­±PD
  /PRINT=SPEARMAN TWOTAIL NOSIG
  /MISSING=PAIRWISE.


«D参数¬Û关©Ê


备ª`	
¤w创«Ø输¥X	23-OCT-2023 21:20:31	
ª`释		
输¤J	数Õu	D:\ACCESSIBILITY AND PCI\SPSS\SPSS¥X图\无标题6.sav	
	¬¡动数Õu¶°	数Õu¶°1	
	过滤¾¹	<无>	
	权­«	<无>	
	©î¤À¤å¥ó	<无>	
	¤u§@数Õu¤å¥ó¤¤ªº¦æ数	45	
¯Ê¥¢­È处²z	对¯Ê¥¢ªº©w义	将¥Î户©w义ªº¯Ê¥¢­È视为¯Ê¥¢¡C	
	¨Ï¥Îªº个®×数	¨C对变¶qªº统计³£°ò¤_©Ò¦³对¤_该对变¶q¨ã¦³¦³®Ä数Õuªº个®×¡C	
语ªk	NONPAR CORR
  /VARIABLES=­°温±è«× ¤£³z¤ô­±PD
  /PRINT=SPEARMAN TWOTAIL NOSIG
  /MISSING=PAIRWISE.	
资·½	处²zµ{§Ç时间	00:00:00.02	
	¯Ó¥Î时间	00:00:00.01	
	¤¹许ªº个®×数	629145 个个®×a	

a. °ò¤_¤u§@ªÅ间内¦sªº¥i¥Î©Ê	


¬Û关©Ê	
	­°温±è«×	¤£³z¤ô­±PD	
´µ¥Ö尔°Ò Rho	­°温±è«×	¬Û关¨t数	1.000	.073	
		显µÛ©Ê¡]双§À¡^	.	.648	
		个®×数	42	42	
	¤£³z¤ô­±PD	¬Û关¨t数	.073	1.000	
		显µÛ©Ê¡]双§À¡^	.648	.	
		个®×数	42	42	

NONPAR CORR
  /VARIABLES=­°温±è«× 绿¦aPD
  /PRINT=SPEARMAN TWOTAIL NOSIG
  /MISSING=PAIRWISE.


«D参数¬Û关©Ê


备ª`	
¤w创«Ø输¥X	23-OCT-2023 21:20:53	
ª`释		
输¤J	数Õu	D:\ACCESSIBILITY AND PCI\SPSS\SPSS¥X图\无标题6.sav	
	¬¡动数Õu¶°	数Õu¶°1	
	过滤¾¹	<无>	
	权­«	<无>	
	©î¤À¤å¥ó	<无>	
	¤u§@数Õu¤å¥ó¤¤ªº¦æ数	45	
¯Ê¥¢­È处²z	对¯Ê¥¢ªº©w义	将¥Î户©w义ªº¯Ê¥¢­È视为¯Ê¥¢¡C	
	¨Ï¥Îªº个®×数	¨C对变¶qªº统计³£°ò¤_©Ò¦³对¤_该对变¶q¨ã¦³¦³®Ä数Õuªº个®×¡C	
语ªk	NONPAR CORR
  /VARIABLES=­°温±è«× 绿¦aPD
  /PRINT=SPEARMAN TWOTAIL NOSIG
  /MISSING=PAIRWISE.	
资·½	处²zµ{§Ç时间	00:00:00.00	
	¯Ó¥Î时间	00:00:00.01	
	¤¹许ªº个®×数	629145 个个®×a	

a. °ò¤_¤u§@ªÅ间内¦sªº¥i¥Î©Ê	


¬Û关©Ê	
	­°温±è«×	绿¦aPD	
´µ¥Ö尔°Ò Rho	­°温±è«×	¬Û关¨t数	1.000	.153	
		显µÛ©Ê¡]双§À¡^	.	.333	
		个®×数	42	42	
	绿¦aPD	¬Û关¨t数	.153	1.000	
		显µÛ©Ê¡]双§À¡^	.333	.	
		个®×数	42	42	

NONPAR CORR
  /VARIABLES=­°温±è«× ¤ôÊ^PD
  /PRINT=SPEARMAN TWOTAIL NOSIG
  /MISSING=PAIRWISE.


«D参数¬Û关©Ê


备ª`	
¤w创«Ø输¥X	23-OCT-2023 21:21:04	
ª`释		
输¤J	数Õu	D:\ACCESSIBILITY AND PCI\SPSS\SPSS¥X图\无标题6.sav	
	¬¡动数Õu¶°	数Õu¶°1	
	过滤¾¹	<无>	
	权­«	<无>	
	©î¤À¤å¥ó	<无>	
	¤u§@数Õu¤å¥ó¤¤ªº¦æ数	45	
¯Ê¥¢­È处²z	对¯Ê¥¢ªº©w义	将¥Î户©w义ªº¯Ê¥¢­È视为¯Ê¥¢¡C	
	¨Ï¥Îªº个®×数	¨C对变¶qªº统计³£°ò¤_©Ò¦³对¤_该对变¶q¨ã¦³¦³®Ä数Õuªº个®×¡C	
语ªk	NONPAR CORR
  /VARIABLES=­°温±è«× ¤ôÊ^PD
  /PRINT=SPEARMAN TWOTAIL NOSIG
  /MISSING=PAIRWISE.	
资·½	处²zµ{§Ç时间	00:00:00.00	
	¯Ó¥Î时间	00:00:00.00	
	¤¹许ªº个®×数	629145 个个®×a	

a. °ò¤_¤u§@ªÅ间内¦sªº¥i¥Î©Ê	


¬Û关©Ê	
	­°温±è«×	¤ôÊ^PD	
´µ¥Ö尔°Ò Rho	­°温±è«×	¬Û关¨t数	1.000	-.081	
		显µÛ©Ê¡]双§À¡^	.	.611	
		个®×数	42	42	
	¤ôÊ^PD	¬Û关¨t数	-.081	1.000	
		显µÛ©Ê¡]双§À¡^	.611	.	
		个®×数	42	42	

NONPAR CORR
  /VARIABLES=­°温±è«× ¤£³z¤ô­±LPI
  /PRINT=SPEARMAN TWOTAIL NOSIG
  /MISSING=PAIRWISE.


«D参数¬Û关©Ê


备ª`	
¤w创«Ø输¥X	23-OCT-2023 21:21:14	
ª`释		
输¤J	数Õu	D:\ACCESSIBILITY AND PCI\SPSS\SPSS¥X图\无标题6.sav	
	¬¡动数Õu¶°	数Õu¶°1	
	过滤¾¹	<无>	
	权­«	<无>	
	©î¤À¤å¥ó	<无>	
	¤u§@数Õu¤å¥ó¤¤ªº¦æ数	45	
¯Ê¥¢­È处²z	对¯Ê¥¢ªº©w义	将¥Î户©w义ªº¯Ê¥¢­È视为¯Ê¥¢¡C	
	¨Ï¥Îªº个®×数	¨C对变¶qªº统计³£°ò¤_©Ò¦³对¤_该对变¶q¨ã¦³¦³®Ä数Õuªº个®×¡C	
语ªk	NONPAR CORR
  /VARIABLES=­°温±è«× ¤£³z¤ô­±LPI
  /PRINT=SPEARMAN TWOTAIL NOSIG
  /MISSING=PAIRWISE.	
资·½	处²zµ{§Ç时间	00:00:00.02	
	¯Ó¥Î时间	00:00:00.00	
	¤¹许ªº个®×数	629145 个个®×a	

a. °ò¤_¤u§@ªÅ间内¦sªº¥i¥Î©Ê	


¬Û关©Ê	
	­°温±è«×	¤£³z¤ô­±LPI	
´µ¥Ö尔°Ò Rho	­°温±è«×	¬Û关¨t数	1.000	.146	
		显µÛ©Ê¡]双§À¡^	.	.355	
		个®×数	42	42	
	¤£³z¤ô­±LPI	¬Û关¨t数	.146	1.000	
		显µÛ©Ê¡]双§À¡^	.355	.	
		个®×数	42	42	

NONPAR CORR
  /VARIABLES=­°温±è«× 绿¦aLPI
  /PRINT=SPEARMAN TWOTAIL NOSIG
  /MISSING=PAIRWISE.


«D参数¬Û关©Ê


备ª`	
¤w创«Ø输¥X	23-OCT-2023 21:21:23	
ª`释		
输¤J	数Õu	D:\ACCESSIBILITY AND PCI\SPSS\SPSS¥X图\无标题6.sav	
	¬¡动数Õu¶°	数Õu¶°1	
	过滤¾¹	<无>	
	权­«	<无>	
	©î¤À¤å¥ó	<无>	
	¤u§@数Õu¤å¥ó¤¤ªº¦æ数	45	
¯Ê¥¢­È处²z	对¯Ê¥¢ªº©w义	将¥Î户©w义ªº¯Ê¥¢­È视为¯Ê¥¢¡C	
	¨Ï¥Îªº个®×数	¨C对变¶qªº统计³£°ò¤_©Ò¦³对¤_该对变¶q¨ã¦³¦³®Ä数Õuªº个®×¡C	
语ªk	NONPAR CORR
  /VARIABLES=­°温±è«× 绿¦aLPI
  /PRINT=SPEARMAN TWOTAIL NOSIG
  /MISSING=PAIRWISE.	
资·½	处²zµ{§Ç时间	00:00:00.00	
	¯Ó¥Î时间	00:00:00.00	
	¤¹许ªº个®×数	629145 个个®×a	

a. °ò¤_¤u§@ªÅ间内¦sªº¥i¥Î©Ê	


¬Û关©Ê	
	­°温±è«×	绿¦aLPI	
´µ¥Ö尔°Ò Rho	­°温±è«×	¬Û关¨t数	1.000	.014	
		显µÛ©Ê¡]双§À¡^	.	.929	
		个®×数	42	42	
	绿¦aLPI	¬Û关¨t数	.014	1.000	
		显µÛ©Ê¡]双§À¡^	.929	.	
		个®×数	42	42	

NONPAR CORR
  /VARIABLES=­°温±è«× ¤ôÊ^LPI
  /PRINT=SPEARMAN TWOTAIL NOSIG
  /MISSING=PAIRWISE.


«D参数¬Û关©Ê


备ª`	
¤w创«Ø输¥X	23-OCT-2023 21:21:35	
ª`释		
输¤J	数Õu	D:\ACCESSIBILITY AND PCI\SPSS\SPSS¥X图\无标题6.sav	
	¬¡动数Õu¶°	数Õu¶°1	
	过滤¾¹	<无>	
	权­«	<无>	
	©î¤À¤å¥ó	<无>	
	¤u§@数Õu¤å¥ó¤¤ªº¦æ数	45	
¯Ê¥¢­È处²z	对¯Ê¥¢ªº©w义	将¥Î户©w义ªº¯Ê¥¢­È视为¯Ê¥¢¡C	
	¨Ï¥Îªº个®×数	¨C对变¶qªº统计³£°ò¤_©Ò¦³对¤_该对变¶q¨ã¦³¦³®Ä数Õuªº个®×¡C	
语ªk	NONPAR CORR
  /VARIABLES=­°温±è«× ¤ôÊ^LPI
  /PRINT=SPEARMAN TWOTAIL NOSIG
  /MISSING=PAIRWISE.	
资·½	处²zµ{§Ç时间	00:00:00.00	
	¯Ó¥Î时间	00:00:00.00	
	¤¹许ªº个®×数	629145 个个®×a	

a. °ò¤_¤u§@ªÅ间内¦sªº¥i¥Î©Ê	


¬Û关©Ê	
	­°温±è«×	¤ôÊ^LPI	
´µ¥Ö尔°Ò Rho	­°温±è«×	¬Û关¨t数	1.000	-.068	
		显µÛ©Ê¡]双§À¡^	.	.669	
		个®×数	42	42	
	¤ôÊ^LPI	¬Û关¨t数	-.068	1.000	
		显µÛ©Ê¡]双§À¡^	.669	.	
		个®×数	42	42	

NONPAR CORR
  /VARIABLES=­°温±è«× ¤£³z¤ô­±LSI
  /PRINT=SPEARMAN TWOTAIL NOSIG
  /MISSING=PAIRWISE.


«D参数¬Û关©Ê


备ª`	
¤w创«Ø输¥X	23-OCT-2023 21:21:47	
ª`释		
输¤J	数Õu	D:\ACCESSIBILITY AND PCI\SPSS\SPSS¥X图\无标题6.sav	
	¬¡动数Õu¶°	数Õu¶°1	
	过滤¾¹	<无>	
	权­«	<无>	
	©î¤À¤å¥ó	<无>	
	¤u§@数Õu¤å¥ó¤¤ªº¦æ数	45	
¯Ê¥¢­È处²z	对¯Ê¥¢ªº©w义	将¥Î户©w义ªº¯Ê¥¢­È视为¯Ê¥¢¡C	
	¨Ï¥Îªº个®×数	¨C对变¶qªº统计³£°ò¤_©Ò¦³对¤_该对变¶q¨ã¦³¦³®Ä数Õuªº个®×¡C	
语ªk	NONPAR CORR
  /VARIABLES=­°温±è«× ¤£³z¤ô­±LSI
  /PRINT=SPEARMAN TWOTAIL NOSIG
  /MISSING=PAIRWISE.	
资·½	处²zµ{§Ç时间	00:00:00.00	
	¯Ó¥Î时间	00:00:00.00	
	¤¹许ªº个®×数	629145 个个®×a	

a. °ò¤_¤u§@ªÅ间内¦sªº¥i¥Î©Ê	


¬Û关©Ê	
	­°温±è«×	¤£³z¤ô­±LSI	
´µ¥Ö尔°Ò Rho	­°温±è«×	¬Û关¨t数	1.000	-.302	
		显µÛ©Ê¡]双§À¡^	.	.052	
		个®×数	42	42	
	¤£³z¤ô­±LSI	¬Û关¨t数	-.302	1.000	
		显µÛ©Ê¡]双§À¡^	.052	.	
		个®×数	42	42	

NONPAR CORR
  /VARIABLES=­°温±è«× 绿¦aLSI
  /PRINT=SPEARMAN TWOTAIL NOSIG
  /MISSING=PAIRWISE.


«D参数¬Û关©Ê


备ª`	
¤w创«Ø输¥X	23-OCT-2023 21:21:57	
ª`释		
输¤J	数Õu	D:\ACCESSIBILITY AND PCI\SPSS\SPSS¥X图\无标题6.sav	
	¬¡动数Õu¶°	数Õu¶°1	
	过滤¾¹	<无>	
	权­«	<无>	
	©î¤À¤å¥ó	<无>	
	¤u§@数Õu¤å¥ó¤¤ªº¦æ数	45	
¯Ê¥¢­È处²z	对¯Ê¥¢ªº©w义	将¥Î户©w义ªº¯Ê¥¢­È视为¯Ê¥¢¡C	
	¨Ï¥Îªº个®×数	¨C对变¶qªº统计³£°ò¤_©Ò¦³对¤_该对变¶q¨ã¦³¦³®Ä数Õuªº个®×¡C	
语ªk	NONPAR CORR
  /VARIABLES=­°温±è«× 绿¦aLSI
  /PRINT=SPEARMAN TWOTAIL NOSIG
  /MISSING=PAIRWISE.	
资·½	处²zµ{§Ç时间	00:00:00.02	
	¯Ó¥Î时间	00:00:00.00	
	¤¹许ªº个®×数	629145 个个®×a	

a. °ò¤_¤u§@ªÅ间内¦sªº¥i¥Î©Ê	


¬Û关©Ê	
	­°温±è«×	绿¦aLSI	
´µ¥Ö尔°Ò Rho	­°温±è«×	¬Û关¨t数	1.000	-.213	
		显µÛ©Ê¡]双§À¡^	.	.177	
		个®×数	42	42	
	绿¦aLSI	¬Û关¨t数	-.213	1.000	
		显µÛ©Ê¡]双§À¡^	.177	.	
		个®×数	42	42	

NONPAR CORR
  /VARIABLES=­°温±è«× ¤ôÊ^LSI
  /PRINT=SPEARMAN TWOTAIL NOSIG
  /MISSING=PAIRWISE.


«D参数¬Û关©Ê


备ª`	
¤w创«Ø输¥X	23-OCT-2023 21:22:08	
ª`释		
输¤J	数Õu	D:\ACCESSIBILITY AND PCI\SPSS\SPSS¥X图\无标题6.sav	
	¬¡动数Õu¶°	数Õu¶°1	
	过滤¾¹	<无>	
	权­«	<无>	
	©î¤À¤å¥ó	<无>	
	¤u§@数Õu¤å¥ó¤¤ªº¦æ数	45	
¯Ê¥¢­È处²z	对¯Ê¥¢ªº©w义	将¥Î户©w义ªº¯Ê¥¢­È视为¯Ê¥¢¡C	
	¨Ï¥Îªº个®×数	¨C对变¶qªº统计³£°ò¤_©Ò¦³对¤_该对变¶q¨ã¦³¦³®Ä数Õuªº个®×¡C	
语ªk	NONPAR CORR
  /VARIABLES=­°温±è«× ¤ôÊ^LSI
  /PRINT=SPEARMAN TWOTAIL NOSIG
  /MISSING=PAIRWISE.	
资·½	处²zµ{§Ç时间	00:00:00.00	
	¯Ó¥Î时间	00:00:00.00	
	¤¹许ªº个®×数	629145 个个®×a	

a. °ò¤_¤u§@ªÅ间内¦sªº¥i¥Î©Ê	


¬Û关©Ê	
	­°温±è«×	¤ôÊ^LSI	
´µ¥Ö尔°Ò Rho	­°温±è«×	¬Û关¨t数	1.000	-.265	
		显µÛ©Ê¡]双§À¡^	.	.090	
		个®×数	42	42	
	¤ôÊ^LSI	¬Û关¨t数	-.265	1.000	
		显µÛ©Ê¡]双§À¡^	.090	.	
		个®×数	42	42	

NONPAR CORR
  /VARIABLES=­°温±è«× ¤£³z¤ô­±DIVISION
  /PRINT=SPEARMAN TWOTAIL NOSIG
  /MISSING=PAIRWISE.


«D参数¬Û关©Ê


备ª`	
¤w创«Ø输¥X	23-OCT-2023 21:22:24	
ª`释		
输¤J	数Õu	D:\ACCESSIBILITY AND PCI\SPSS\SPSS¥X图\无标题6.sav	
	¬¡动数Õu¶°	数Õu¶°1	
	过滤¾¹	<无>	
	权­«	<无>	
	©î¤À¤å¥ó	<无>	
	¤u§@数Õu¤å¥ó¤¤ªº¦æ数	45	
¯Ê¥¢­È处²z	对¯Ê¥¢ªº©w义	将¥Î户©w义ªº¯Ê¥¢­È视为¯Ê¥¢¡C	
	¨Ï¥Îªº个®×数	¨C对变¶qªº统计³£°ò¤_©Ò¦³对¤_该对变¶q¨ã¦³¦³®Ä数Õuªº个®×¡C	
语ªk	NONPAR CORR
  /VARIABLES=­°温±è«× ¤£³z¤ô­±DIVISION
  /PRINT=SPEARMAN TWOTAIL NOSIG
  /MISSING=PAIRWISE.	
资·½	处²zµ{§Ç时间	00:00:00.02	
	¯Ó¥Î时间	00:00:00.01	
	¤¹许ªº个®×数	629145 个个®×a	

a. °ò¤_¤u§@ªÅ间内¦sªº¥i¥Î©Ê	


¬Û关©Ê	
	­°温±è«×	¤£³z¤ô­±DIVISION	
´µ¥Ö尔°Ò Rho	­°温±è«×	¬Û关¨t数	1.000	-.135	
		显µÛ©Ê¡]双§À¡^	.	.393	
		个®×数	42	42	
	¤£³z¤ô­±DIVISION	¬Û关¨t数	-.135	1.000	
		显µÛ©Ê¡]双§À¡^	.393	.	
		个®×数	42	42	

NONPAR CORR
  /VARIABLES=­°温±è«× 绿¦aDIVISION
  /PRINT=SPEARMAN TWOTAIL NOSIG
  /MISSING=PAIRWISE.


«D参数¬Û关©Ê


备ª`	
¤w创«Ø输¥X	23-OCT-2023 21:22:36	
ª`释		
输¤J	数Õu	D:\ACCESSIBILITY AND PCI\SPSS\SPSS¥X图\无标题6.sav	
	¬¡动数Õu¶°	数Õu¶°1	
	过滤¾¹	<无>	
	权­«	<无>	
	©î¤À¤å¥ó	<无>	
	¤u§@数Õu¤å¥ó¤¤ªº¦æ数	45	
¯Ê¥¢­È处²z	对¯Ê¥¢ªº©w义	将¥Î户©w义ªº¯Ê¥¢­È视为¯Ê¥¢¡C	
	¨Ï¥Îªº个®×数	¨C对变¶qªº统计³£°ò¤_©Ò¦³对¤_该对变¶q¨ã¦³¦³®Ä数Õuªº个®×¡C	
语ªk	NONPAR CORR
  /VARIABLES=­°温±è«× 绿¦aDIVISION
  /PRINT=SPEARMAN TWOTAIL NOSIG
  /MISSING=PAIRWISE.	
资·½	处²zµ{§Ç时间	00:00:00.00	
	¯Ó¥Î时间	00:00:00.00	
	¤¹许ªº个®×数	629145 个个®×a	

a. °ò¤_¤u§@ªÅ间内¦sªº¥i¥Î©Ê	


¬Û关©Ê	
	­°温±è«×	绿¦aDIVISION	
´µ¥Ö尔°Ò Rho	­°温±è«×	¬Û关¨t数	1.000	-.013	
		显µÛ©Ê¡]双§À¡^	.	.936	
		个®×数	42	42	
	绿¦aDIVISION	¬Û关¨t数	-.013	1.000	
		显µÛ©Ê¡]双§À¡^	.936	.	
		个®×数	42	42	

NONPAR CORR
  /VARIABLES=­°温±è«× ¤ôÊ^DIVISION
  /PRINT=SPEARMAN TWOTAIL NOSIG
  /MISSING=PAIRWISE.


«D参数¬Û关©Ê


备ª`	
¤w创«Ø输¥X	23-OCT-2023 21:22:56	
ª`释		
输¤J	数Õu	D:\ACCESSIBILITY AND PCI\SPSS\SPSS¥X图\无标题6.sav	
	¬¡动数Õu¶°	数Õu¶°1	
	过滤¾¹	<无>	
	权­«	<无>	
	©î¤À¤å¥ó	<无>	
	¤u§@数Õu¤å¥ó¤¤ªº¦æ数	45	
¯Ê¥¢­È处²z	对¯Ê¥¢ªº©w义	将¥Î户©w义ªº¯Ê¥¢­È视为¯Ê¥¢¡C	
	¨Ï¥Îªº个®×数	¨C对变¶qªº统计³£°ò¤_©Ò¦³对¤_该对变¶q¨ã¦³¦³®Ä数Õuªº个®×¡C	
语ªk	NONPAR CORR
  /VARIABLES=­°温±è«× ¤ôÊ^DIVISION
  /PRINT=SPEARMAN TWOTAIL NOSIG
  /MISSING=PAIRWISE.	
资·½	处²zµ{§Ç时间	00:00:00.00	
	¯Ó¥Î时间	00:00:00.00	
	¤¹许ªº个®×数	629145 个个®×a	

a. °ò¤_¤u§@ªÅ间内¦sªº¥i¥Î©Ê	


¬Û关©Ê	
	­°温±è«×	¤ôÊ^DIVISION	
´µ¥Ö尔°Ò Rho	­°温±è«×	¬Û关¨t数	1.000	-.150	
		显µÛ©Ê¡]双§À¡^	.	.342	
		个®×数	42	42	
	¤ôÊ^DIVISION	¬Û关¨t数	-.150	1.000	
		显µÛ©Ê¡]双§À¡^	.342	.	
		个®×数	42	42	

NONPAR CORR
  /VARIABLES=­°温±è«× ¤£³z¤ô­±SPLIT
  /PRINT=SPEARMAN TWOTAIL NOSIG
  /MISSING=PAIRWISE.


«D参数¬Û关©Ê


备ª`	
¤w创«Ø输¥X	23-OCT-2023 21:23:12	
ª`释		
输¤J	数Õu	D:\ACCESSIBILITY AND PCI\SPSS\SPSS¥X图\无标题6.sav	
	¬¡动数Õu¶°	数Õu¶°1	
	过滤¾¹	<无>	
	权­«	<无>	
	©î¤À¤å¥ó	<无>	
	¤u§@数Õu¤å¥ó¤¤ªº¦æ数	45	
¯Ê¥¢­È处²z	对¯Ê¥¢ªº©w义	将¥Î户©w义ªº¯Ê¥¢­È视为¯Ê¥¢¡C	
	¨Ï¥Îªº个®×数	¨C对变¶qªº统计³£°ò¤_©Ò¦³对¤_该对变¶q¨ã¦³¦³®Ä数Õuªº个®×¡C	
语ªk	NONPAR CORR
  /VARIABLES=­°温±è«× ¤£³z¤ô­±SPLIT
  /PRINT=SPEARMAN TWOTAIL NOSIG
  /MISSING=PAIRWISE.	
资·½	处²zµ{§Ç时间	00:00:00.02	
	¯Ó¥Î时间	00:00:00.00	
	¤¹许ªº个®×数	629145 个个®×a	

a. °ò¤_¤u§@ªÅ间内¦sªº¥i¥Î©Ê	


¬Û关©Ê	
	­°温±è«×	¤£³z¤ô­±SPLIT	
´µ¥Ö尔°Ò Rho	­°温±è«×	¬Û关¨t数	1.000	-.127	
		显µÛ©Ê¡]双§À¡^	.	.422	
		个®×数	42	42	
	¤£³z¤ô­±SPLIT	¬Û关¨t数	-.127	1.000	
		显µÛ©Ê¡]双§À¡^	.422	.	
		个®×数	42	42	

NONPAR CORR
  /VARIABLES=­°温±è«× 绿¦aSPLIT
  /PRINT=SPEARMAN TWOTAIL NOSIG
  /MISSING=PAIRWISE.


«D参数¬Û关©Ê


备ª`	
¤w创«Ø输¥X	23-OCT-2023 21:23:25	
ª`释		
输¤J	数Õu	D:\ACCESSIBILITY AND PCI\SPSS\SPSS¥X图\无标题6.sav	
	¬¡动数Õu¶°	数Õu¶°1	
	过滤¾¹	<无>	
	权­«	<无>	
	©î¤À¤å¥ó	<无>	
	¤u§@数Õu¤å¥ó¤¤ªº¦æ数	45	
¯Ê¥¢­È处²z	对¯Ê¥¢ªº©w义	将¥Î户©w义ªº¯Ê¥¢­È视为¯Ê¥¢¡C	
	¨Ï¥Îªº个®×数	¨C对变¶qªº统计³£°ò¤_©Ò¦³对¤_该对变¶q¨ã¦³¦³®Ä数Õuªº个®×¡C	
语ªk	NONPAR CORR
  /VARIABLES=­°温±è«× 绿¦aSPLIT
  /PRINT=SPEARMAN TWOTAIL NOSIG
  /MISSING=PAIRWISE.	
资·½	处²zµ{§Ç时间	00:00:00.00	
	¯Ó¥Î时间	00:00:00.00	
	¤¹许ªº个®×数	629145 个个®×a	

a. °ò¤_¤u§@ªÅ间内¦sªº¥i¥Î©Ê	


¬Û关©Ê	
	­°温±è«×	绿¦aSPLIT	
´µ¥Ö尔°Ò Rho	­°温±è«×	¬Û关¨t数	1.000	-.011	
		显µÛ©Ê¡]双§À¡^	.	.944	
		个®×数	42	42	
	绿¦aSPLIT	¬Û关¨t数	-.011	1.000	
		显µÛ©Ê¡]双§À¡^	.944	.	
		个®×数	42	42	

NONPAR CORR
  /VARIABLES=­°温±è«× ¤ôÊ^SPLIT
  /PRINT=SPEARMAN TWOTAIL NOSIG
  /MISSING=PAIRWISE.


«D参数¬Û关©Ê


备ª`	
¤w创«Ø输¥X	23-OCT-2023 21:23:35	
ª`释		
输¤J	数Õu	D:\ACCESSIBILITY AND PCI\SPSS\SPSS¥X图\无标题6.sav	
	¬¡动数Õu¶°	数Õu¶°1	
	过滤¾¹	<无>	
	权­«	<无>	
	©î¤À¤å¥ó	<无>	
	¤u§@数Õu¤å¥ó¤¤ªº¦æ数	45	
¯Ê¥¢­È处²z	对¯Ê¥¢ªº©w义	将¥Î户©w义ªº¯Ê¥¢­È视为¯Ê¥¢¡C	
	¨Ï¥Îªº个®×数	¨C对变¶qªº统计³£°ò¤_©Ò¦³对¤_该对变¶q¨ã¦³¦³®Ä数Õuªº个®×¡C	
语ªk	NONPAR CORR
  /VARIABLES=­°温±è«× ¤ôÊ^SPLIT
  /PRINT=SPEARMAN TWOTAIL NOSIG
  /MISSING=PAIRWISE.	
资·½	处²zµ{§Ç时间	00:00:00.00	
	¯Ó¥Î时间	00:00:00.00	
	¤¹许ªº个®×数	629145 个个®×a	

a. °ò¤_¤u§@ªÅ间内¦sªº¥i¥Î©Ê	


¬Û关©Ê	
	­°温±è«×	¤ôÊ^SPLIT	
´µ¥Ö尔°Ò Rho	­°温±è«×	¬Û关¨t数	1.000	-.104	
		显µÛ©Ê¡]双§À¡^	.	.512	
		个®×数	42	42	
	¤ôÊ^SPLIT	¬Û关¨t数	-.104	1.000	
		显µÛ©Ê¡]双§À¡^	.512	.	
		个®×数	42	42	

NONPAR CORR
  /VARIABLES=­°温±è«× ¤£³z¤ô­±AI
  /PRINT=SPEARMAN TWOTAIL NOSIG
  /MISSING=PAIRWISE.


«D参数¬Û关©Ê


备ª`	
¤w创«Ø输¥X	23-OCT-2023 21:23:47	
ª`释		
输¤J	数Õu	D:\ACCESSIBILITY AND PCI\SPSS\SPSS¥X图\无标题6.sav	
	¬¡动数Õu¶°	数Õu¶°1	
	过滤¾¹	<无>	
	权­«	<无>	
	©î¤À¤å¥ó	<无>	
	¤u§@数Õu¤å¥ó¤¤ªº¦æ数	45	
¯Ê¥¢­È处²z	对¯Ê¥¢ªº©w义	将¥Î户©w义ªº¯Ê¥¢­È视为¯Ê¥¢¡C	
	¨Ï¥Îªº个®×数	¨C对变¶qªº统计³£°ò¤_©Ò¦³对¤_该对变¶q¨ã¦³¦³®Ä数Õuªº个®×¡C	
语ªk	NONPAR CORR
  /VARIABLES=­°温±è«× ¤£³z¤ô­±AI
  /PRINT=SPEARMAN TWOTAIL NOSIG
  /MISSING=PAIRWISE.	
资·½	处²zµ{§Ç时间	00:00:00.00	
	¯Ó¥Î时间	00:00:00.00	
	¤¹许ªº个®×数	629145 个个®×a	

a. °ò¤_¤u§@ªÅ间内¦sªº¥i¥Î©Ê	


¬Û关©Ê	
	­°温±è«×	¤£³z¤ô­±AI	
´µ¥Ö尔°Ò Rho	­°温±è«×	¬Û关¨t数	1.000	.067	
		显µÛ©Ê¡]双§À¡^	.	.676	
		个®×数	42	42	
	¤£³z¤ô­±AI	¬Û关¨t数	.067	1.000	
		显µÛ©Ê¡]双§À¡^	.676	.	
		个®×数	42	42	

NONPAR CORR
  /VARIABLES=­°温±è«× 绿¦aAI
  /PRINT=SPEARMAN TWOTAIL NOSIG
  /MISSING=PAIRWISE.


«D参数¬Û关©Ê


备ª`	
¤w创«Ø输¥X	23-OCT-2023 21:23:58	
ª`释		
输¤J	数Õu	D:\ACCESSIBILITY AND PCI\SPSS\SPSS¥X图\无标题6.sav	
	¬¡动数Õu¶°	数Õu¶°1	
	过滤¾¹	<无>	
	权­«	<无>	
	©î¤À¤å¥ó	<无>	
	¤u§@数Õu¤å¥ó¤¤ªº¦æ数	45	
¯Ê¥¢­È处²z	对¯Ê¥¢ªº©w义	将¥Î户©w义ªº¯Ê¥¢­È视为¯Ê¥¢¡C	
	¨Ï¥Îªº个®×数	¨C对变¶qªº统计³£°ò¤_©Ò¦³对¤_该对变¶q¨ã¦³¦³®Ä数Õuªº个®×¡C	
语ªk	NONPAR CORR
  /VARIABLES=­°温±è«× 绿¦aAI
  /PRINT=SPEARMAN TWOTAIL NOSIG
  /MISSING=PAIRWISE.	
资·½	处²zµ{§Ç时间	00:00:00.00	
	¯Ó¥Î时间	00:00:00.00	
	¤¹许ªº个®×数	629145 个个®×a	

a. °ò¤_¤u§@ªÅ间内¦sªº¥i¥Î©Ê	


¬Û关©Ê	
	­°温±è«×	绿¦aAI	
´µ¥Ö尔°Ò Rho	­°温±è«×	¬Û关¨t数	1.000	-.119	
		显µÛ©Ê¡]双§À¡^	.	.452	
		个®×数	42	42	
	绿¦aAI	¬Û关¨t数	-.119	1.000	
		显µÛ©Ê¡]双§À¡^	.452	.	
		个®×数	42	42	

NONPAR CORR
  /VARIABLES=­°温±è«× ¤ôÊ^AI
  /PRINT=SPEARMAN TWOTAIL NOSIG
  /MISSING=PAIRWISE.


«D参数¬Û关©Ê


备ª`	
¤w创«Ø输¥X	23-OCT-2023 21:24:11	
ª`释		
输¤J	数Õu	D:\ACCESSIBILITY AND PCI\SPSS\SPSS¥X图\无标题6.sav	
	¬¡动数Õu¶°	数Õu¶°1	
	过滤¾¹	<无>	
	权­«	<无>	
	©î¤À¤å¥ó	<无>	
	¤u§@数Õu¤å¥ó¤¤ªº¦æ数	45	
¯Ê¥¢­È处²z	对¯Ê¥¢ªº©w义	将¥Î户©w义ªº¯Ê¥¢­È视为¯Ê¥¢¡C	
	¨Ï¥Îªº个®×数	¨C对变¶qªº统计³£°ò¤_©Ò¦³对¤_该对变¶q¨ã¦³¦³®Ä数Õuªº个®×¡C	
语ªk	NONPAR CORR
  /VARIABLES=­°温±è«× ¤ôÊ^AI
  /PRINT=SPEARMAN TWOTAIL NOSIG
  /MISSING=PAIRWISE.	
资·½	处²zµ{§Ç时间	00:00:00.00	
	¯Ó¥Î时间	00:00:00.00	
	¤¹许ªº个®×数	629145 个个®×a	

a. °ò¤_¤u§@ªÅ间内¦sªº¥i¥Î©Ê	


¬Û关©Ê	
	­°温±è«×	¤ôÊ^AI	
´µ¥Ö尔°Ò Rho	­°温±è«×	¬Û关¨t数	1.000	-.085	
		显µÛ©Ê¡]双§À¡^	.	.590	
		个®×数	42	42	
	¤ôÊ^AI	¬Û关¨t数	-.085	1.000	
		显µÛ©Ê¡]双§À¡^	.590	.	
		个®×数	42	42	
