# Supplementary figures and images for: Optimizing spatial equity of urban park cooling services: Integrating landscape metrics with K-means and PSO algorithms in Nanchang, China
Source: PLoS One. 2026 Mar 19;21(3):e0344026. doi: 10.1371/journal.pone.0344026 (PMC13001981; doi:10.1371/journal.pone.0344026)

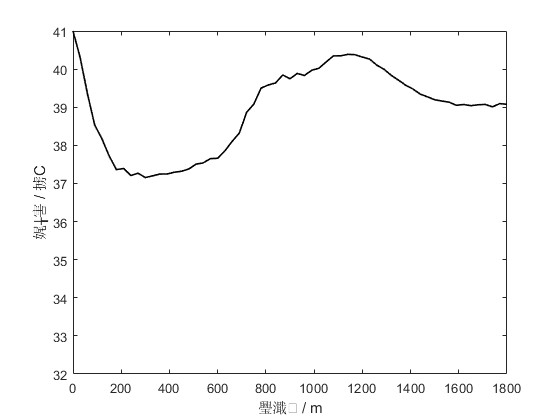

Supplement: S1 File — (ZIP) [file pone.0344026.s001.zip › Supplementary material/3 Matlab algorithms and some results/Park temperature inflection plot in Matlab/0ZJGC.jpg]

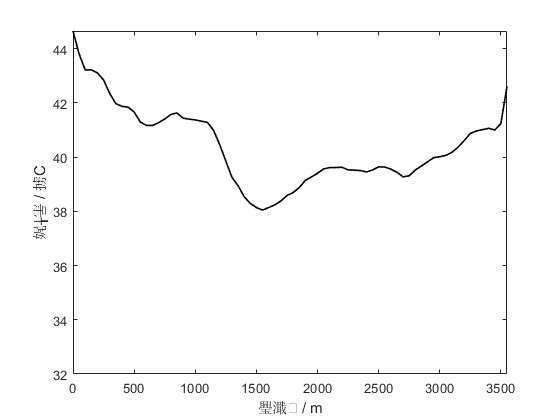

Supplement: S1 File — (ZIP) [file pone.0344026.s001.zip › Supplementary material/3 Matlab algorithms and some results/Park temperature inflection plot in Matlab/1.jpg]

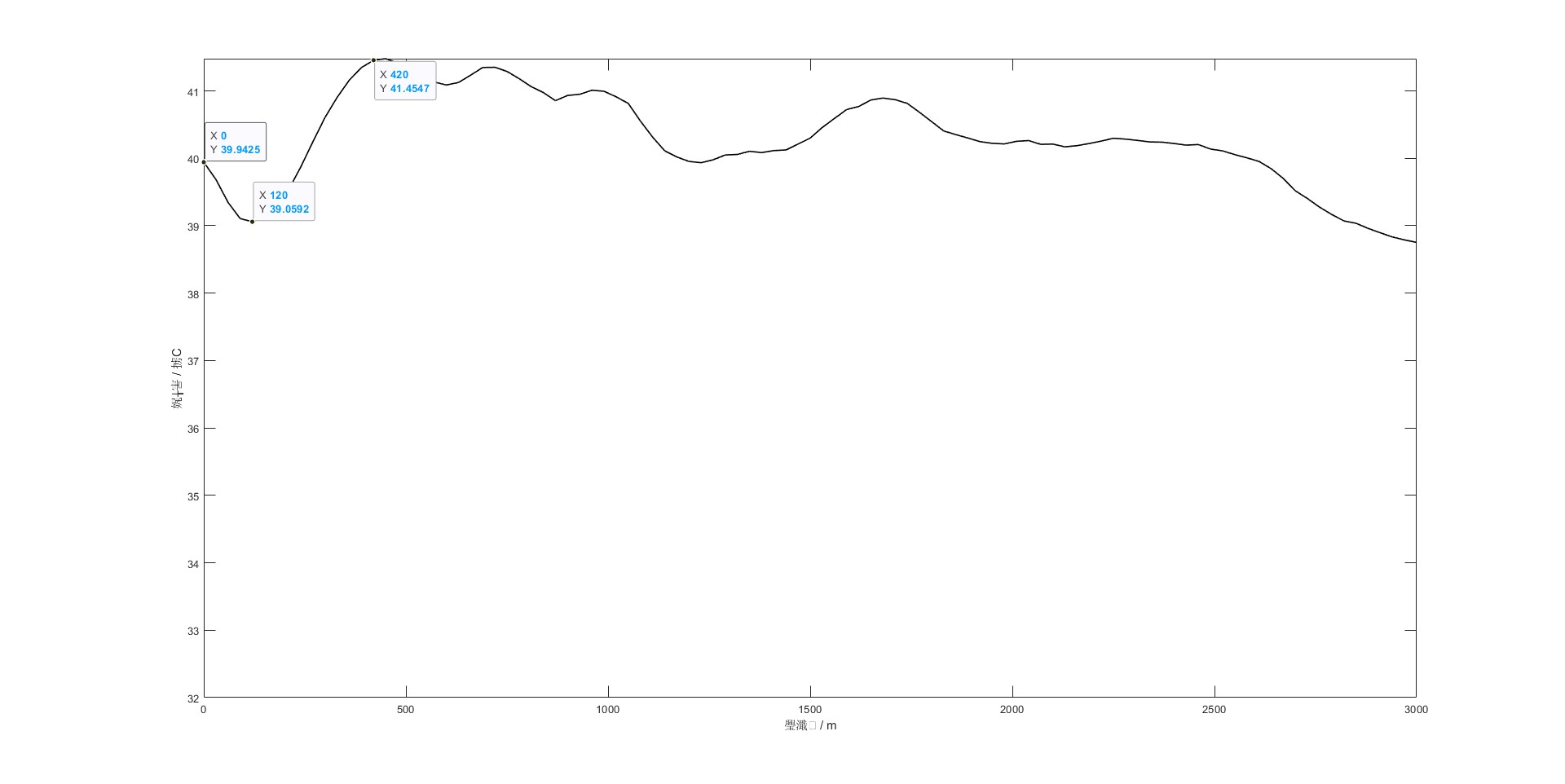

Supplement: S1 File — (ZIP) [file pone.0344026.s001.zip › Supplementary material/3 Matlab algorithms and some results/Park temperature inflection plot in Matlab/10.jpg]

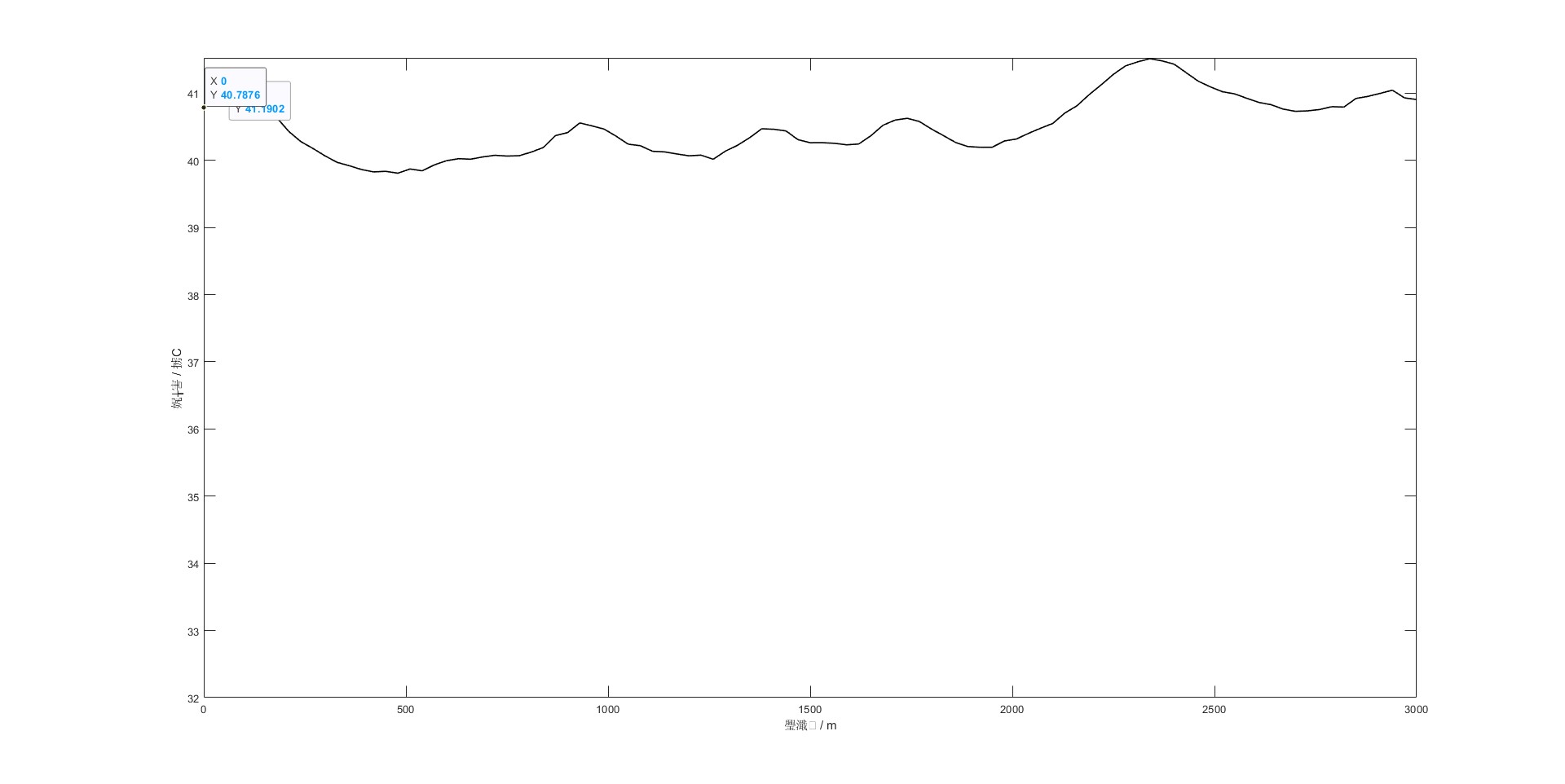

Supplement: S1 File — (ZIP) [file pone.0344026.s001.zip › Supplementary material/3 Matlab algorithms and some results/Park temperature inflection plot in Matlab/11.jpg]

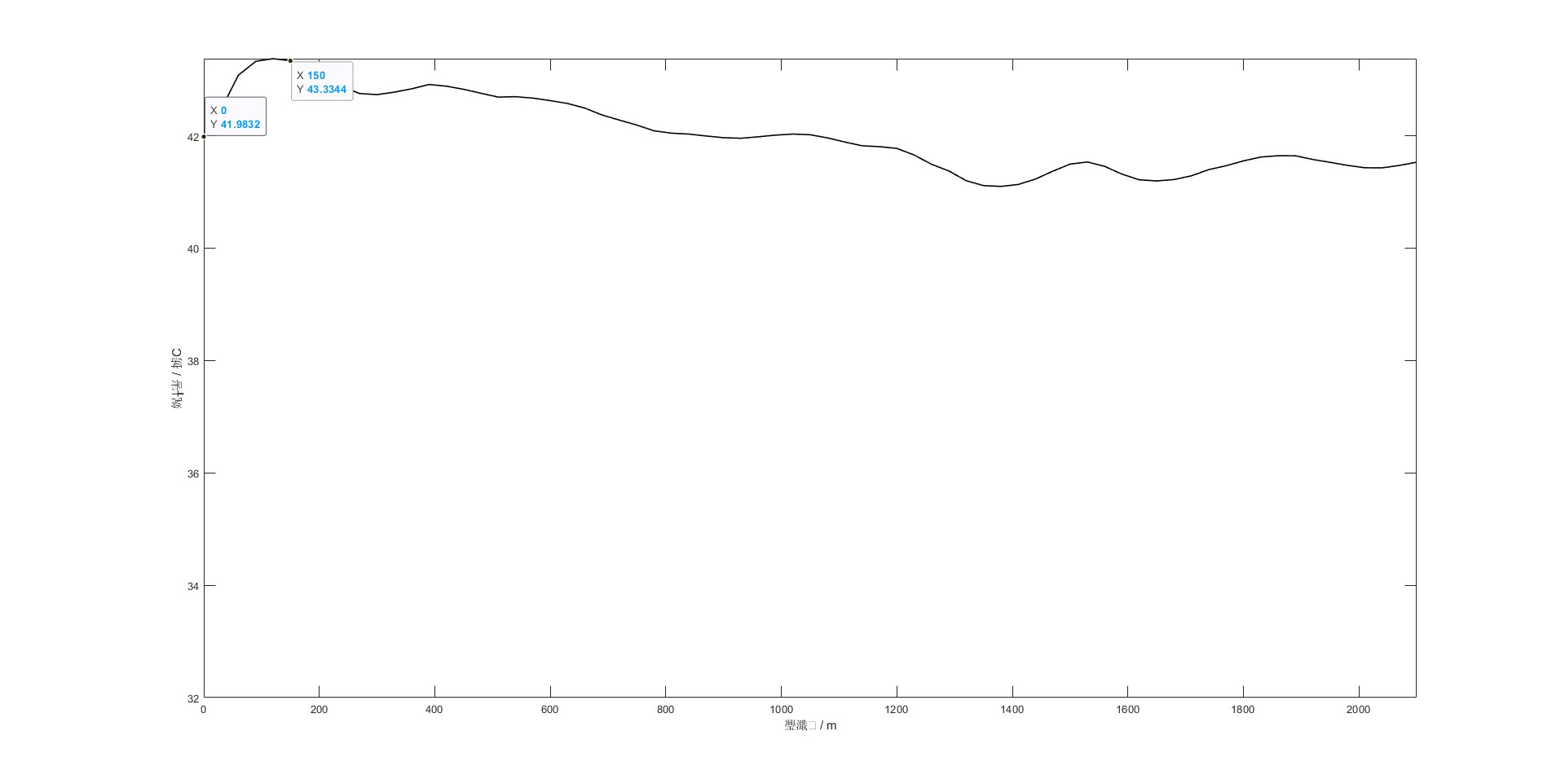

Supplement: S1 File — (ZIP) [file pone.0344026.s001.zip › Supplementary material/3 Matlab algorithms and some results/Park temperature inflection plot in Matlab/12.jpg]

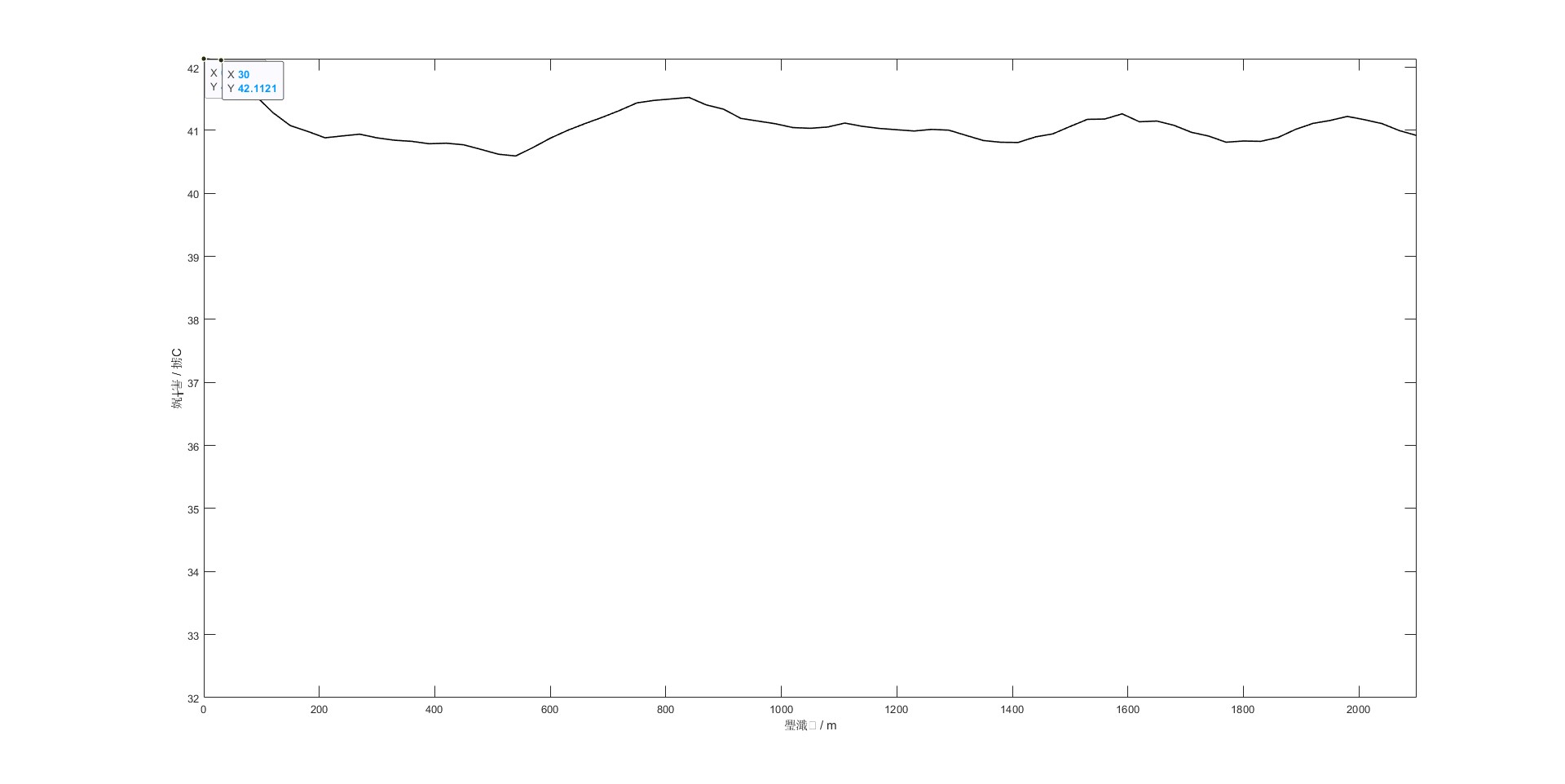

Supplement: S1 File — (ZIP) [file pone.0344026.s001.zip › Supplementary material/3 Matlab algorithms and some results/Park temperature inflection plot in Matlab/13.jpg]

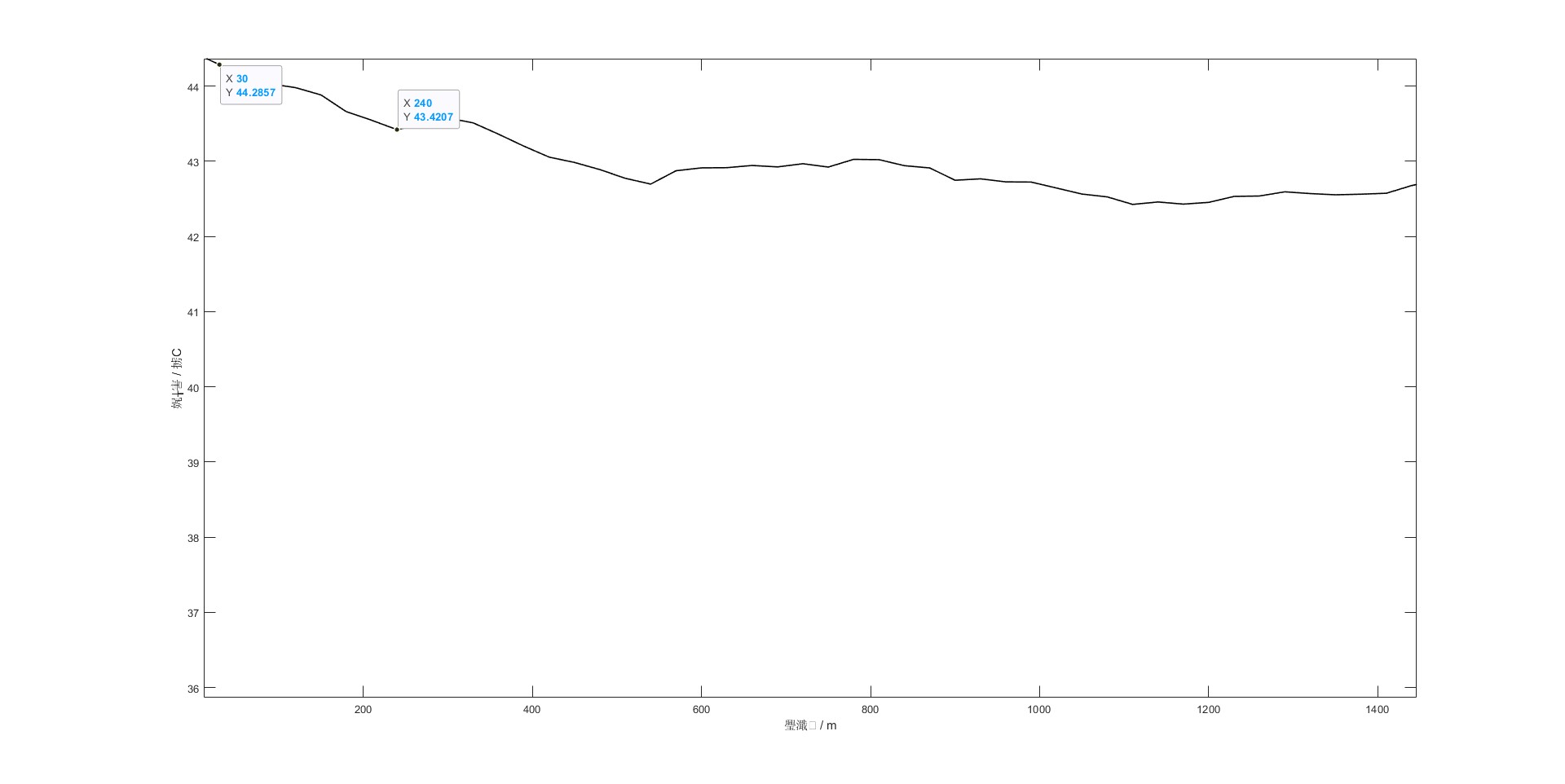

Supplement: S1 File — (ZIP) [file pone.0344026.s001.zip › Supplementary material/3 Matlab algorithms and some results/Park temperature inflection plot in Matlab/14.jpg]

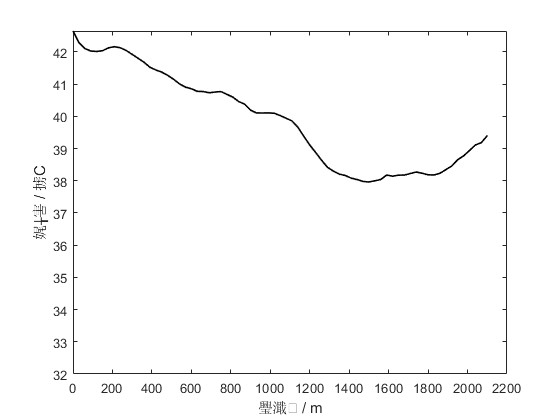

Supplement: S1 File — (ZIP) [file pone.0344026.s001.zip › Supplementary material/3 Matlab algorithms and some results/Park temperature inflection plot in Matlab/15.jpg]

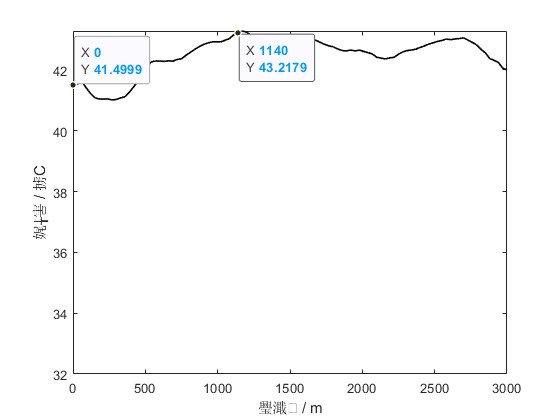

Supplement: S1 File — (ZIP) [file pone.0344026.s001.zip › Supplementary material/3 Matlab algorithms and some results/Park temperature inflection plot in Matlab/16.jpg]

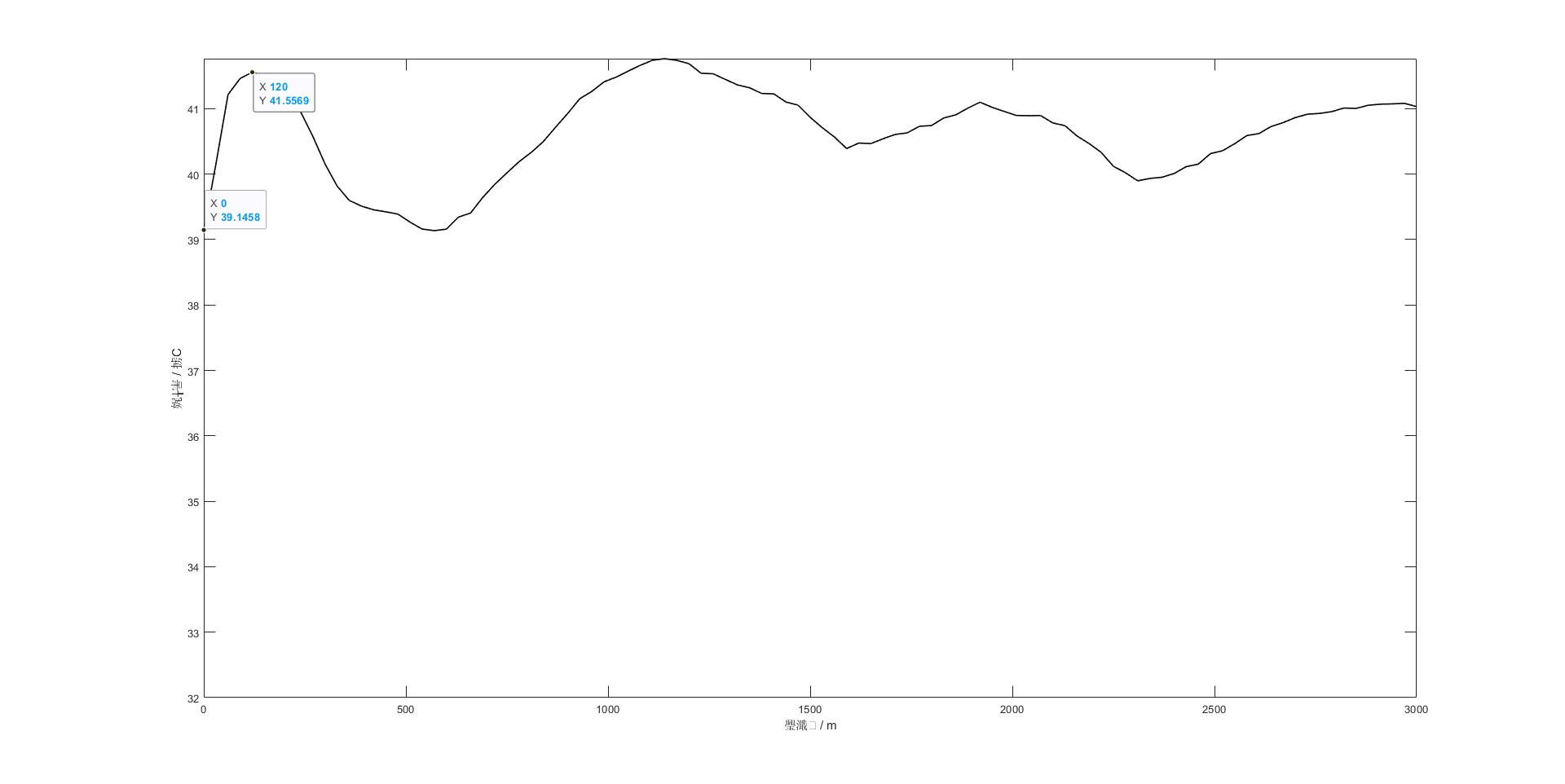

Supplement: S1 File — (ZIP) [file pone.0344026.s001.zip › Supplementary material/3 Matlab algorithms and some results/Park temperature inflection plot in Matlab/17.jpg]

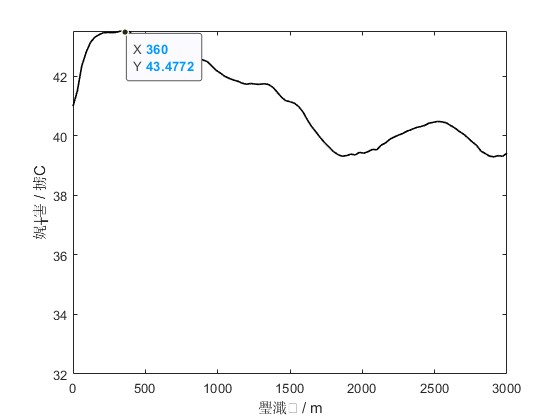

Supplement: S1 File — (ZIP) [file pone.0344026.s001.zip › Supplementary material/3 Matlab algorithms and some results/Park temperature inflection plot in Matlab/18.jpg]

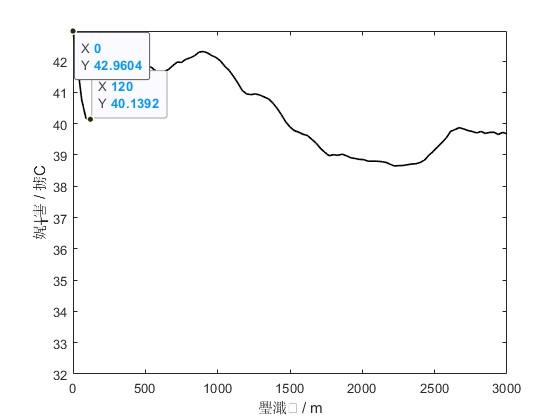

Supplement: S1 File — (ZIP) [file pone.0344026.s001.zip › Supplementary material/3 Matlab algorithms and some results/Park temperature inflection plot in Matlab/19.jpg]

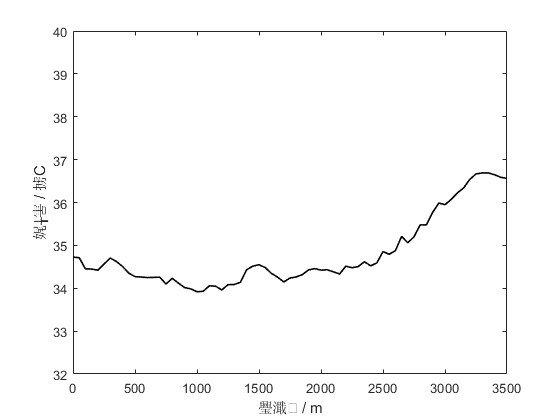

Supplement: S1 File — (ZIP) [file pone.0344026.s001.zip › Supplementary material/3 Matlab algorithms and some results/Park temperature inflection plot in Matlab/2.jpg]

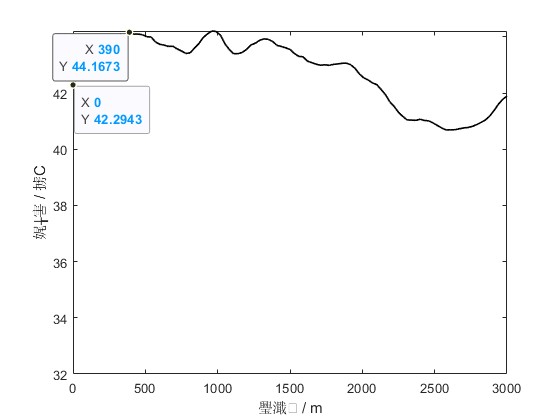

Supplement: S1 File — (ZIP) [file pone.0344026.s001.zip › Supplementary material/3 Matlab algorithms and some results/Park temperature inflection plot in Matlab/20.jpg]

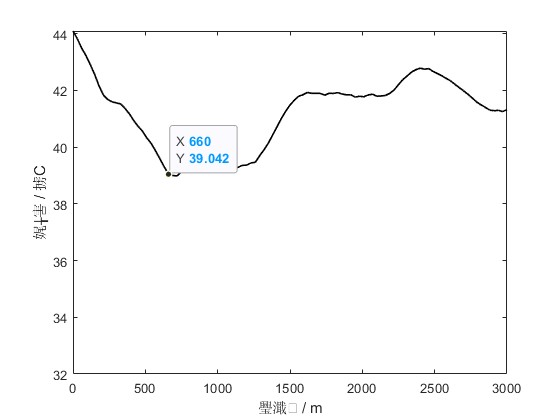

Supplement: S1 File — (ZIP) [file pone.0344026.s001.zip › Supplementary material/3 Matlab algorithms and some results/Park temperature inflection plot in Matlab/21.jpg]

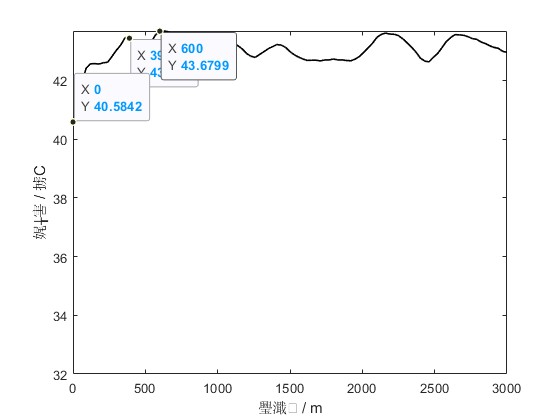

Supplement: S1 File — (ZIP) [file pone.0344026.s001.zip › Supplementary material/3 Matlab algorithms and some results/Park temperature inflection plot in Matlab/22.jpg]

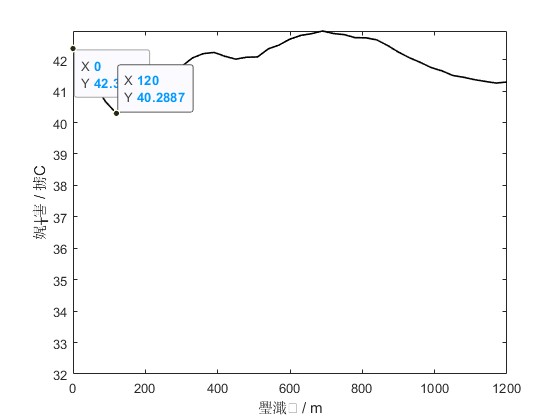

Supplement: S1 File — (ZIP) [file pone.0344026.s001.zip › Supplementary material/3 Matlab algorithms and some results/Park temperature inflection plot in Matlab/23.jpg]

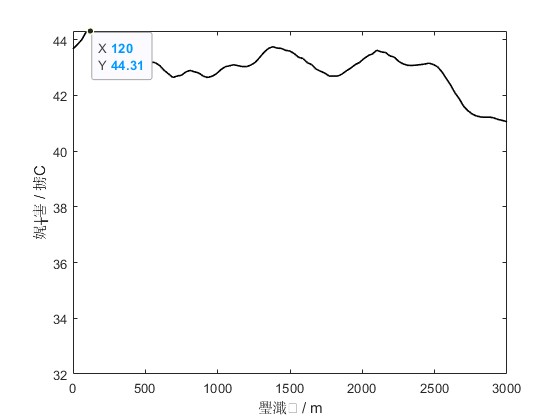

Supplement: S1 File — (ZIP) [file pone.0344026.s001.zip › Supplementary material/3 Matlab algorithms and some results/Park temperature inflection plot in Matlab/24SJT.jpg]

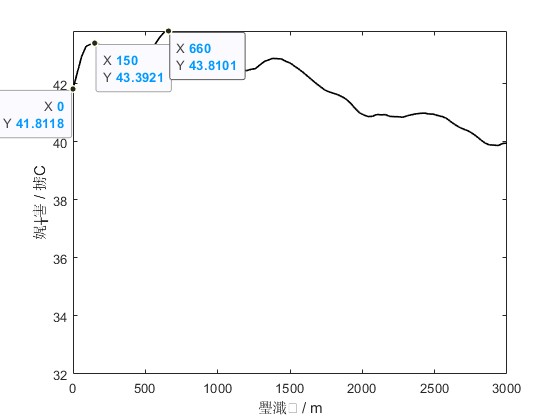

Supplement: S1 File — (ZIP) [file pone.0344026.s001.zip › Supplementary material/3 Matlab algorithms and some results/Park temperature inflection plot in Matlab/25.jpg]

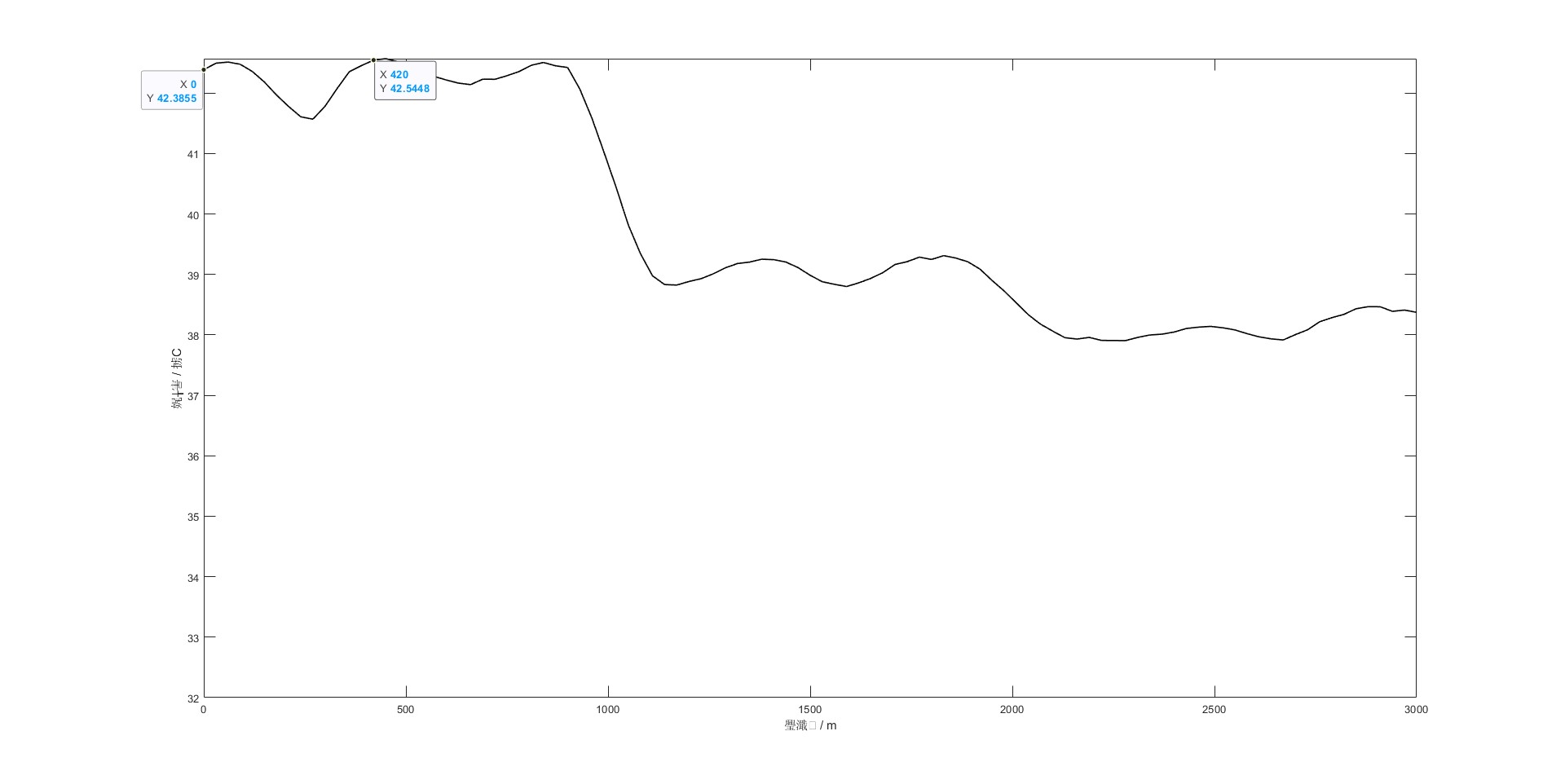

Supplement: S1 File — (ZIP) [file pone.0344026.s001.zip › Supplementary material/3 Matlab algorithms and some results/Park temperature inflection plot in Matlab/26.jpg]

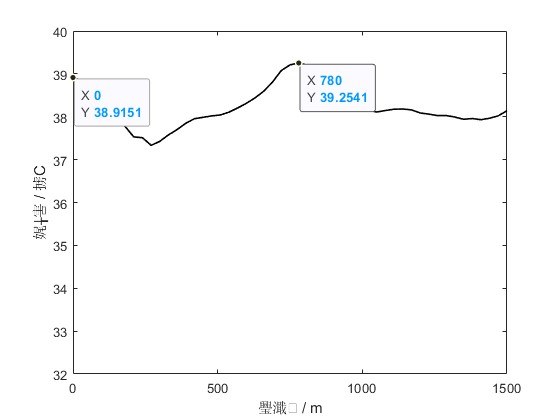

Supplement: S1 File — (ZIP) [file pone.0344026.s001.zip › Supplementary material/3 Matlab algorithms and some results/Park temperature inflection plot in Matlab/27.jpg]

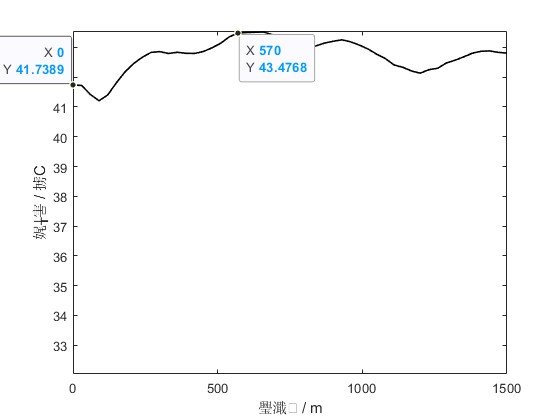

Supplement: S1 File — (ZIP) [file pone.0344026.s001.zip › Supplementary material/3 Matlab algorithms and some results/Park temperature inflection plot in Matlab/28.jpg]

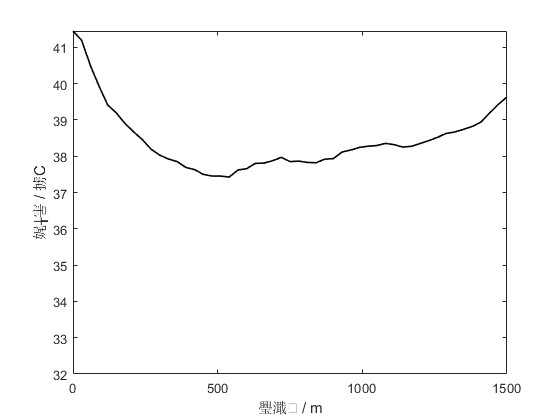

Supplement: S1 File — (ZIP) [file pone.0344026.s001.zip › Supplementary material/3 Matlab algorithms and some results/Park temperature inflection plot in Matlab/29.jpg]

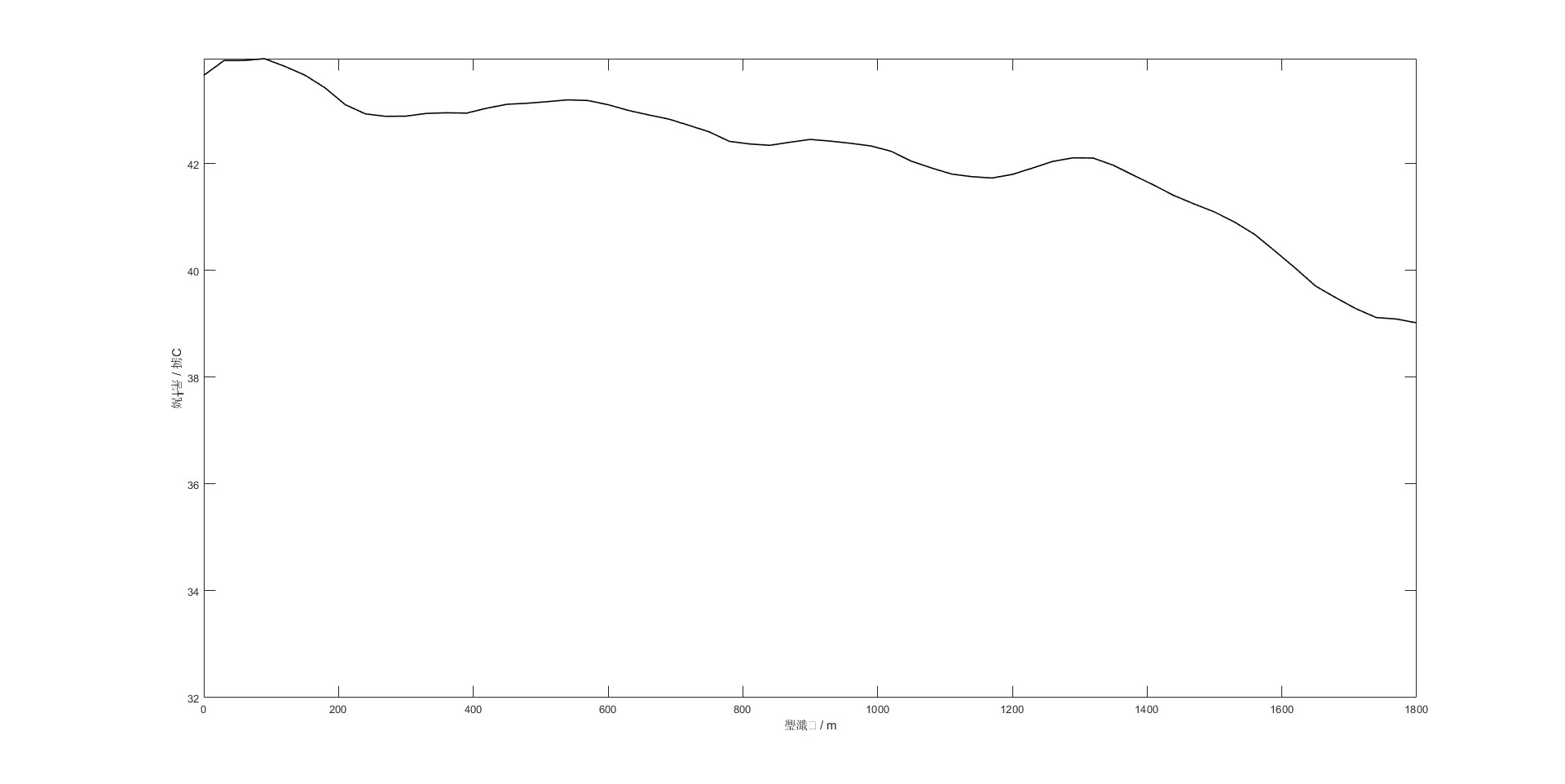

Supplement: S1 File — (ZIP) [file pone.0344026.s001.zip › Supplementary material/3 Matlab algorithms and some results/Park temperature inflection plot in Matlab/3.jpg]

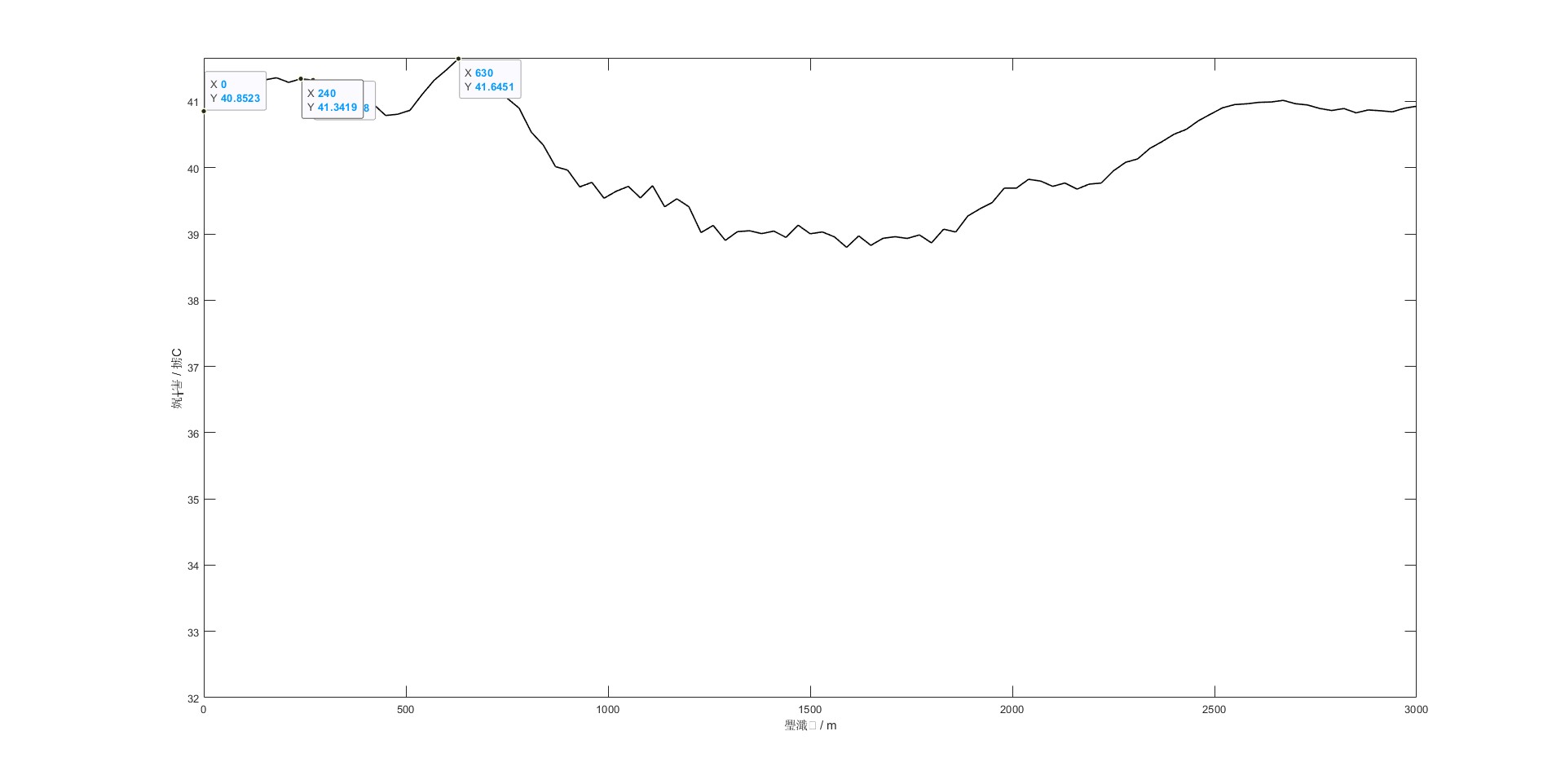

Supplement: S1 File — (ZIP) [file pone.0344026.s001.zip › Supplementary material/3 Matlab algorithms and some results/Park temperature inflection plot in Matlab/30.jpg]

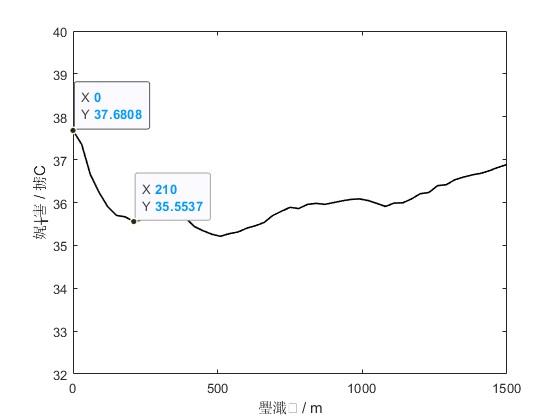

Supplement: S1 File — (ZIP) [file pone.0344026.s001.zip › Supplementary material/3 Matlab algorithms and some results/Park temperature inflection plot in Matlab/31.jpg]

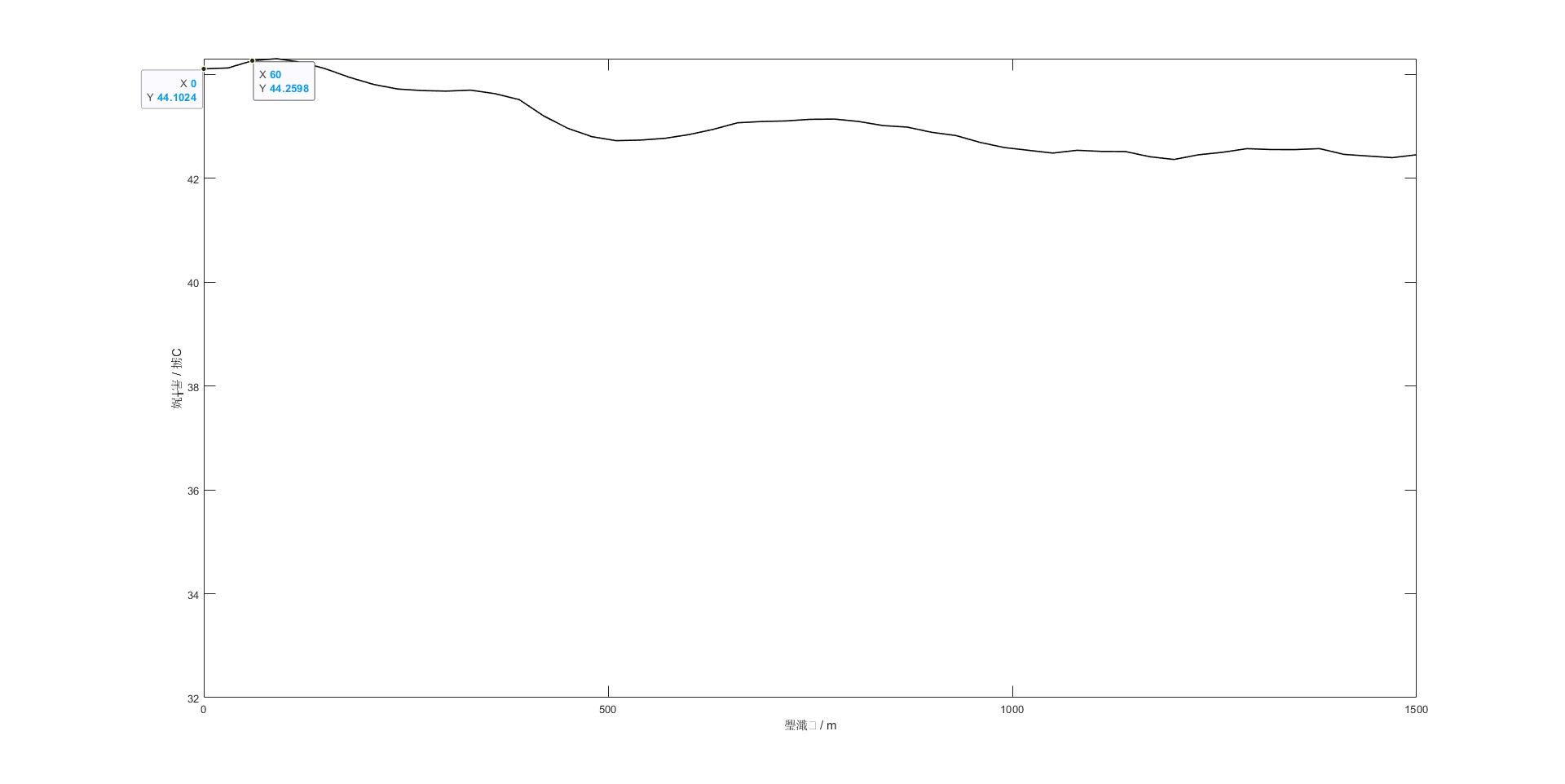

Supplement: S1 File — (ZIP) [file pone.0344026.s001.zip › Supplementary material/3 Matlab algorithms and some results/Park temperature inflection plot in Matlab/32.jpg]

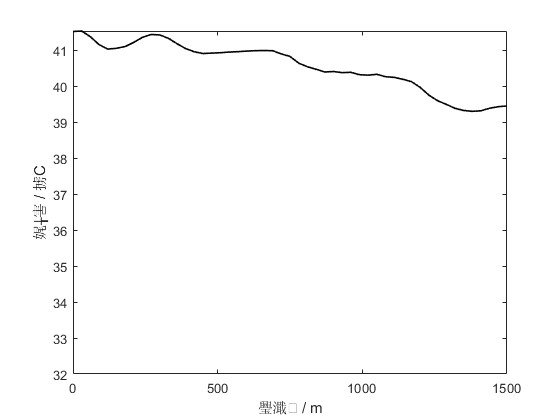

Supplement: S1 File — (ZIP) [file pone.0344026.s001.zip › Supplementary material/3 Matlab algorithms and some results/Park temperature inflection plot in Matlab/33.jpg]

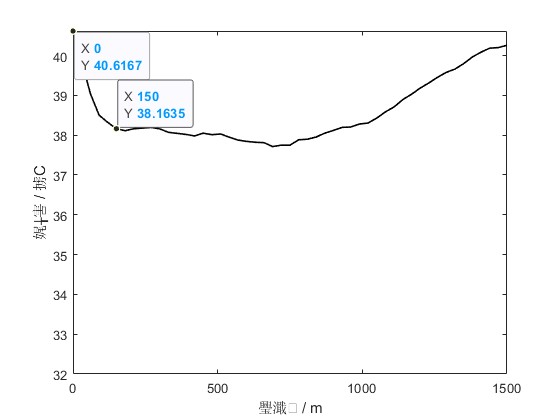

Supplement: S1 File — (ZIP) [file pone.0344026.s001.zip › Supplementary material/3 Matlab algorithms and some results/Park temperature inflection plot in Matlab/34.jpg]

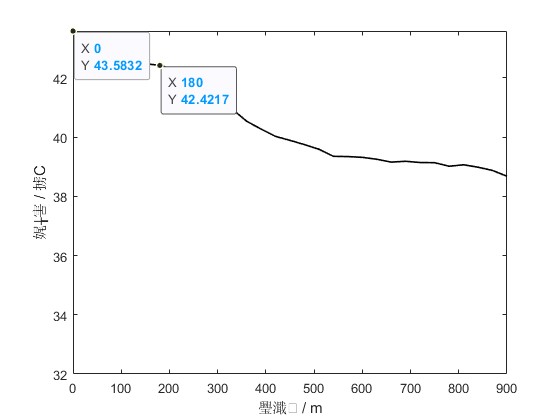

Supplement: S1 File — (ZIP) [file pone.0344026.s001.zip › Supplementary material/3 Matlab algorithms and some results/Park temperature inflection plot in Matlab/35.jpg]

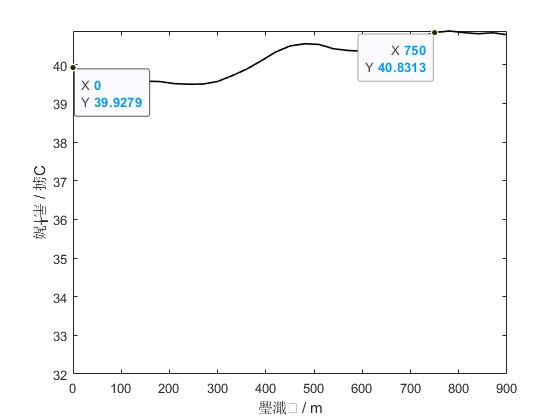

Supplement: S1 File — (ZIP) [file pone.0344026.s001.zip › Supplementary material/3 Matlab algorithms and some results/Park temperature inflection plot in Matlab/36.jpg]

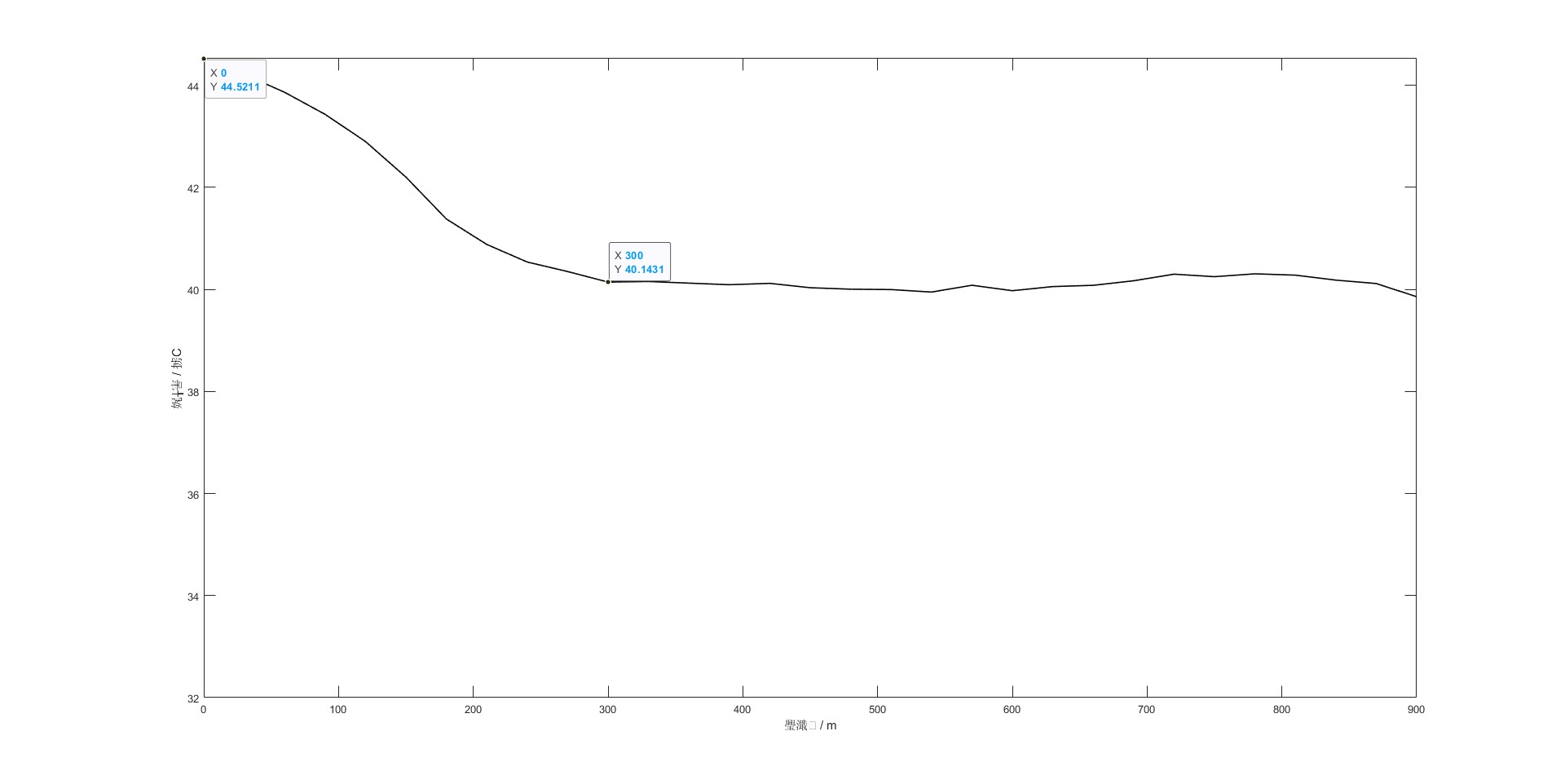

Supplement: S1 File — (ZIP) [file pone.0344026.s001.zip › Supplementary material/3 Matlab algorithms and some results/Park temperature inflection plot in Matlab/37.jpg]

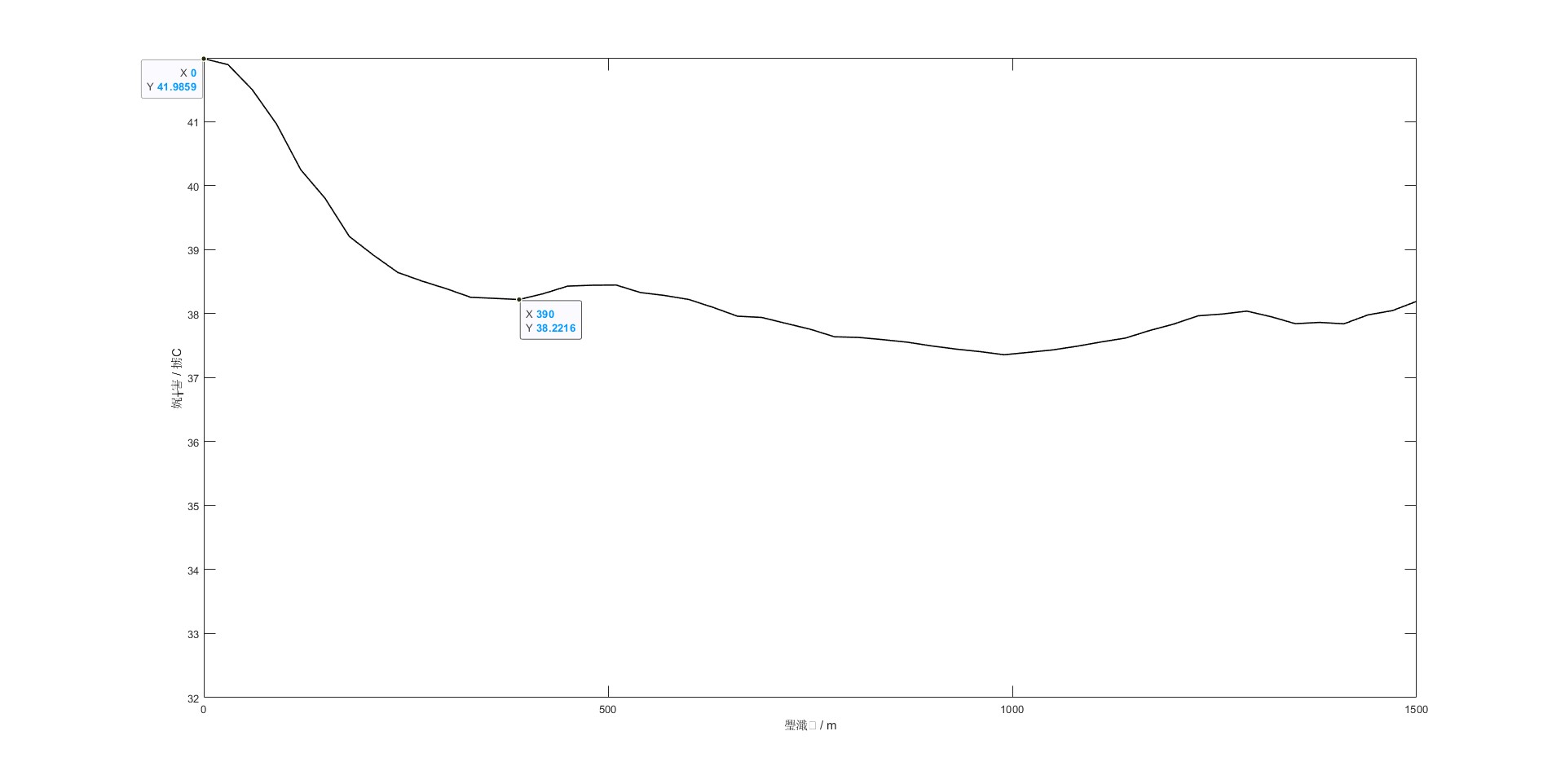

Supplement: S1 File — (ZIP) [file pone.0344026.s001.zip › Supplementary material/3 Matlab algorithms and some results/Park temperature inflection plot in Matlab/38.jpg]

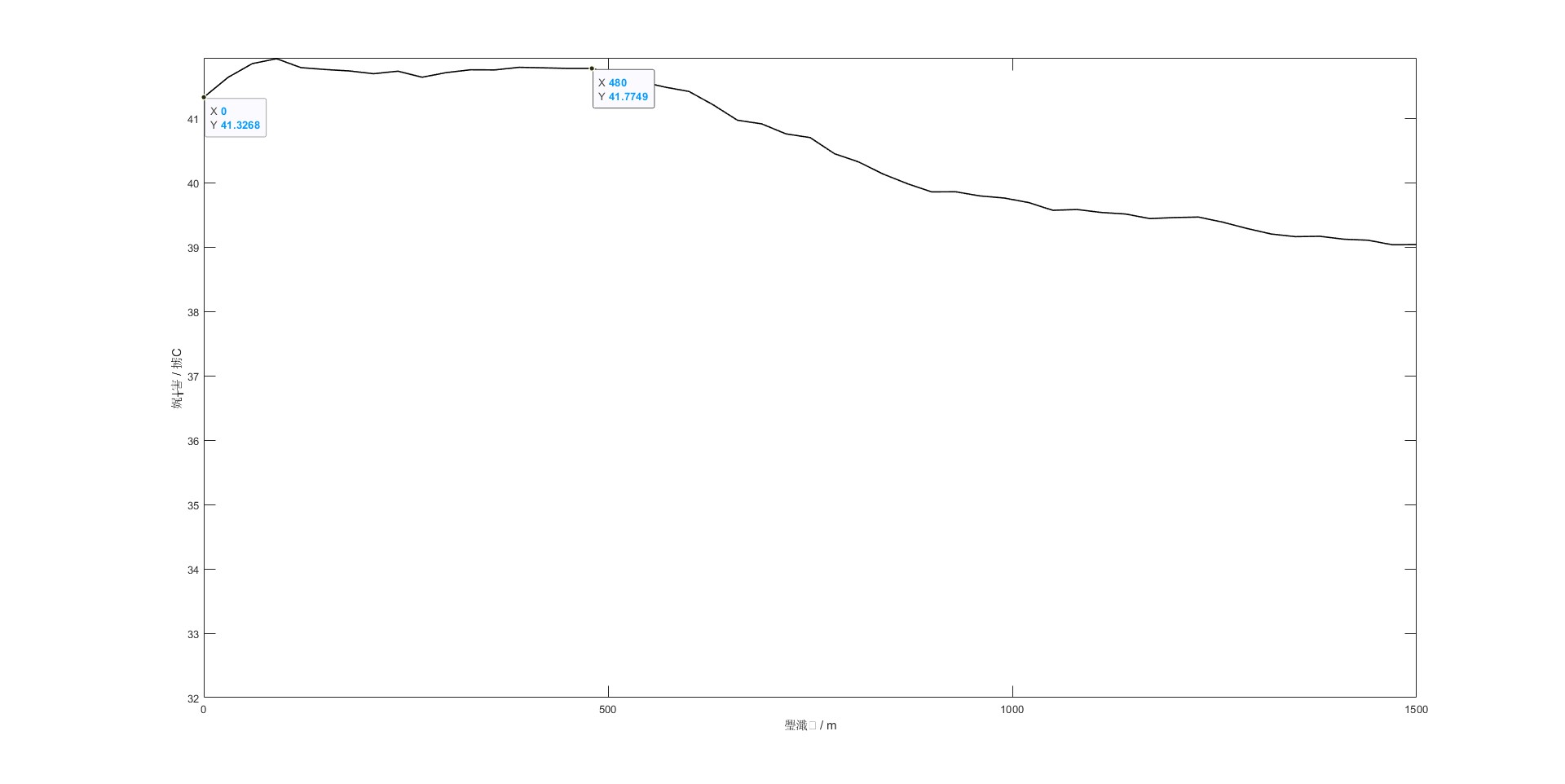

Supplement: S1 File — (ZIP) [file pone.0344026.s001.zip › Supplementary material/3 Matlab algorithms and some results/Park temperature inflection plot in Matlab/39.jpg]

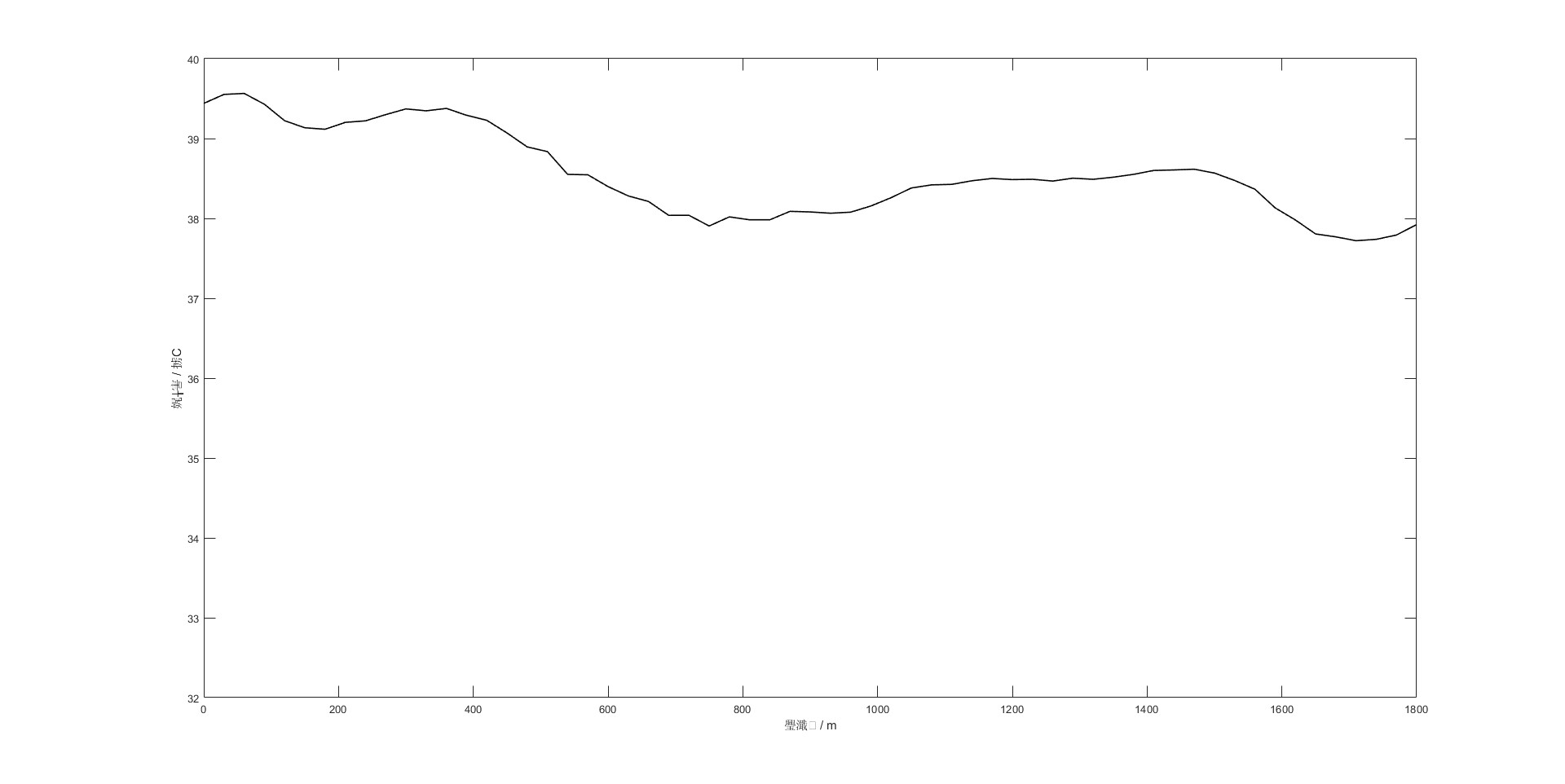

Supplement: S1 File — (ZIP) [file pone.0344026.s001.zip › Supplementary material/3 Matlab algorithms and some results/Park temperature inflection plot in Matlab/4.jpg]

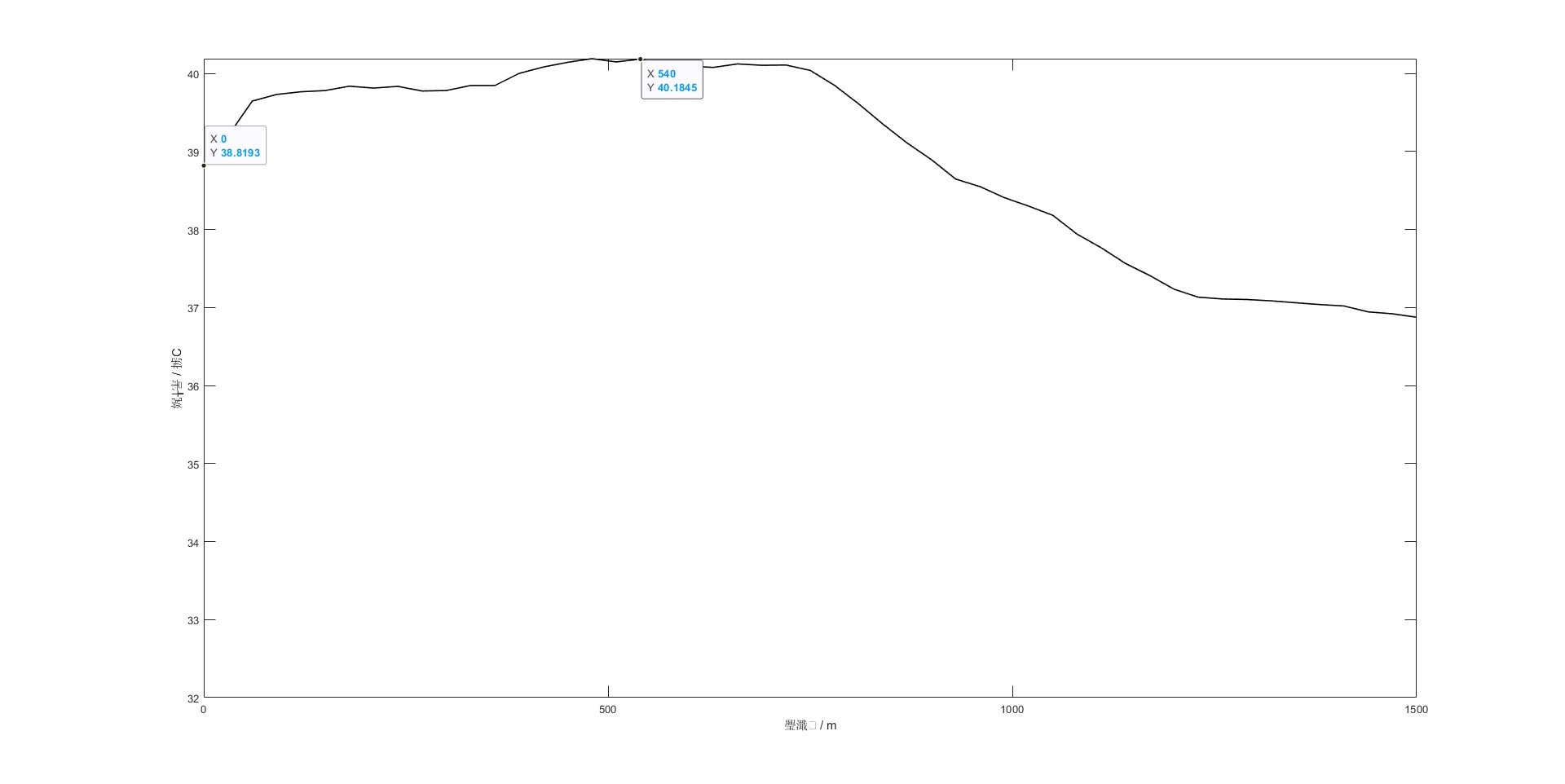

Supplement: S1 File — (ZIP) [file pone.0344026.s001.zip › Supplementary material/3 Matlab algorithms and some results/Park temperature inflection plot in Matlab/40.jpg]

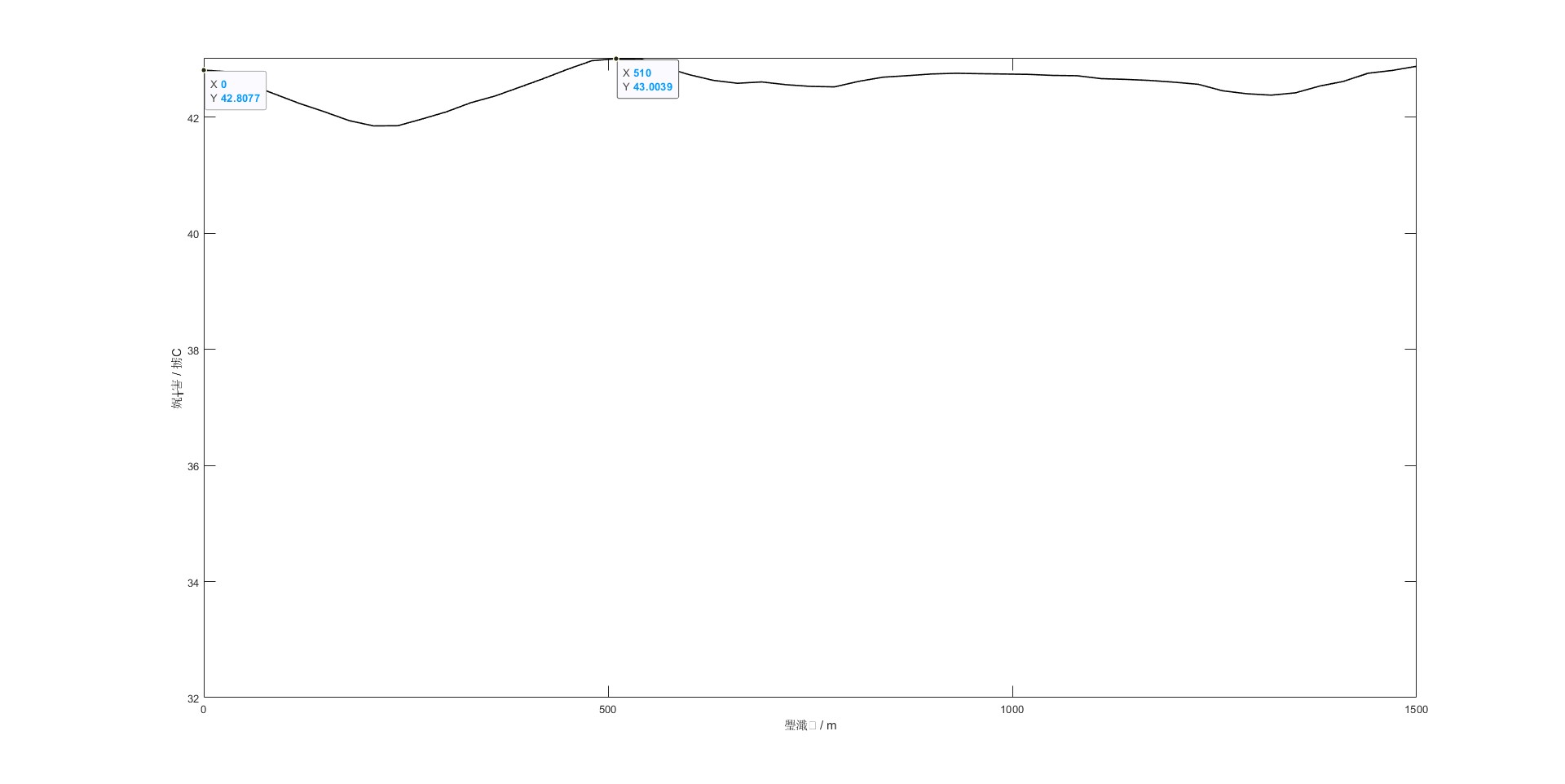

Supplement: S1 File — (ZIP) [file pone.0344026.s001.zip › Supplementary material/3 Matlab algorithms and some results/Park temperature inflection plot in Matlab/41.jpg]

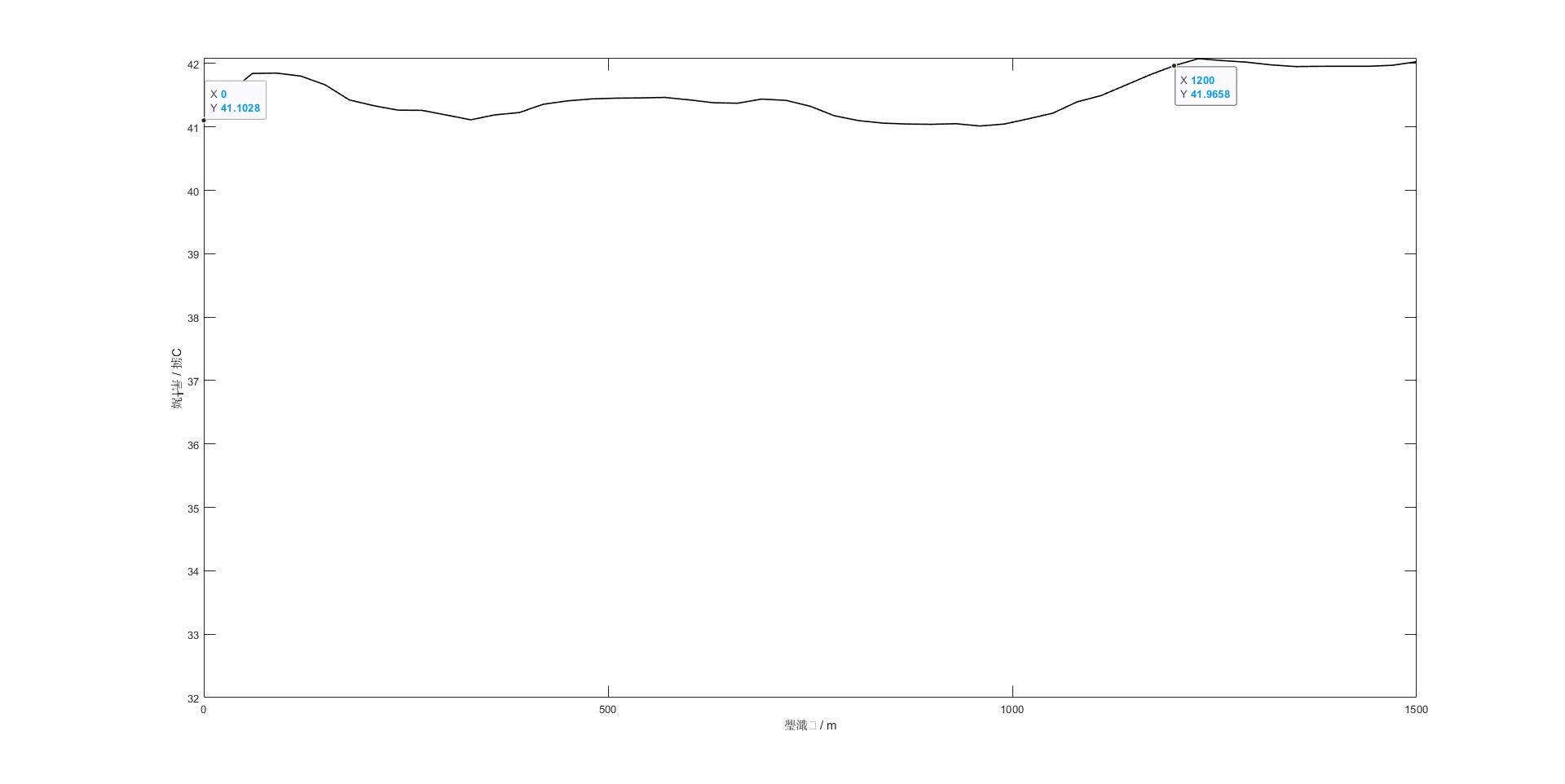

Supplement: S1 File — (ZIP) [file pone.0344026.s001.zip › Supplementary material/3 Matlab algorithms and some results/Park temperature inflection plot in Matlab/42MLW.jpg]

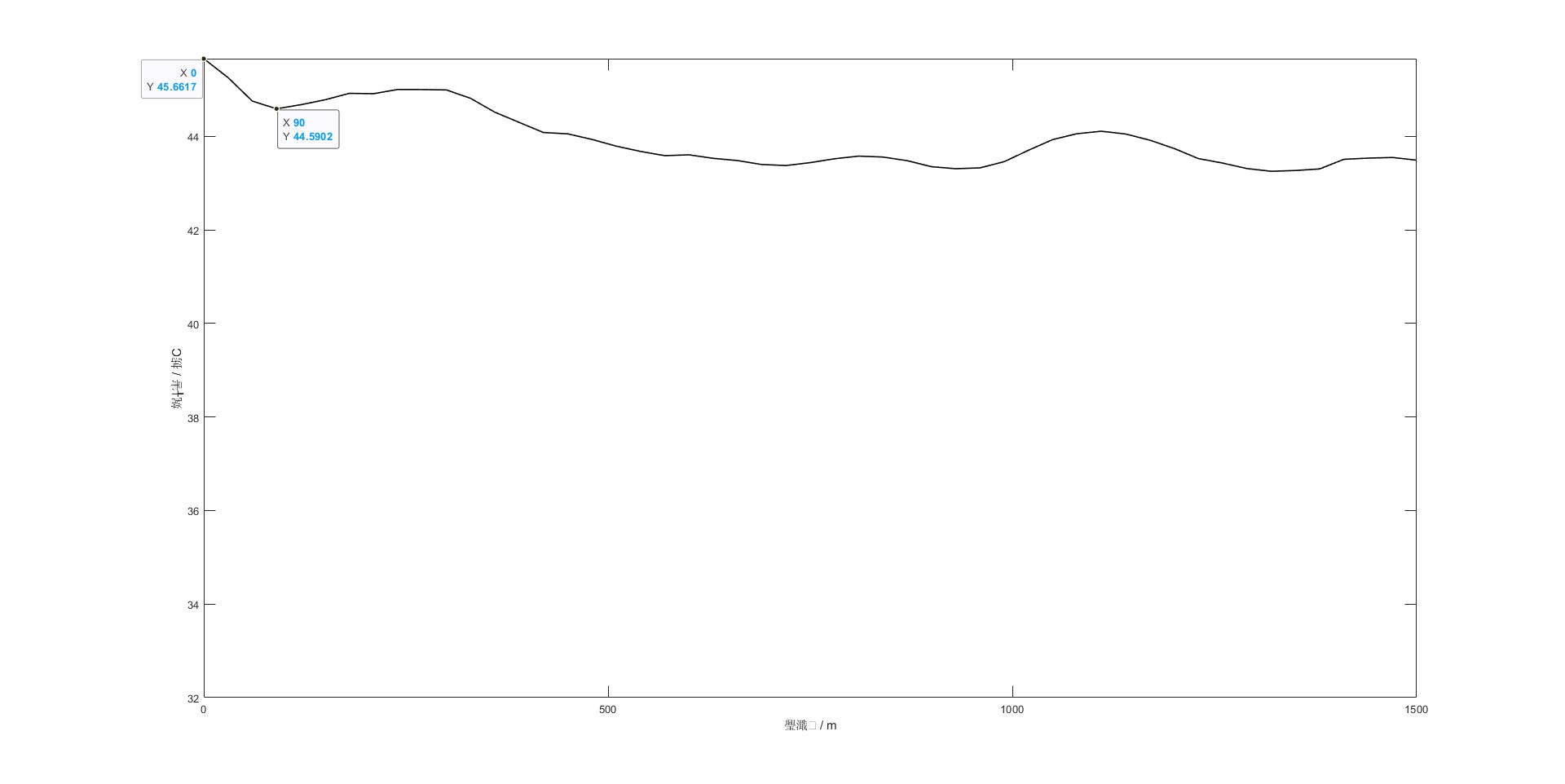

Supplement: S1 File — (ZIP) [file pone.0344026.s001.zip › Supplementary material/3 Matlab algorithms and some results/Park temperature inflection plot in Matlab/43.jpg]

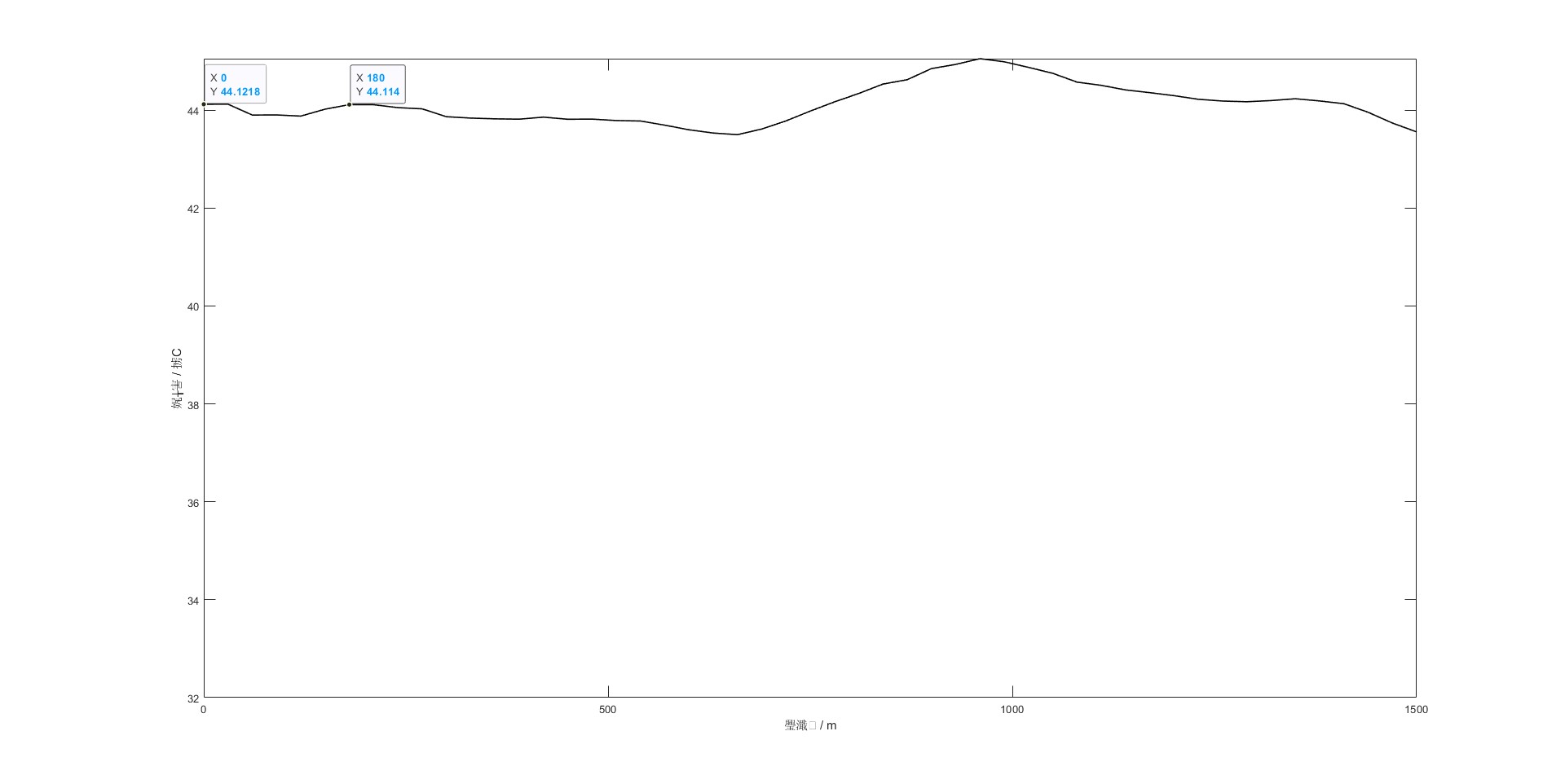

Supplement: S1 File — (ZIP) [file pone.0344026.s001.zip › Supplementary material/3 Matlab algorithms and some results/Park temperature inflection plot in Matlab/44.jpg]

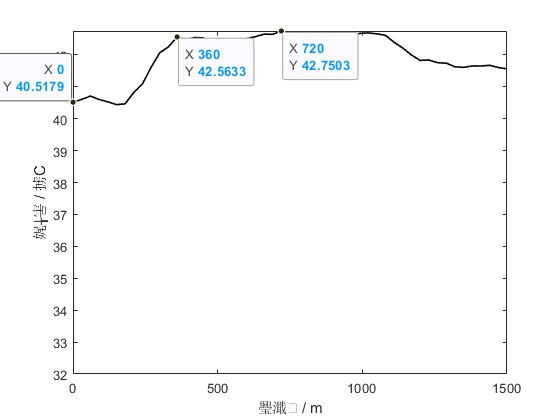

Supplement: S1 File — (ZIP) [file pone.0344026.s001.zip › Supplementary material/3 Matlab algorithms and some results/Park temperature inflection plot in Matlab/45.jpg]

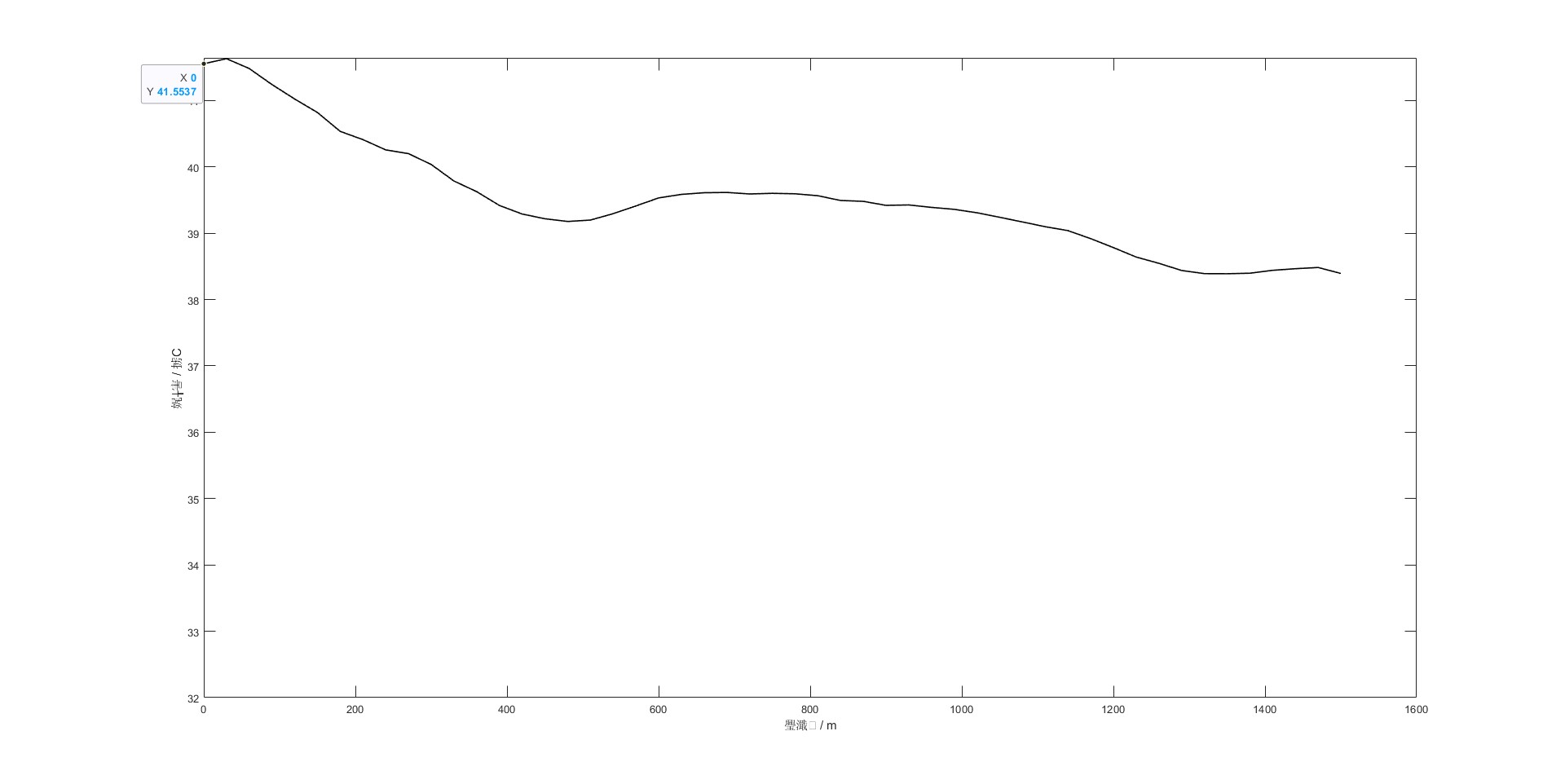

Supplement: S1 File — (ZIP) [file pone.0344026.s001.zip › Supplementary material/3 Matlab algorithms and some results/Park temperature inflection plot in Matlab/46.jpg]

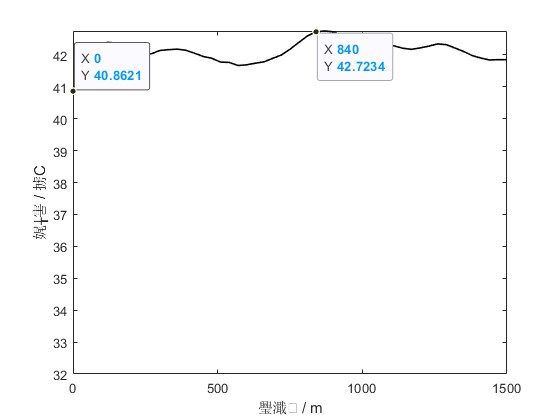

Supplement: S1 File — (ZIP) [file pone.0344026.s001.zip › Supplementary material/3 Matlab algorithms and some results/Park temperature inflection plot in Matlab/47.jpg]

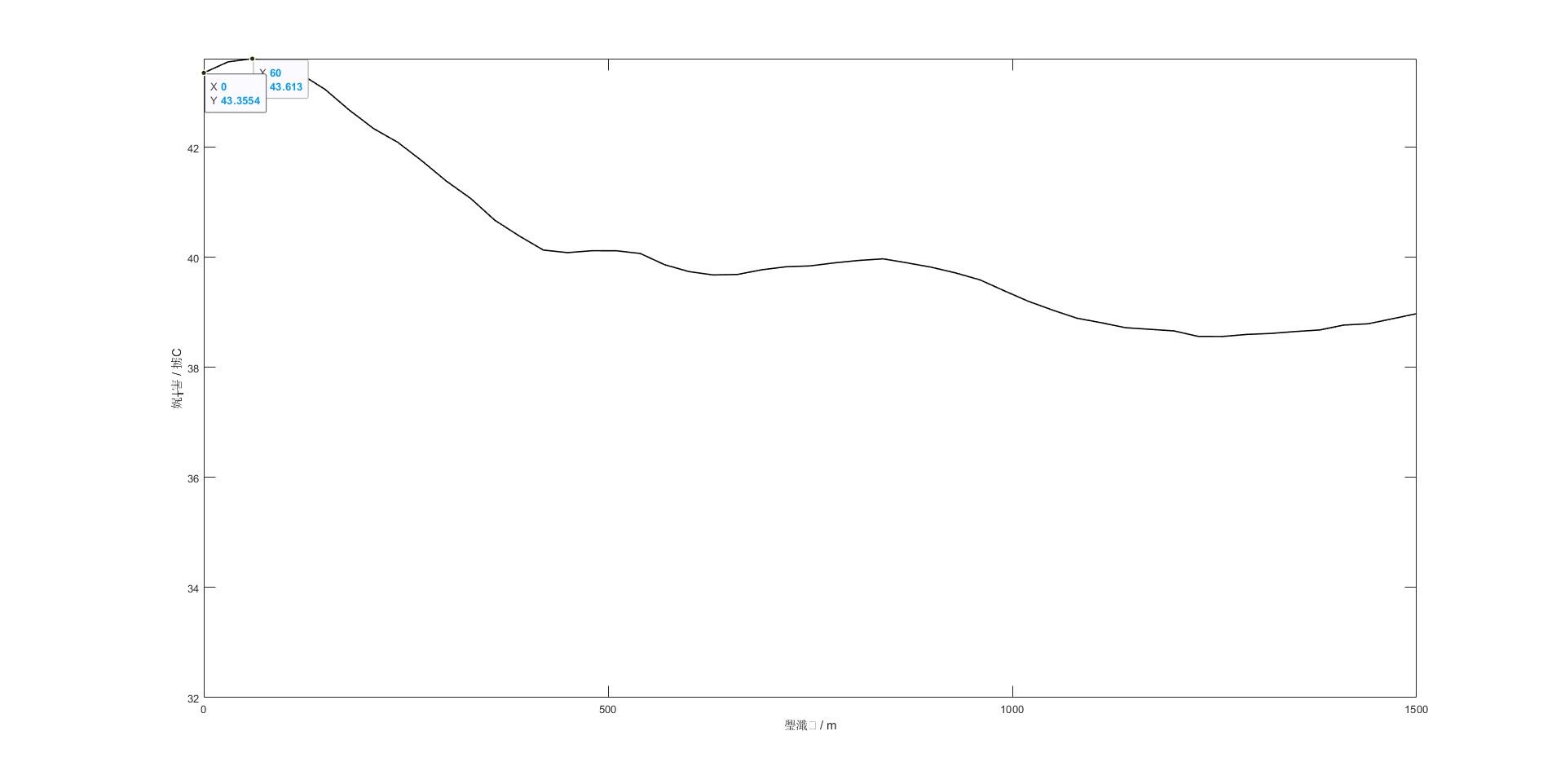

Supplement: S1 File — (ZIP) [file pone.0344026.s001.zip › Supplementary material/3 Matlab algorithms and some results/Park temperature inflection plot in Matlab/48.jpg]

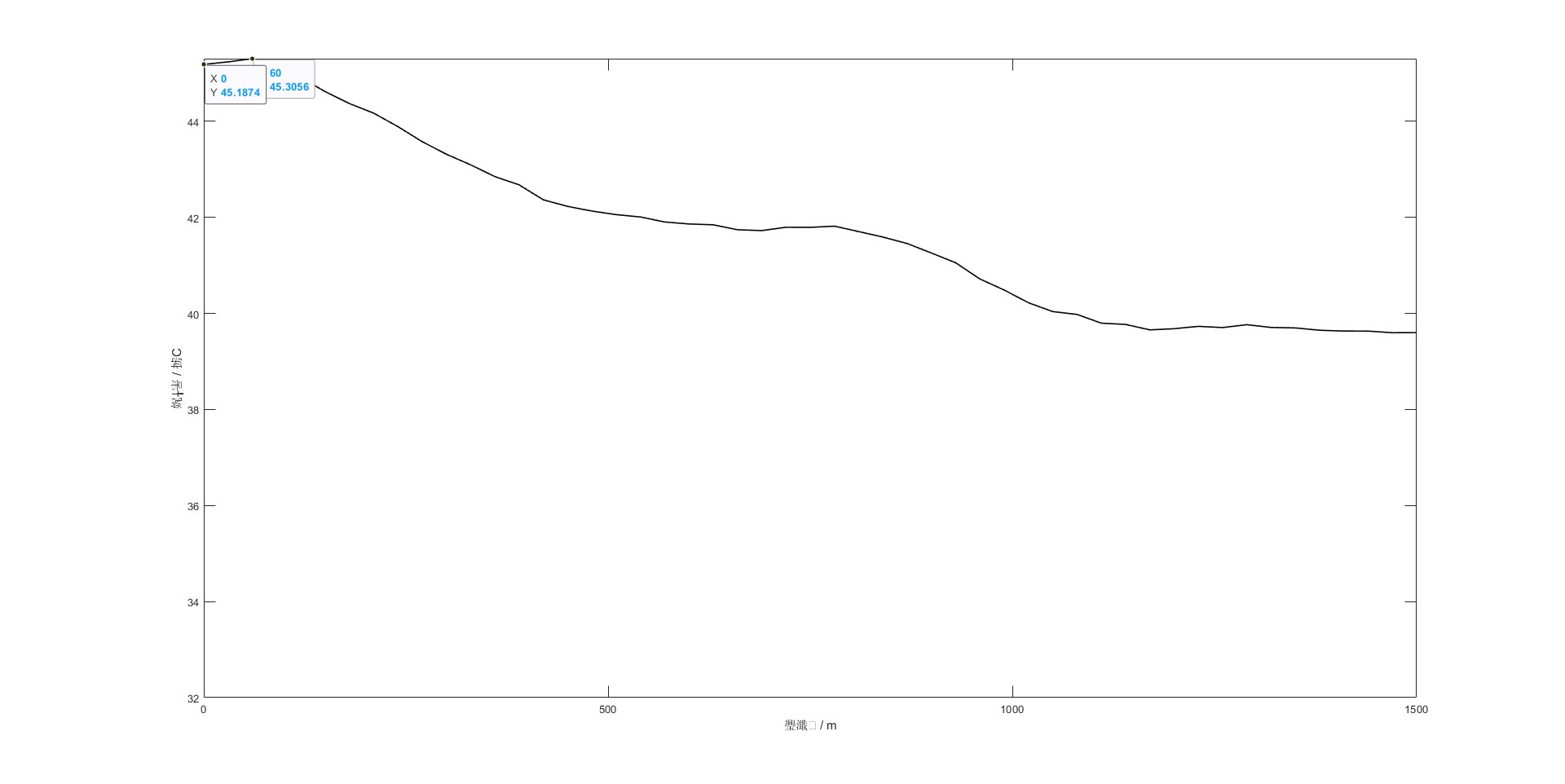

Supplement: S1 File — (ZIP) [file pone.0344026.s001.zip › Supplementary material/3 Matlab algorithms and some results/Park temperature inflection plot in Matlab/49.jpg]

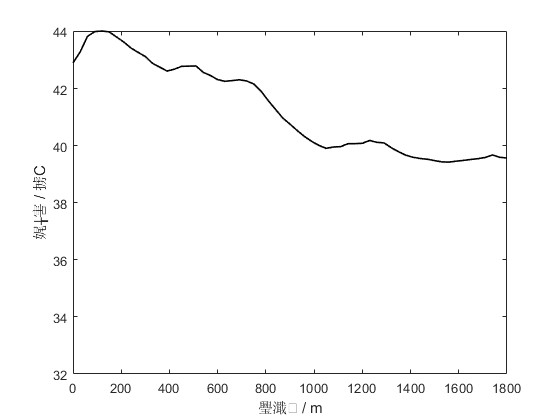

Supplement: S1 File — (ZIP) [file pone.0344026.s001.zip › Supplementary material/3 Matlab algorithms and some results/Park temperature inflection plot in Matlab/5.jpg]

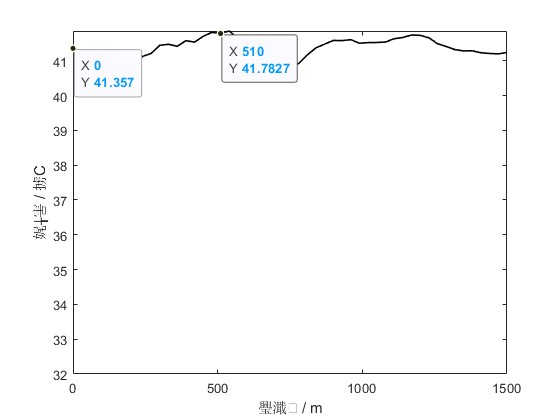

Supplement: S1 File — (ZIP) [file pone.0344026.s001.zip › Supplementary material/3 Matlab algorithms and some results/Park temperature inflection plot in Matlab/50.jpg]

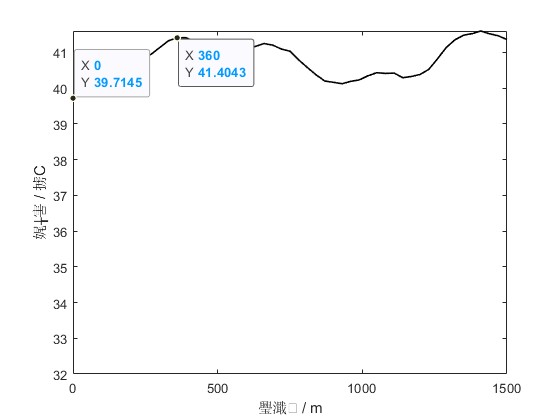

Supplement: S1 File — (ZIP) [file pone.0344026.s001.zip › Supplementary material/3 Matlab algorithms and some results/Park temperature inflection plot in Matlab/51.jpg]

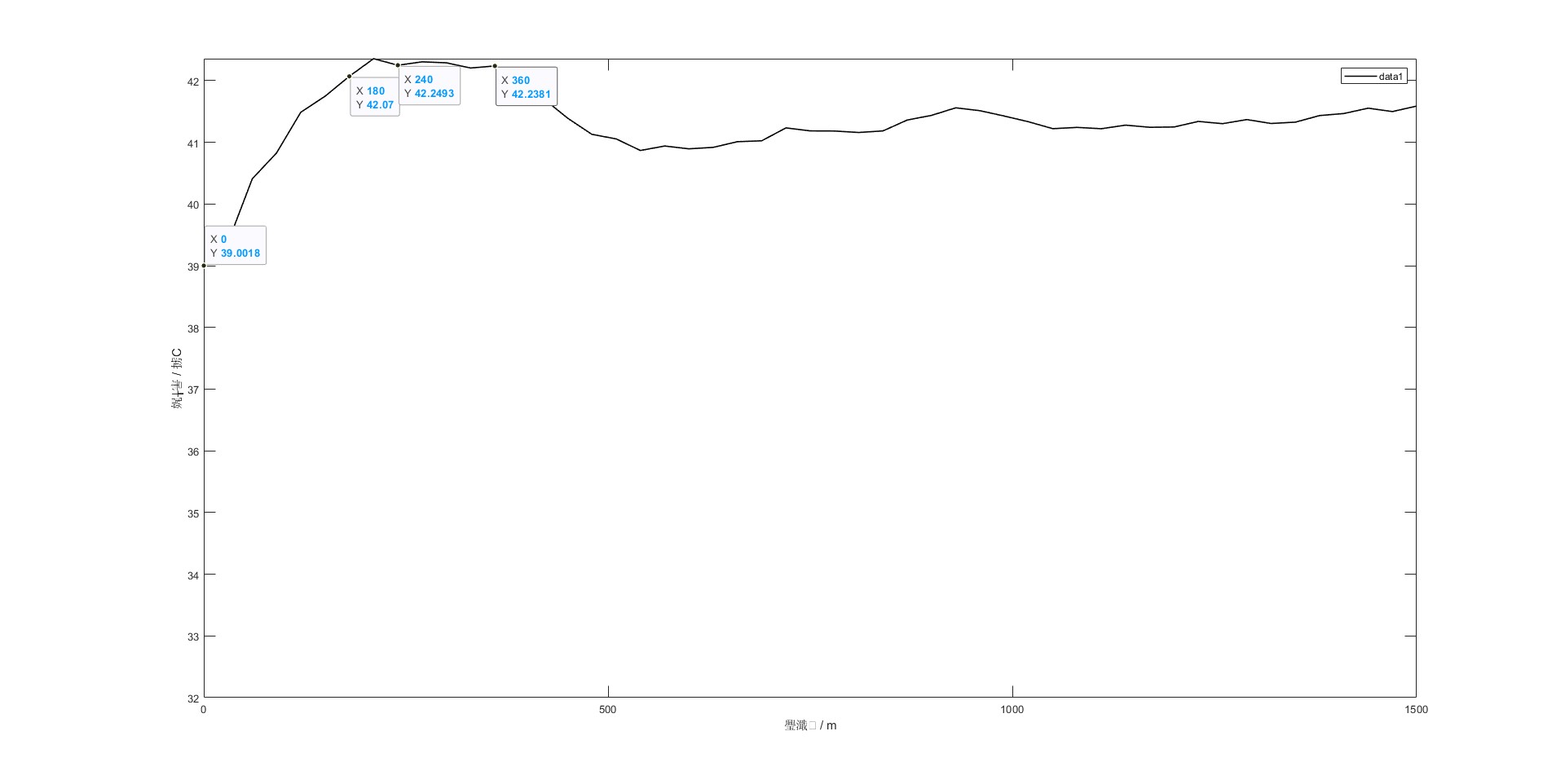

Supplement: S1 File — (ZIP) [file pone.0344026.s001.zip › Supplementary material/3 Matlab algorithms and some results/Park temperature inflection plot in Matlab/52.jpg]

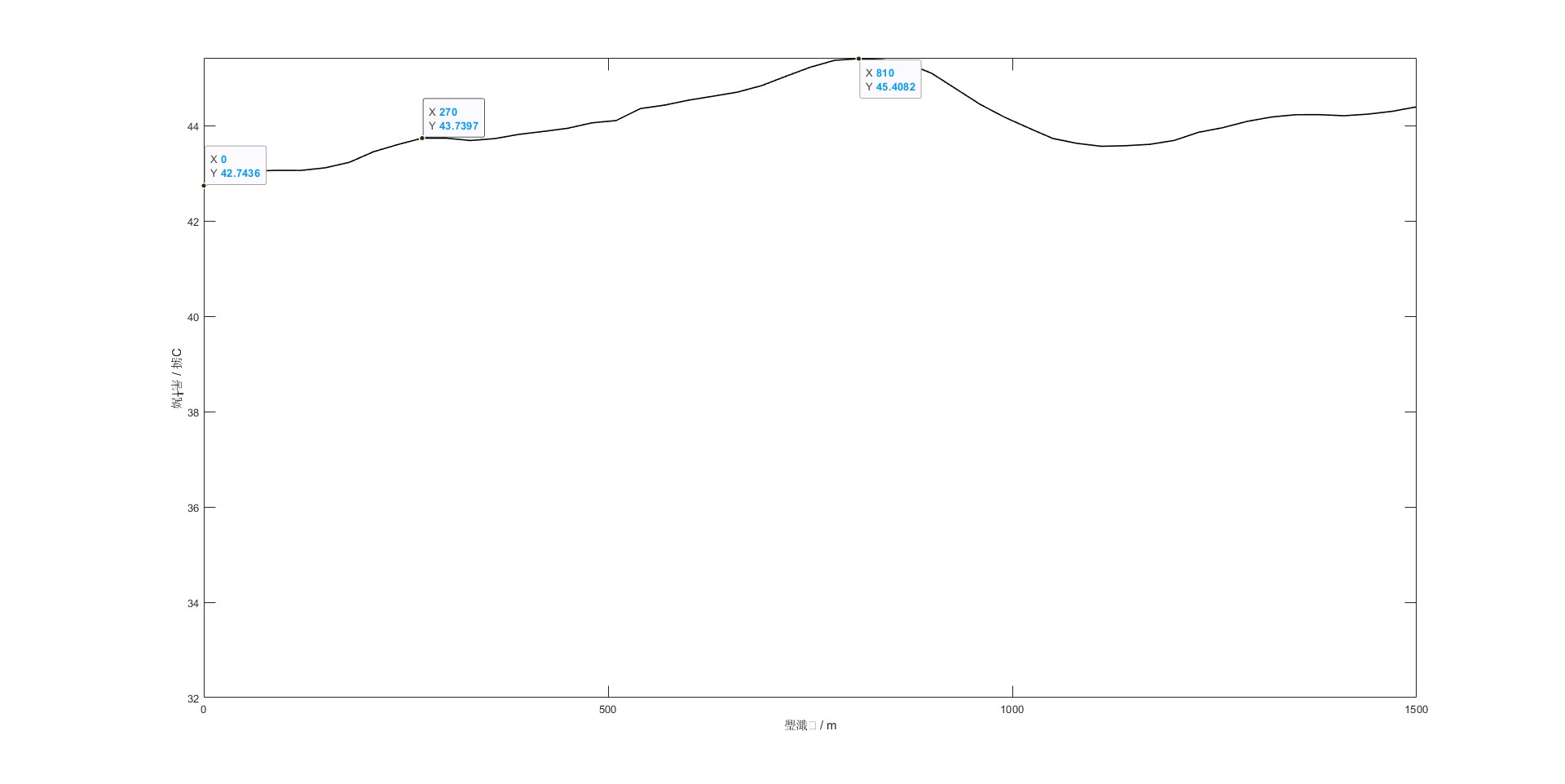

Supplement: S1 File — (ZIP) [file pone.0344026.s001.zip › Supplementary material/3 Matlab algorithms and some results/Park temperature inflection plot in Matlab/53.jpg]

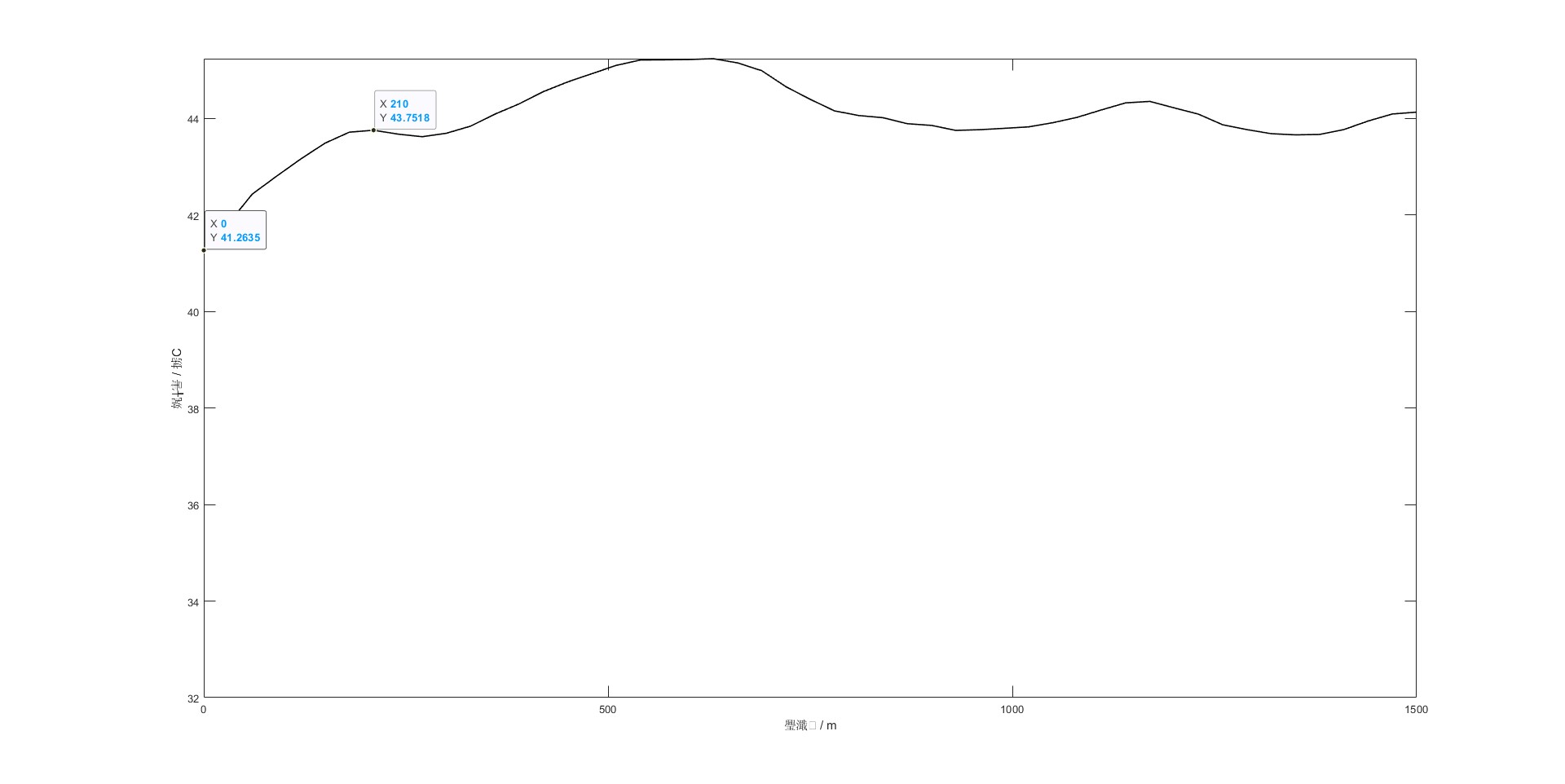

Supplement: S1 File — (ZIP) [file pone.0344026.s001.zip › Supplementary material/3 Matlab algorithms and some results/Park temperature inflection plot in Matlab/54.jpg]

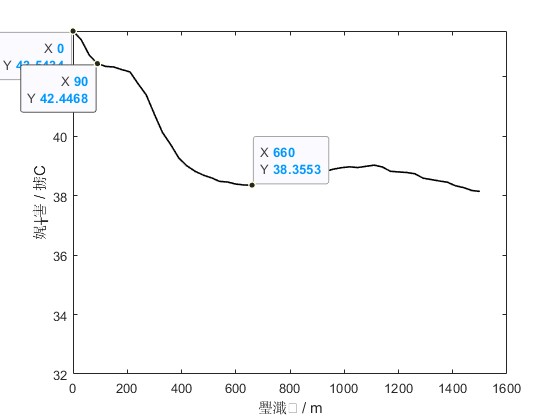

Supplement: S1 File — (ZIP) [file pone.0344026.s001.zip › Supplementary material/3 Matlab algorithms and some results/Park temperature inflection plot in Matlab/55.jpg]

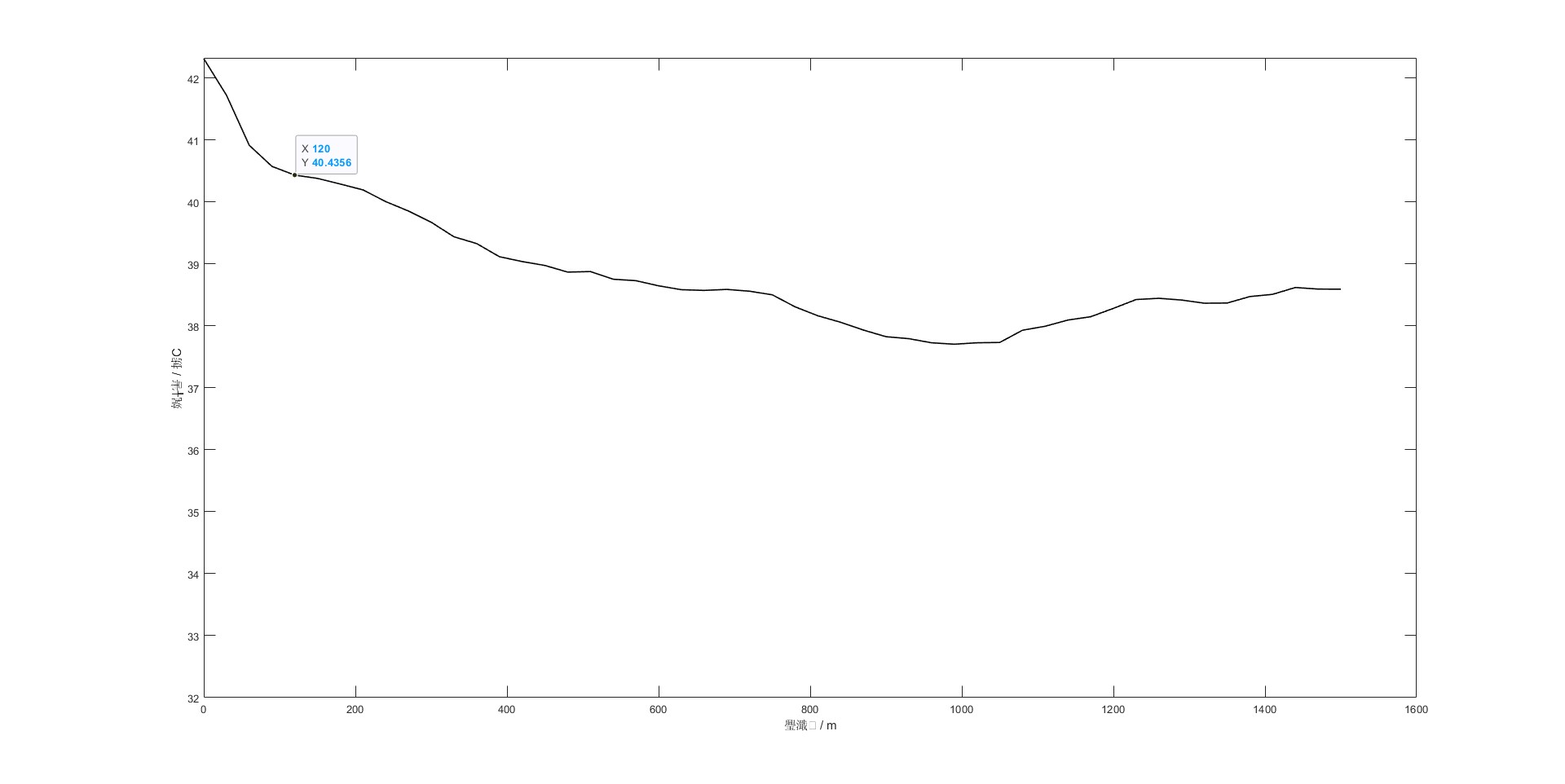

Supplement: S1 File — (ZIP) [file pone.0344026.s001.zip › Supplementary material/3 Matlab algorithms and some results/Park temperature inflection plot in Matlab/56 GXTYGY.jpg]

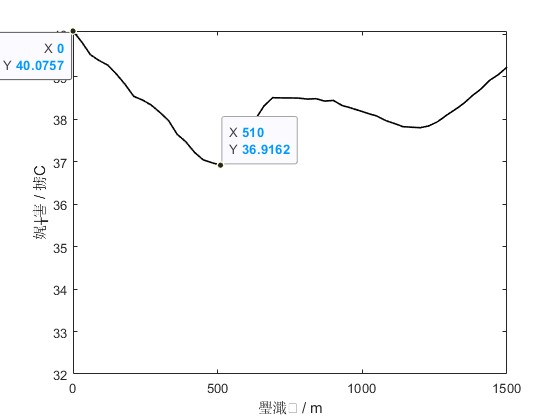

Supplement: S1 File — (ZIP) [file pone.0344026.s001.zip › Supplementary material/3 Matlab algorithms and some results/Park temperature inflection plot in Matlab/58.jpg]

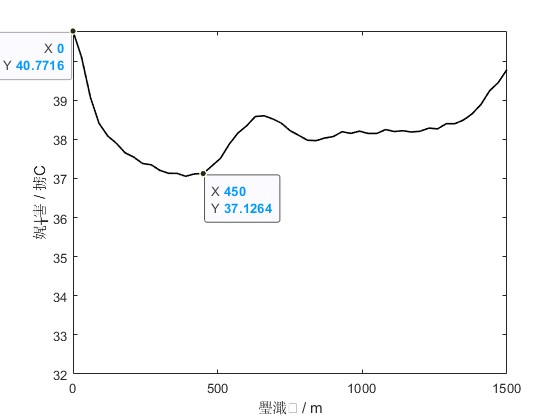

Supplement: S1 File — (ZIP) [file pone.0344026.s001.zip › Supplementary material/3 Matlab algorithms and some results/Park temperature inflection plot in Matlab/59.jpg]

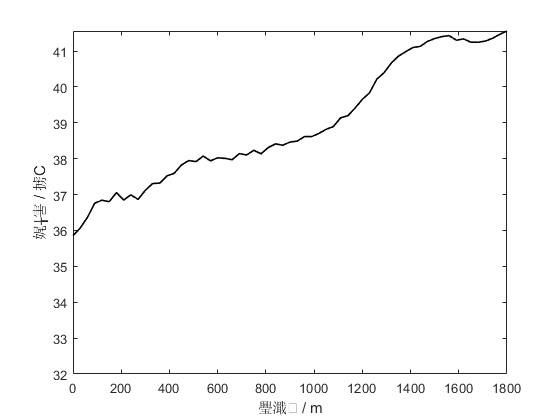

Supplement: S1 File — (ZIP) [file pone.0344026.s001.zip › Supplementary material/3 Matlab algorithms and some results/Park temperature inflection plot in Matlab/6.jpg]

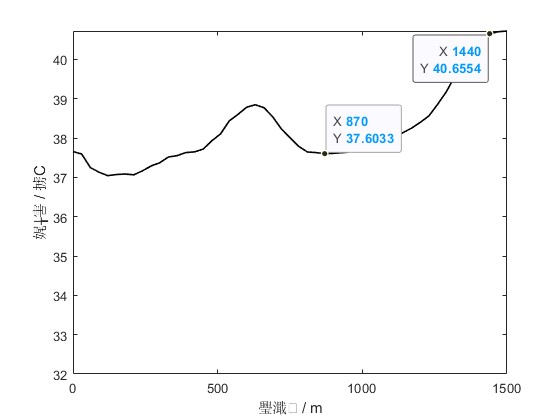

Supplement: S1 File — (ZIP) [file pone.0344026.s001.zip › Supplementary material/3 Matlab algorithms and some results/Park temperature inflection plot in Matlab/61.jpg]

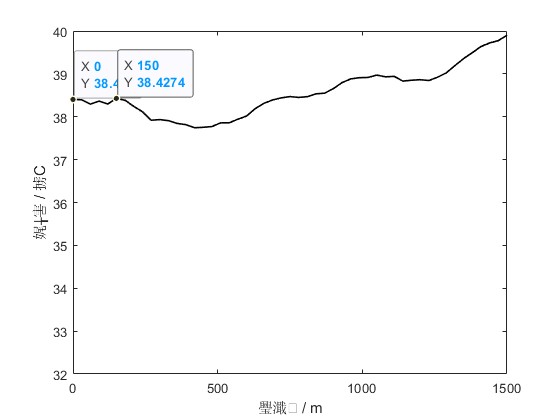

Supplement: S1 File — (ZIP) [file pone.0344026.s001.zip › Supplementary material/3 Matlab algorithms and some results/Park temperature inflection plot in Matlab/65.jpg]

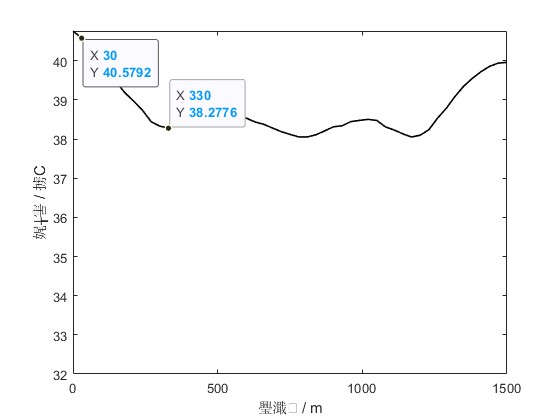

Supplement: S1 File — (ZIP) [file pone.0344026.s001.zip › Supplementary material/3 Matlab algorithms and some results/Park temperature inflection plot in Matlab/67.jpg]

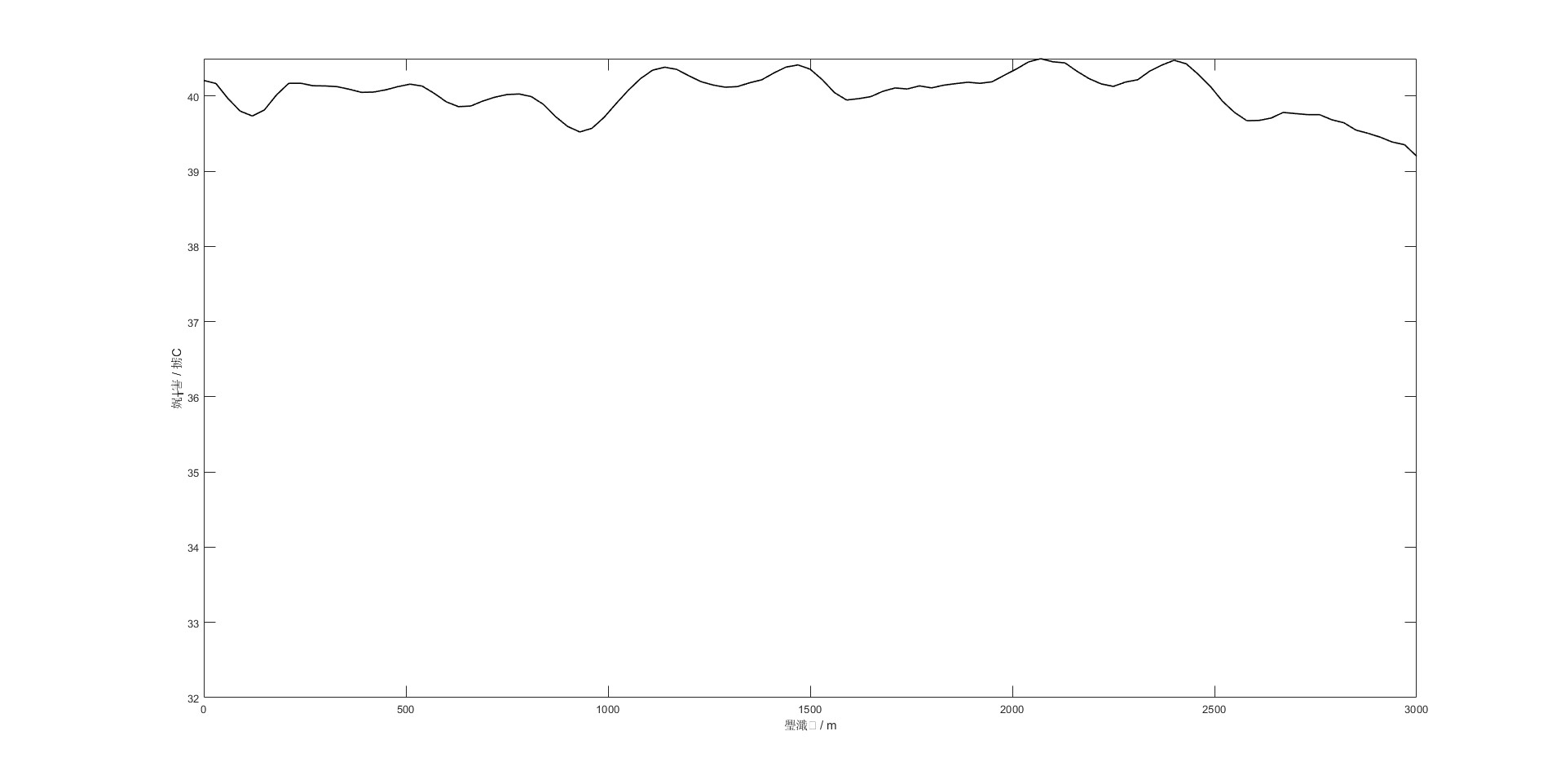

Supplement: S1 File — (ZIP) [file pone.0344026.s001.zip › Supplementary material/3 Matlab algorithms and some results/Park temperature inflection plot in Matlab/7.jpg]

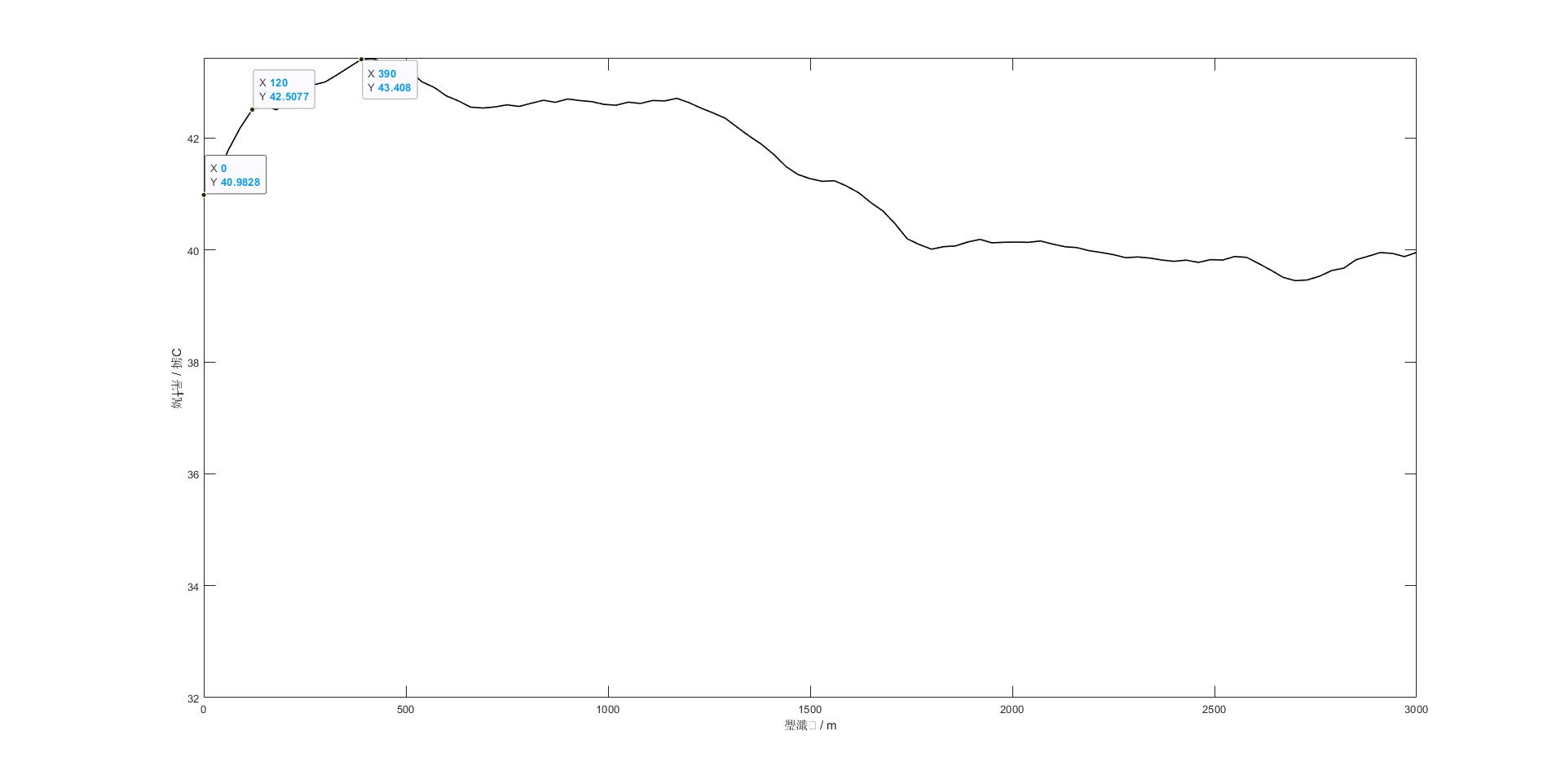

Supplement: S1 File — (ZIP) [file pone.0344026.s001.zip › Supplementary material/3 Matlab algorithms and some results/Park temperature inflection plot in Matlab/8.jpg]

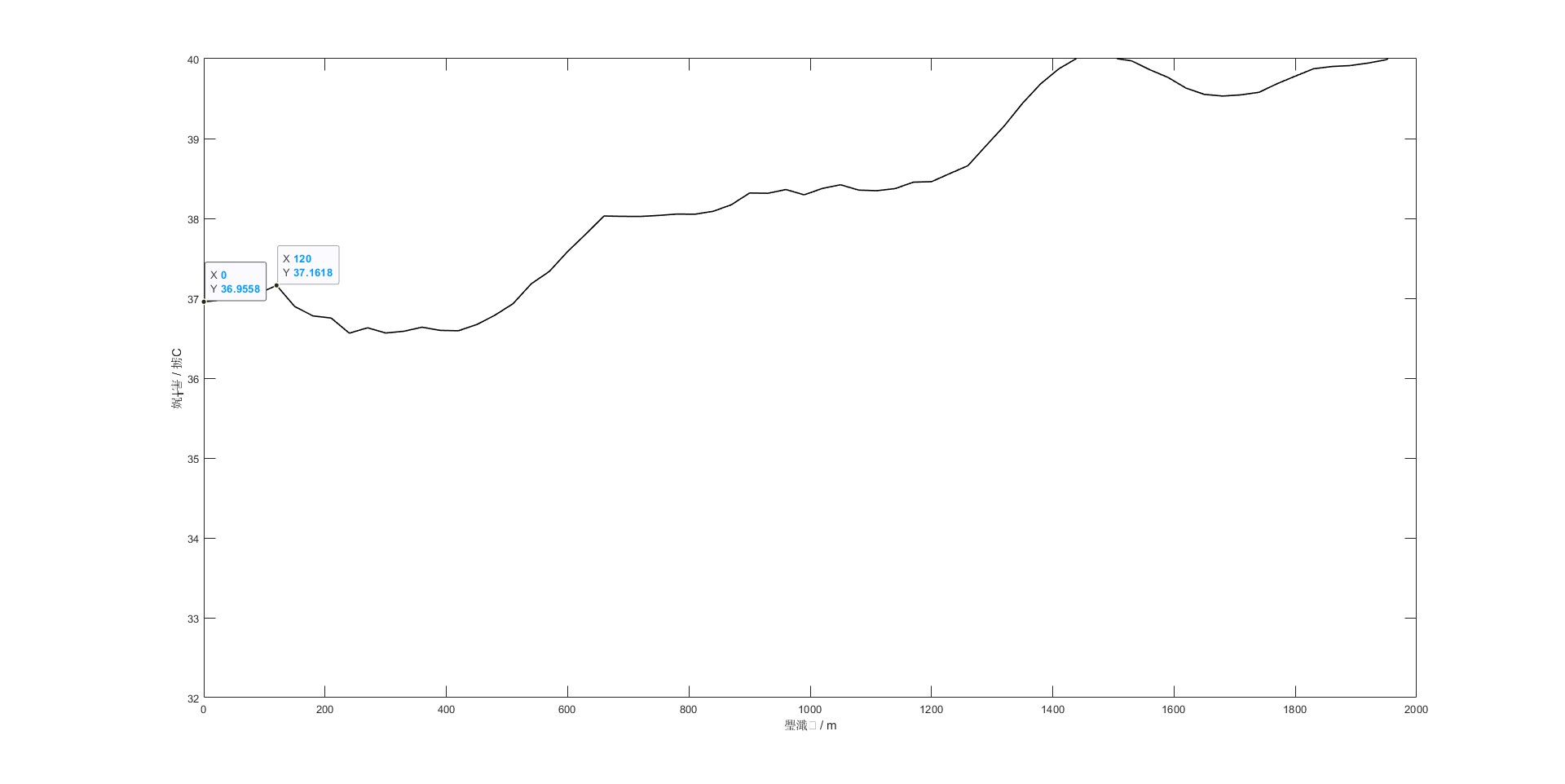

Supplement: S1 File — (ZIP) [file pone.0344026.s001.zip › Supplementary material/3 Matlab algorithms and some results/Park temperature inflection plot in Matlab/9.jpg]

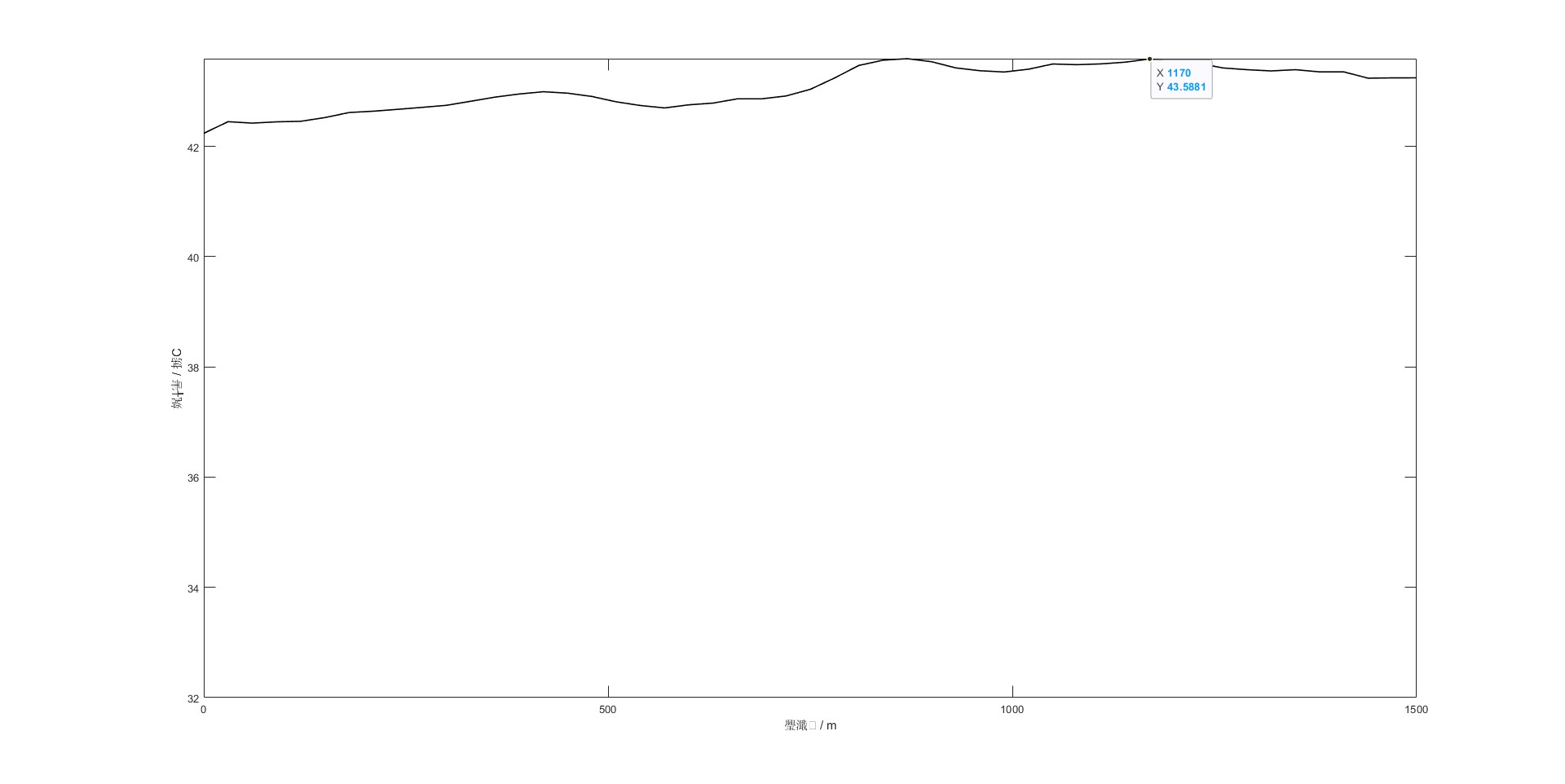

Supplement: S1 File — (ZIP) [file pone.0344026.s001.zip › Supplementary material/3 Matlab algorithms and some results/Park temperature inflection plot in Matlab/AXHGY.jpg]

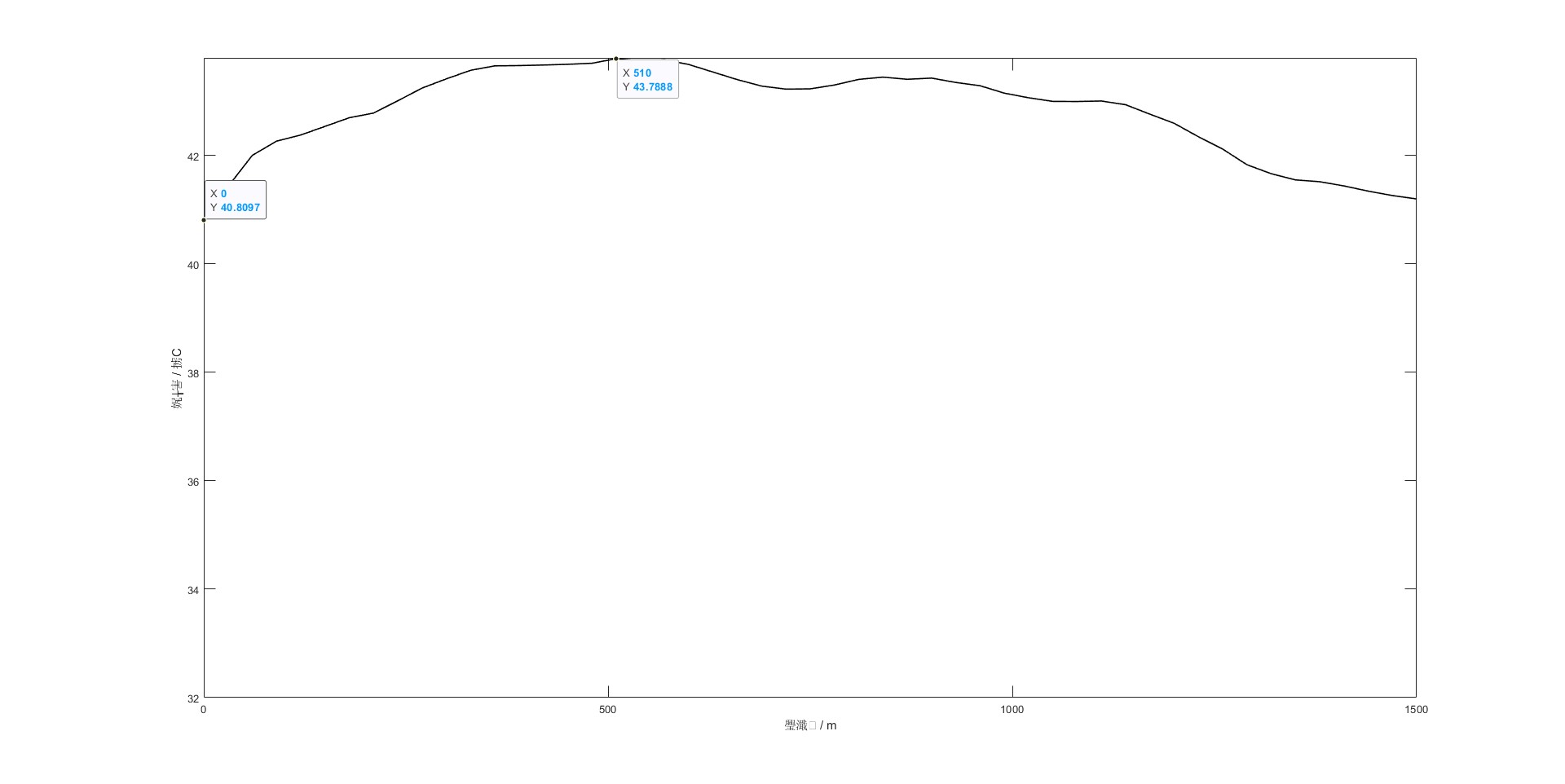

Supplement: S1 File — (ZIP) [file pone.0344026.s001.zip › Supplementary material/3 Matlab algorithms and some results/Park temperature inflection plot in Matlab/BDSRMHJQ.jpg]

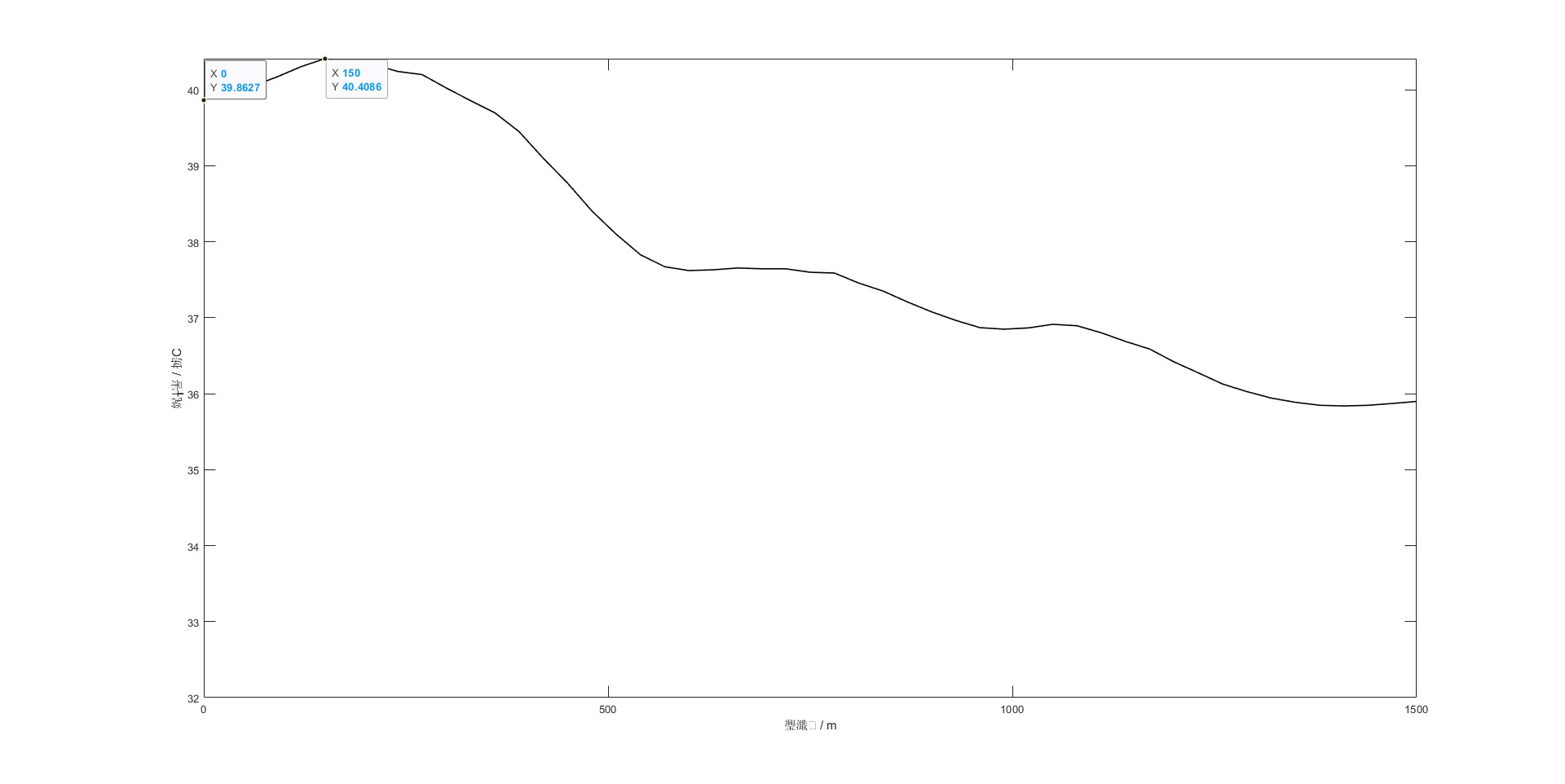

Supplement: S1 File — (ZIP) [file pone.0344026.s001.zip › Supplementary material/3 Matlab algorithms and some results/Park temperature inflection plot in Matlab/BJWHGC.jpg]

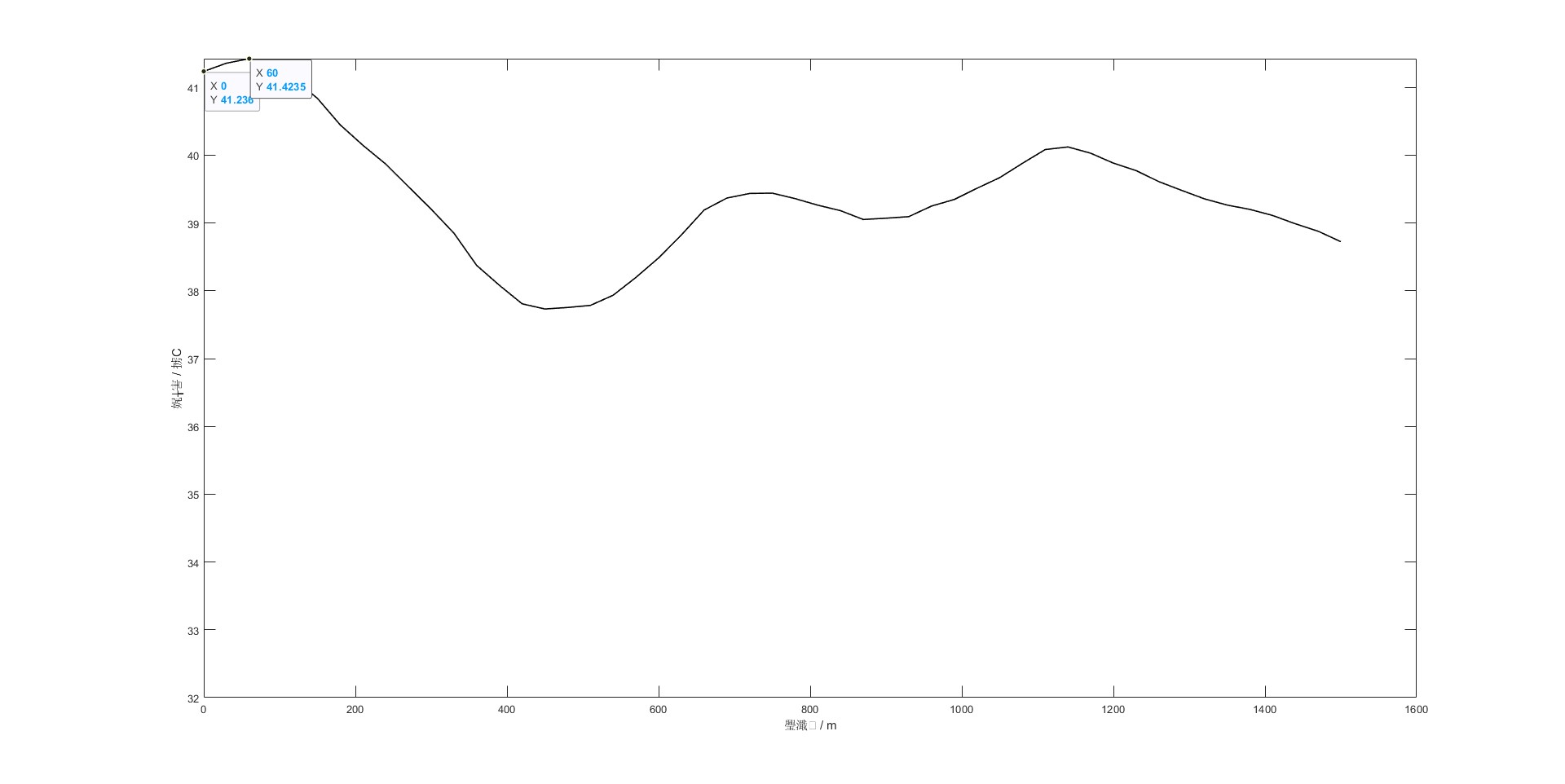

Supplement: S1 File — (ZIP) [file pone.0344026.s001.zip › Supplementary material/3 Matlab algorithms and some results/Park temperature inflection plot in Matlab/BJWHGC1.jpg]

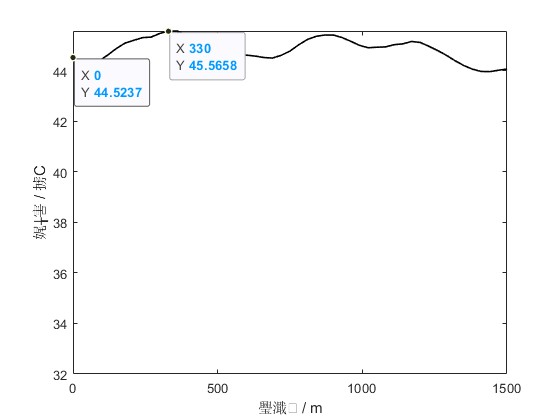

Supplement: S1 File — (ZIP) [file pone.0344026.s001.zip › Supplementary material/3 Matlab algorithms and some results/Park temperature inflection plot in Matlab/BYGY.jpg]

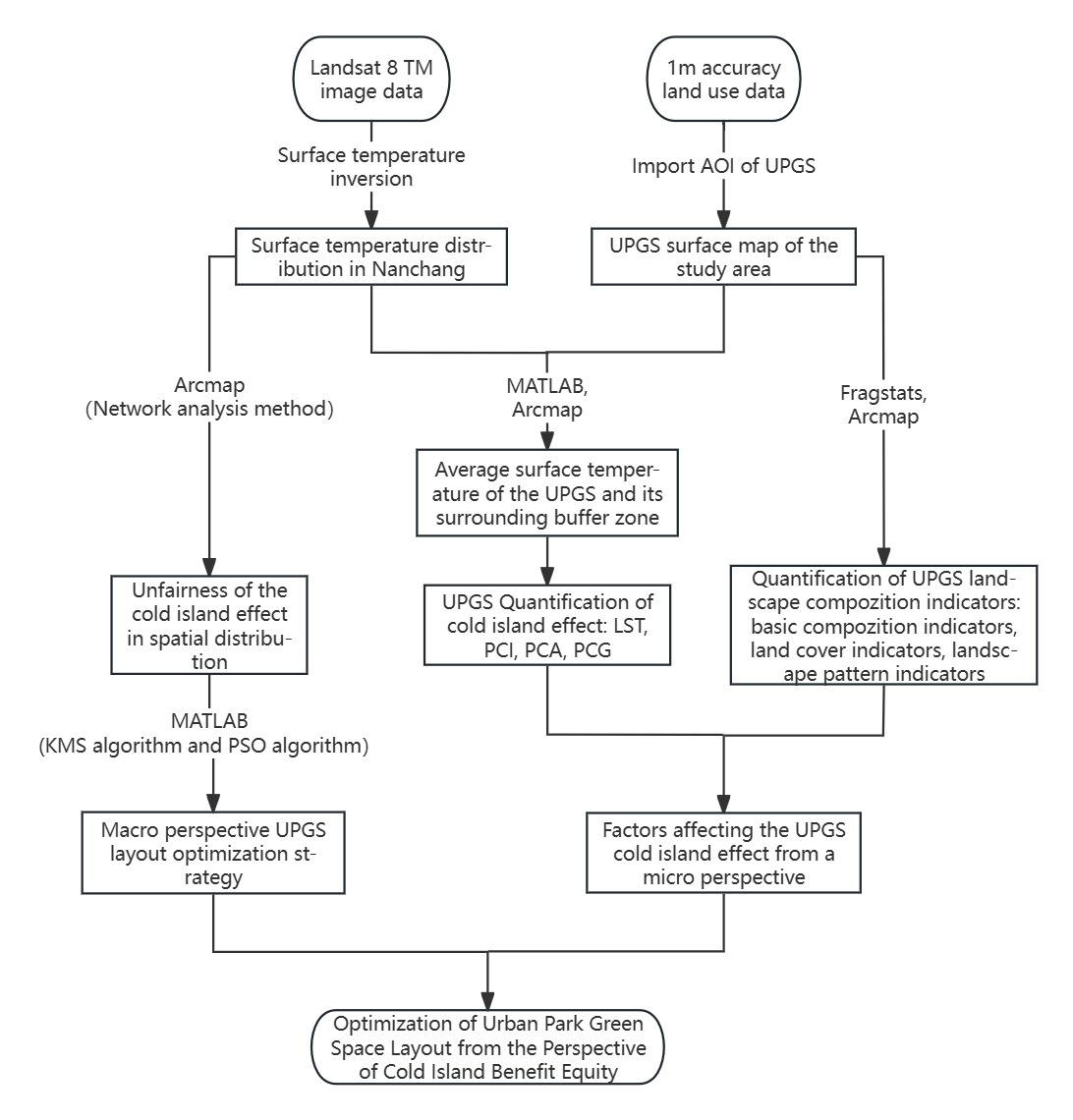

Supplement: S1 File — (ZIP) [file pone.0344026.s001.zip › Supplementary material/The thesis involves pictures/Figure 1.jpg]

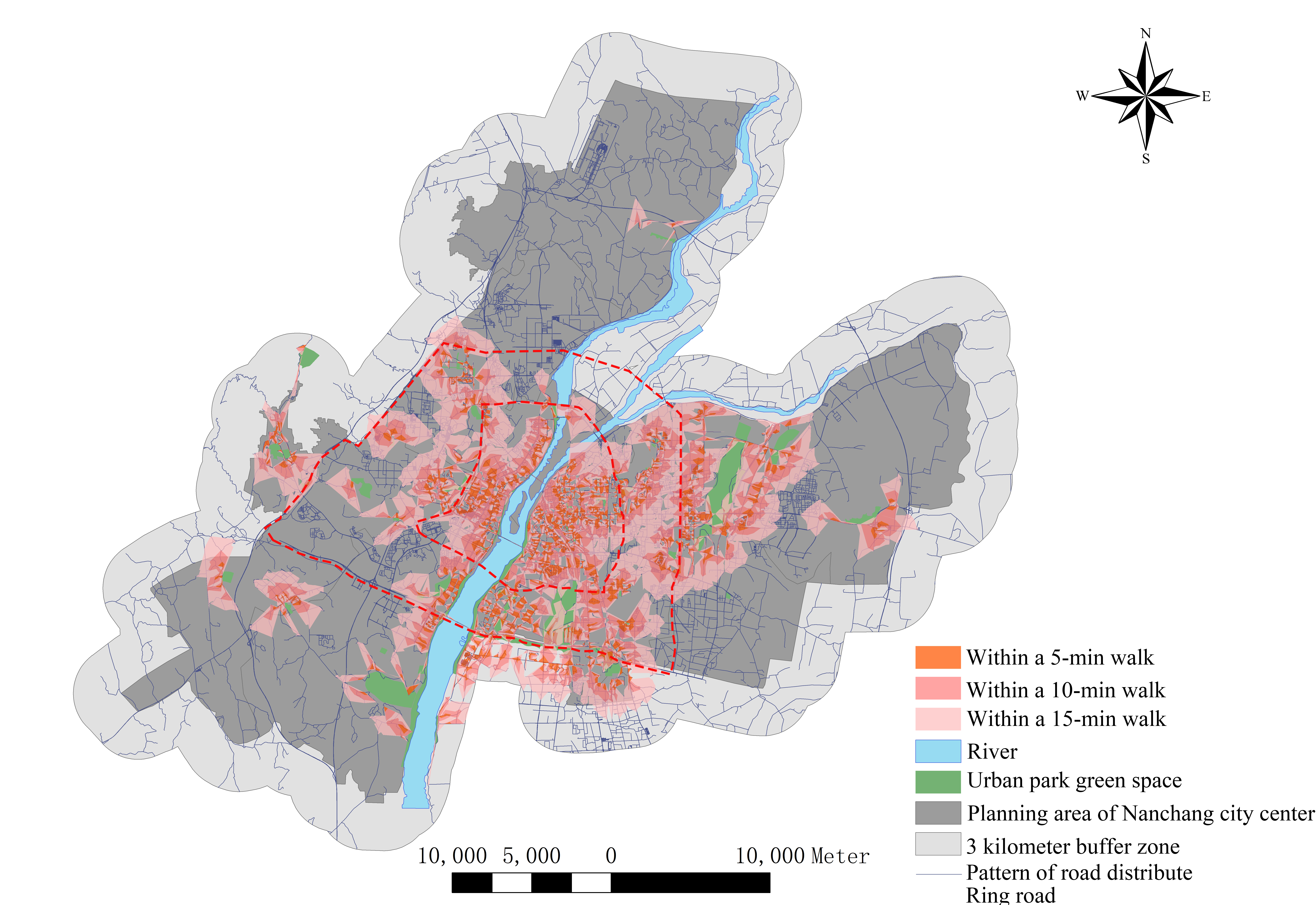

Supplement: S1 File — (ZIP) [file pone.0344026.s001.zip › Supplementary material/The thesis involves pictures/Figure 10a.jpg]

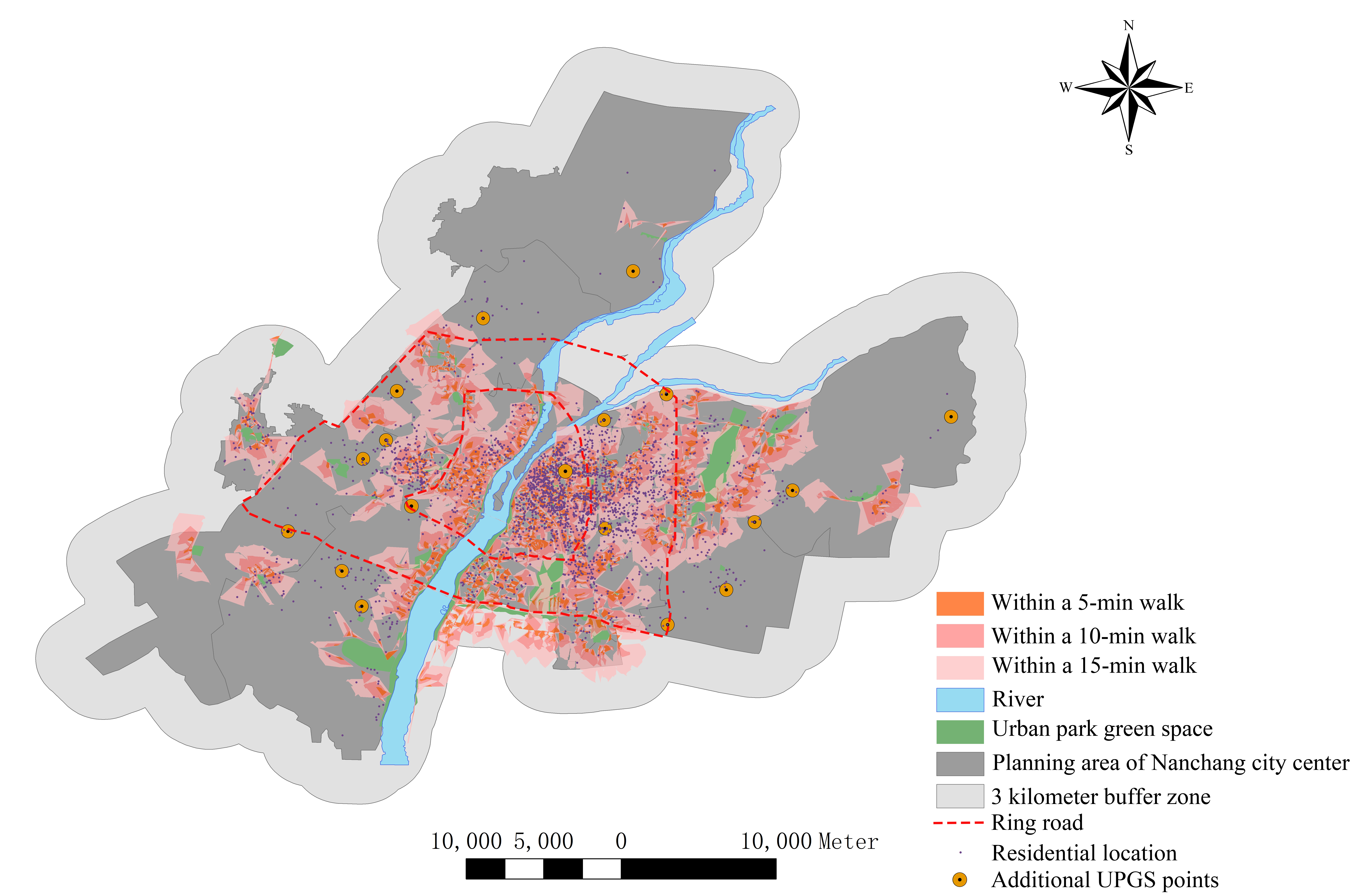

Supplement: S1 File — (ZIP) [file pone.0344026.s001.zip › Supplementary material/The thesis involves pictures/Figure 10b.jpg]

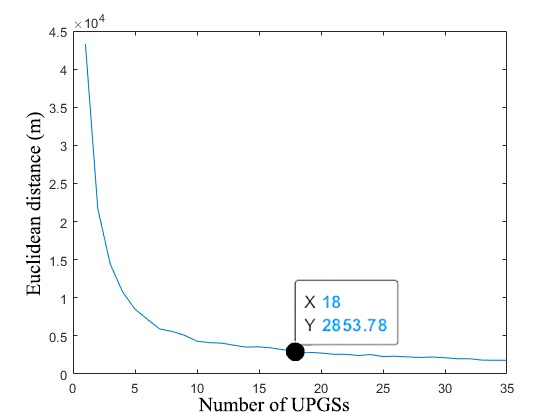

Supplement: S1 File — (ZIP) [file pone.0344026.s001.zip › Supplementary material/The thesis involves pictures/Figure 11a.jpg]

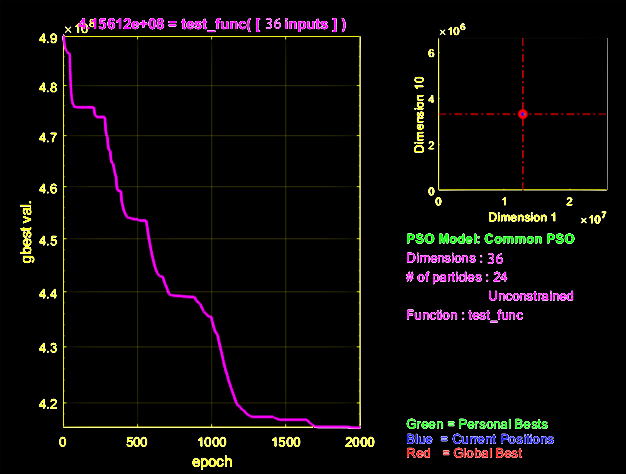

Supplement: S1 File — (ZIP) [file pone.0344026.s001.zip › Supplementary material/The thesis involves pictures/Figure 11b.jpg]

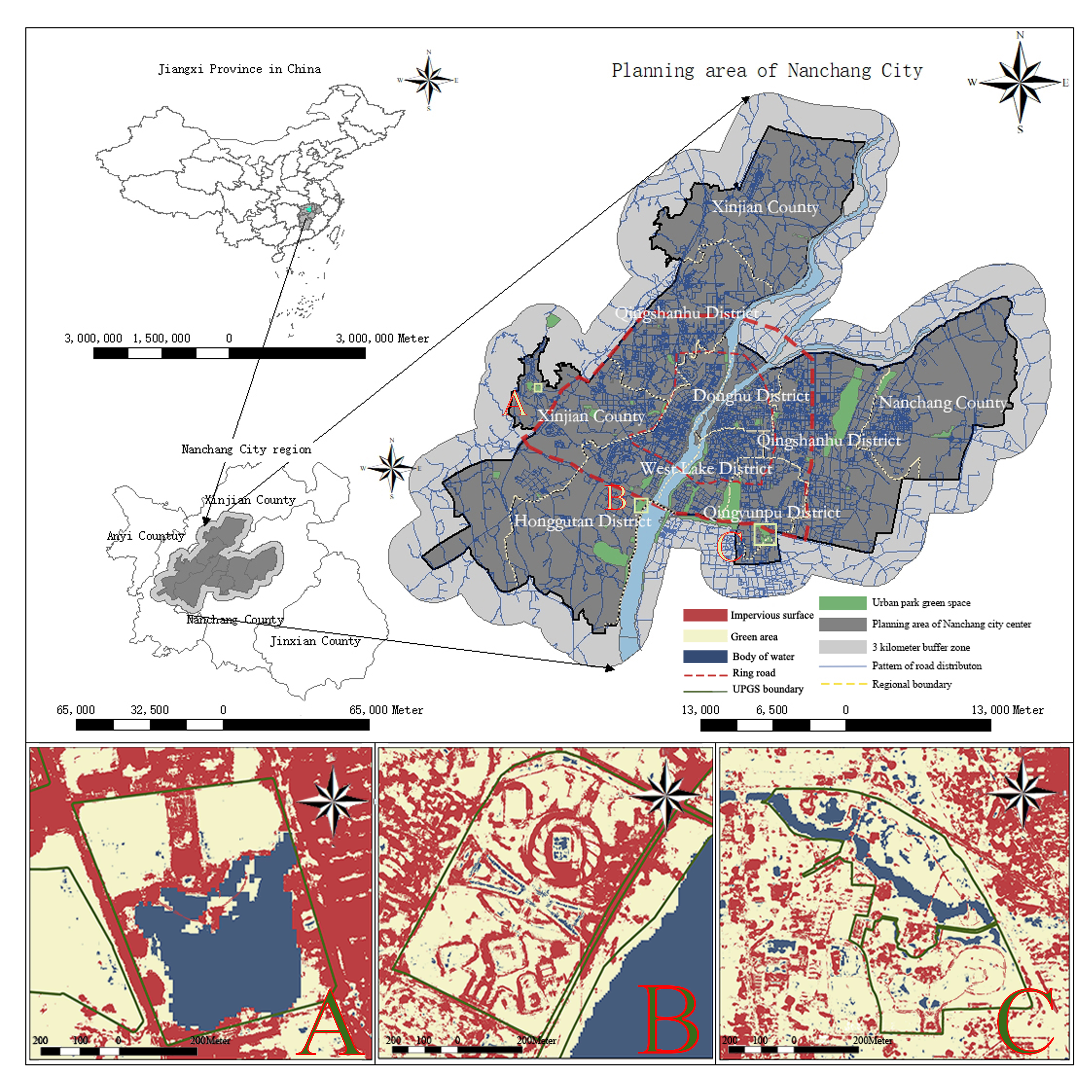

Supplement: S1 File — (ZIP) [file pone.0344026.s001.zip › Supplementary material/The thesis involves pictures/Figure 2.jpg]

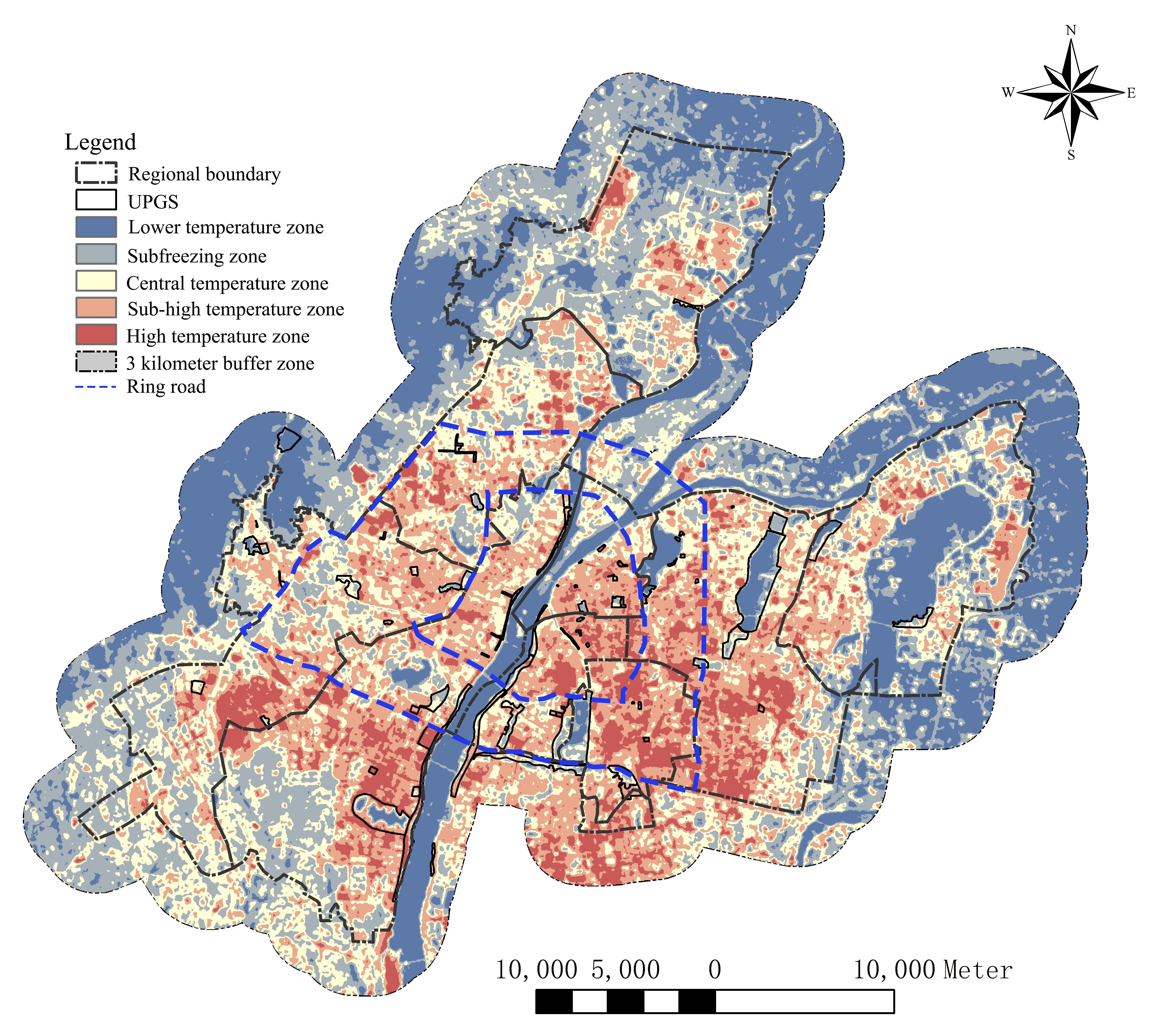

Supplement: S1 File — (ZIP) [file pone.0344026.s001.zip › Supplementary material/The thesis involves pictures/Figure 3.jpg]

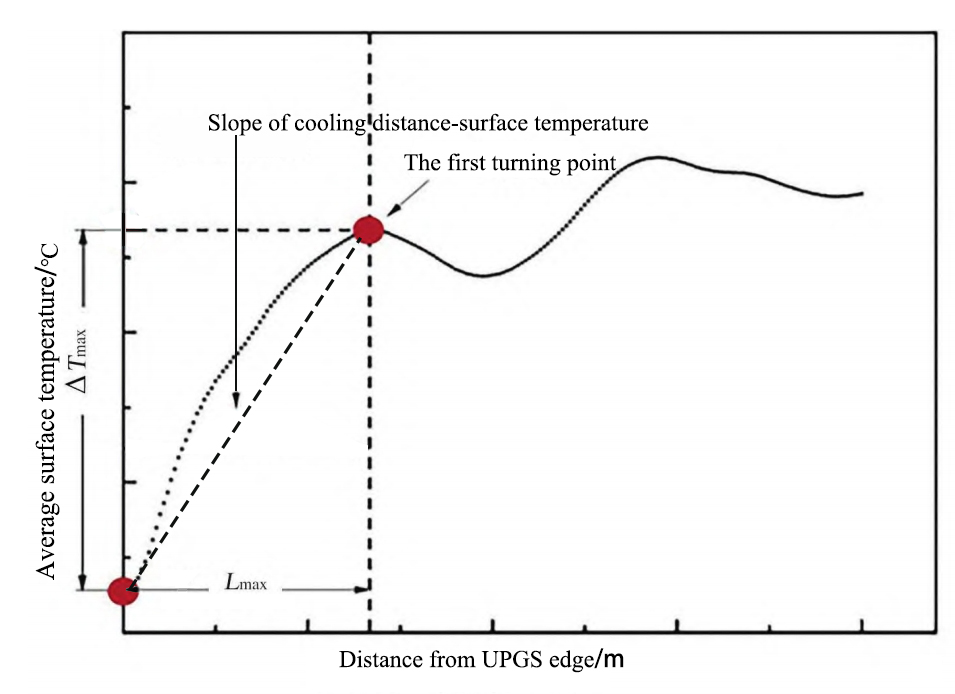

Supplement: S1 File — (ZIP) [file pone.0344026.s001.zip › Supplementary material/The thesis involves pictures/Figure 4.jpg]

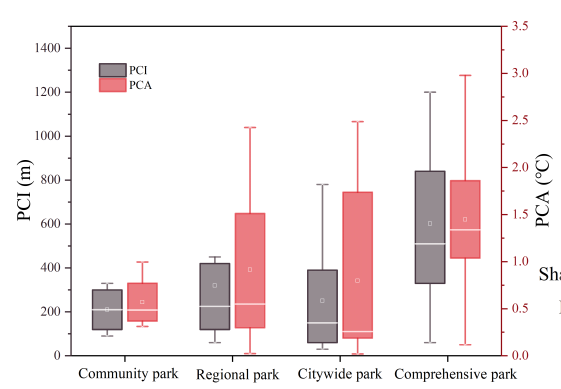

Supplement: S1 File — (ZIP) [file pone.0344026.s001.zip › Supplementary material/The thesis involves pictures/Figure 5.png]

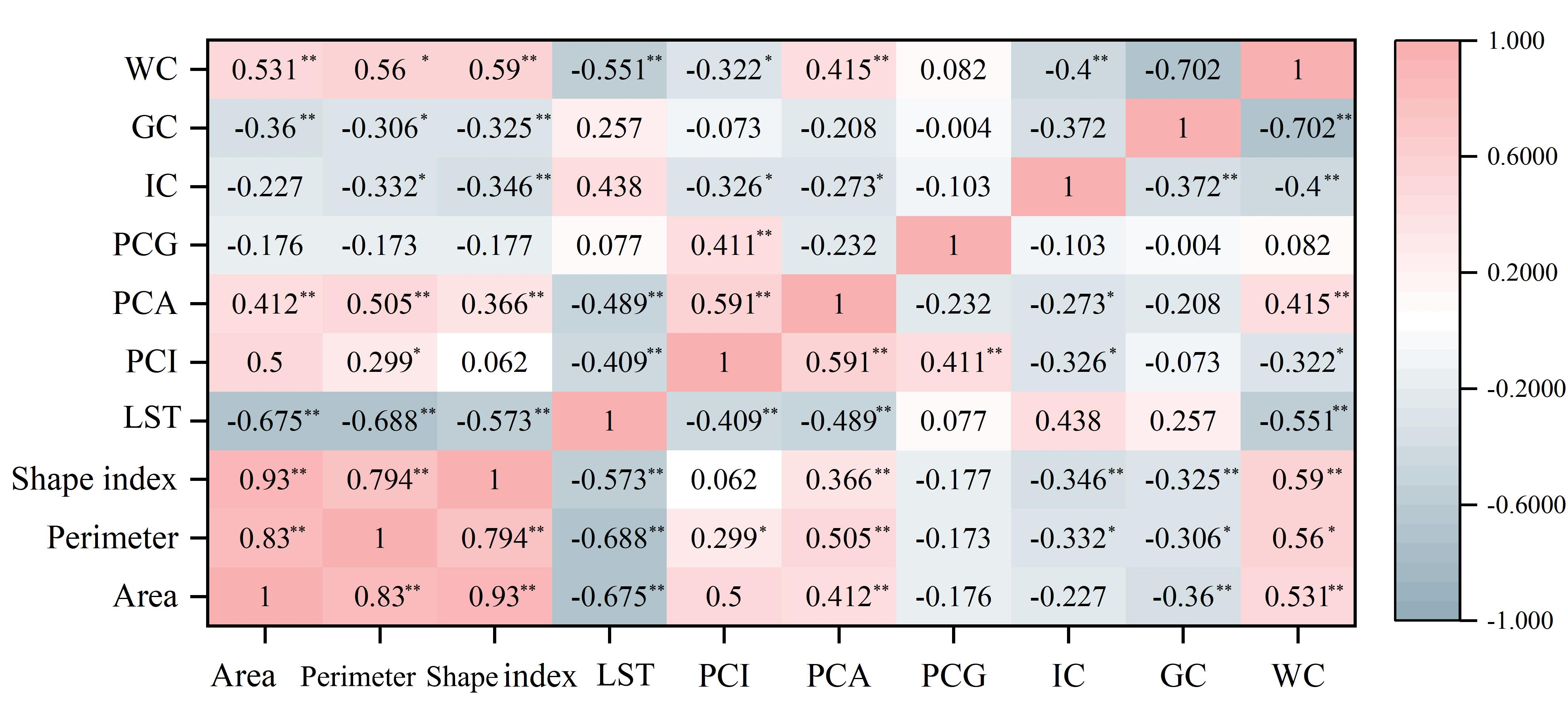

Supplement: S1 File — (ZIP) [file pone.0344026.s001.zip › Supplementary material/The thesis involves pictures/Figure 6.png]

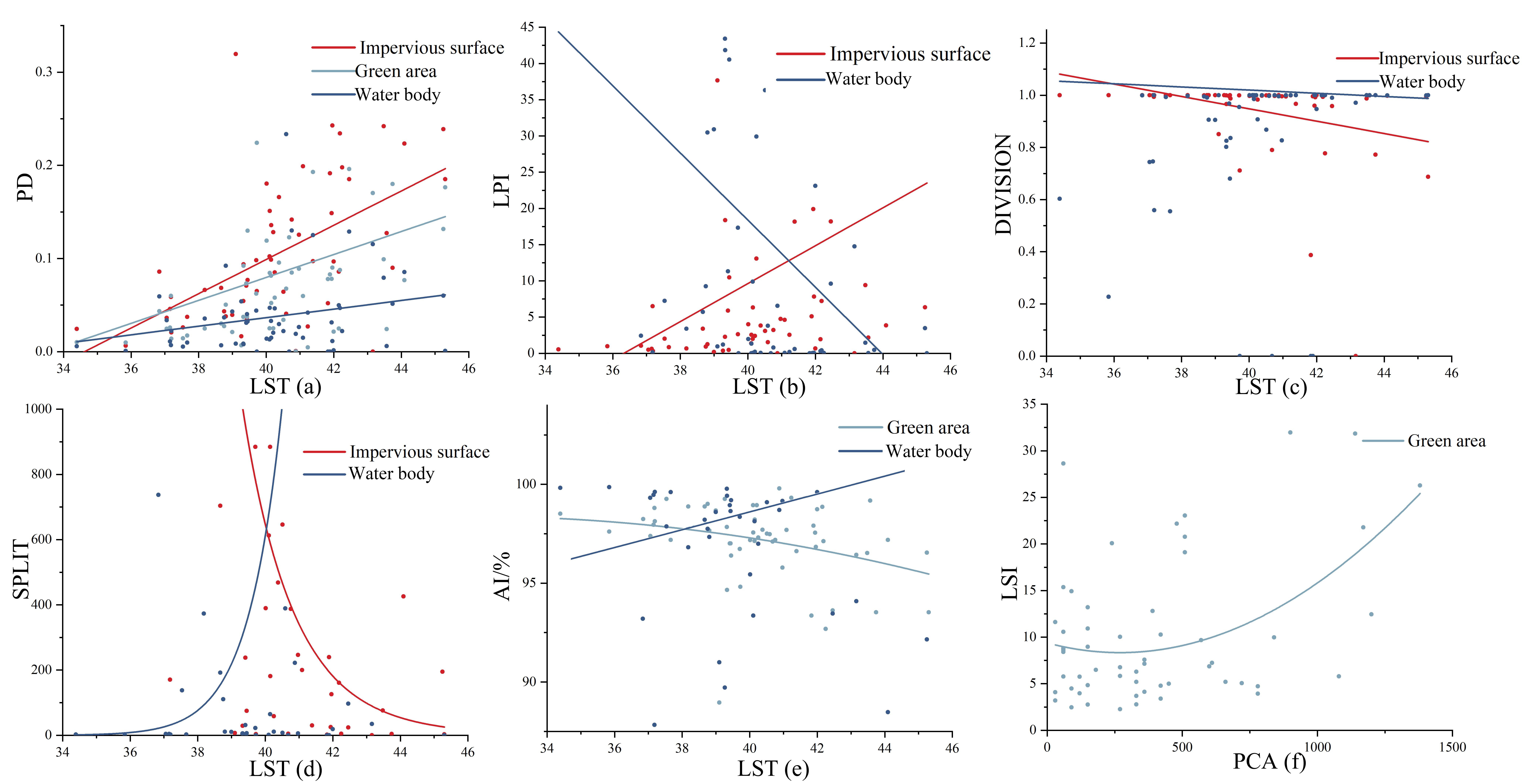

Supplement: S1 File — (ZIP) [file pone.0344026.s001.zip › Supplementary material/The thesis involves pictures/Figure 8.jpg]

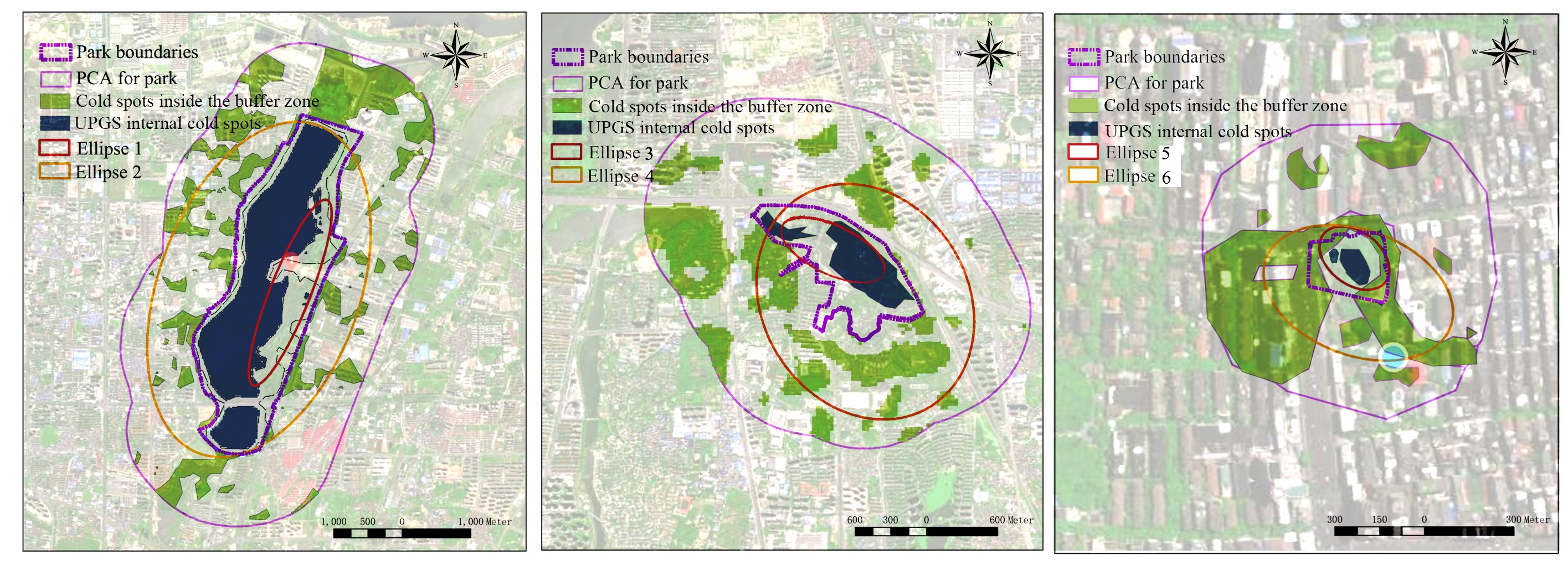

Supplement: S1 File — (ZIP) [file pone.0344026.s001.zip › Supplementary material/The thesis involves pictures/Figure 9(a-c).jpg]

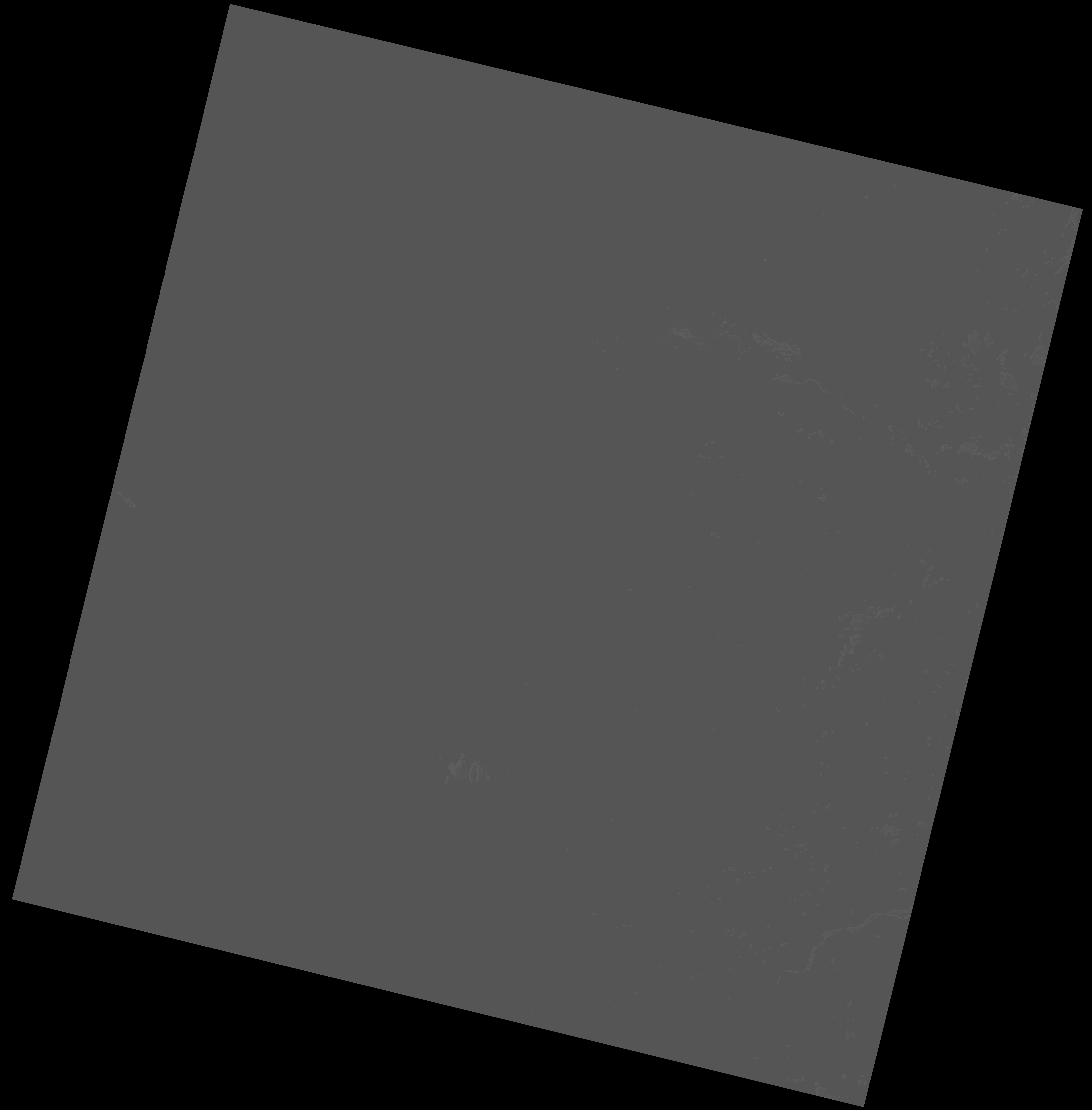

Supplement: S2 File — (ZIP) [file pone.0344026.s002.zip › Supplementary material(2)/From OpenStreetMap and Landsat/LC09_L2SP_122040_20230509_20230511_02_T1_QA_PIXEL.TIF]

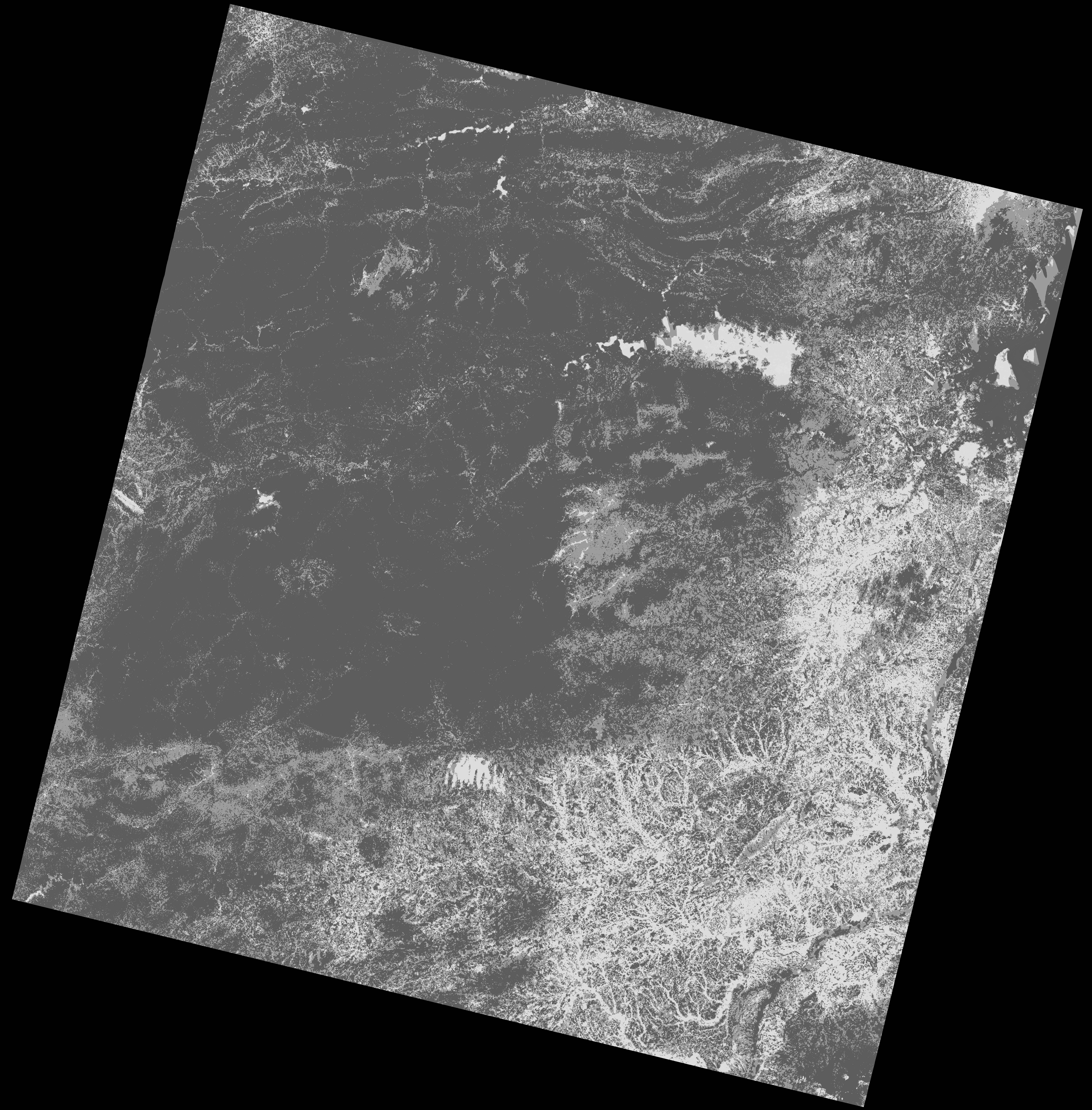

Supplement: S2 File — (ZIP) [file pone.0344026.s002.zip › Supplementary material(2)/From OpenStreetMap and Landsat/LC09_L2SP_122040_20230509_20230511_02_T1_SR_QA_AEROSOL.TIF]
