# Supplementary material for: Optimizing spatial equity of urban park cooling services: Integrating landscape metrics with K-means and PSO algorithms in Nanchang, China
Source: PLoS One. 2026 Mar 19;21(3):e0344026. doi: 10.1371/journal.pone.0344026 (PMC13001981; doi:10.1371/journal.pone.0344026)
Supplement: S1 File — (ZIP) [file pone.0344026.s001.zip › Supplementary material/3 Matlab algorithms and some results/PSO algorithm and KMS algorithm/Introduction to the use of PSO tools.doc]

**Introduction to the Use of PSO Algorithm**

1.Introduction to the PSO Toolbox

PSOt is a toolbox for PSO. It encapsulates the core parts of the PSO algorithm and provides users with adjustable parameters of the algorithm. Users only need to define the function to be optimized (for calculating the minimum or maximum value), set the value range of the function's independent variables, and the maximum allowable variation in each iteration (called maximum velocity, Max_V), etc., to perform optimization independently.

Compared with genetic algorithms, PSO can realize function optimization by adjusting only a few parameters. This algorithm has no special requirements for the function to be optimized (such as differentiability, time continuity, etc.), so it has extremely strong versatility and is more advantageous in cases involving multiple variables, high nonlinearity, discontinuity, and non-differentiability.

The use of this toolbox mainly involves the following steps:

1）Set the path of the toolbox in Matlab;2）Define the function to be optimized;3）Call the core function of the PSO algorithm: pso_Trelea_vectorized ().The third step is the most critical. Proper parameter setting according to specific needs can make the algorithm converge extremely quickly.

# The following is a detailed introduction to each step.

## Setting the Toolbox Path

 In the MATLAB Command Window, click "File -> Set Path...", as shown in the following figure:

## In the pop-up dialog box, click "Add Folder", then browse to find the location where the toolbox is placed, as shown in the following figure:

 If you want to use the test functions included in this toolbox, you need to set the path to point to the "testfunctions" folder under the toolbox using the same method as above.

# If you want to use it for neural network training, set the path to point to the "nnet" folder under the "testfunctions" folder of the toolbox.

Define the function to be optimized (see the file test_func.m)

Users can define the function to be optimized according to their own needs. For example, if you want to calculate the minimum value of the following binary function:z = 0.5*(x-3)² + 0.2*(y-5)² - 0.1where the ranges of the independent variables x and y are both [-50, 50].The function to be optimized can be defined as follows:

```
%%----------------------------------------------------------------%%
function z=test_func(in)
nn=size(in);
x=in(:,1);
y=in(:,2);
nx=nn(1);
   for i=1:nx
       temp = 0.5*(x(i)-3)^2+0.2*(y(i)-5)^2-0.1;
       z(i,:) = temp;
   end
%%----------------------------------------------------------------%%
```

It should be specially noted that the core function of the PSO algorithm, pso_Trelea_vectorized(), automatically initializes a set of random variables. Therefore, the input "in" in the function to be optimized, test_func(in), is a matrix consisting of a set of x and y values. Correspondingly, the output z of the function is a vector.Define the function to be optimized (see the file test_main.m)

After defining the function to be optimized, set the corresponding parameters, and then you can call PSO for optimization. For the above optimization problem, the call can be made as follows:

%%----------------------------------------------------------------%%

clear

clc

x_range=[-50,50];

y_range=[-50,50];

range = [x_range;y_range];

Max_V = 0.2*(range(:,2)-range(:,1));

n=2;

pso_Trelea_vectorized('test_func',n,Max_V,range)

%%----------------------------------------------------------------%%Explanation of Relevant Parameters in the PSO Algorithm

Meanings of Relevant Parameters in the Toolbox

In this toolbox, users can set 13 parameters of the PSO algorithm (see the comment section of pso_Trelea_vectorized()). However, in practice, some parameters can directly use the default values provided by the algorithm. The default values of the algorithm are explained as follows: (In the definition of the pso_Trelea_vectorized function, they are represented by the variable Pdef. If users assign values when calling pso_Trelea_vectorized(), the corresponding parameters will be overwritten. The author usually modifies the values of Pdef directly instead of specifying them during the call.)

%

1)Pdef = [**100** 2000 24 2 2 0.9 0.4 1500 1e-25 250 NaN 0 0];

P(1): The interval for display in the MATLAB command window. A value of 100 means displaying once every 100 iterations; if set to 0, no intermediate processes will be displayed.

%

2)Pdef = [100 **2000** 24 2 2 0.9 0.4 1500 1e-25 250 NaN 0 0];

P(2): The maximum number of iterations. Even if the algorithm does not converge, it will automatically stop after reaching this number.%

3)Pdef = [100 2000 **24** 2 2 0.9 0.4 1500 1e-25 250 NaN 0 0];

P(3): The number of seeds, i.e., how many seeds are initialized. For the above problem, after initialization, "in" is a matrix composed of 24 x-values and 24 y-values. A larger number of seeds increases the possibility of converging to the global optimal value but slows down the algorithm's convergence speed.

4)Pdef = [100 2000 24 **2 2** 0.9 0.4 1500 1e-25 250 NaN 0 0];

P(4), P(5): The acceleration parameters of the algorithm, which affect the local optimal value and global optimal value respectively. It is said that 2 is a good choice for most cases, so they generally do not need to be modified.

5)Pdef = [100 2000 24 2 2 **0.9 0.4** 1500 1e-25 250 NaN 0 0];

P(6), P(7): The weight values at the initial moment and convergence moment. In the earliest PSO algorithm, these parameters did not exist, and convergence was ensured by adjusting other parameters, but it was difficult to simultaneously satisfy both convergence speed and accuracy. Later, these weights were added in the improved algorithm, making it possible to balance convergence speed and accuracy. The author believes that they still do not need to be modified for most cases.

%

6)Pdef = [100 2000 24 2 2 0.9 0.4 **1500** 1e-25 250 NaN 0 0];

P(8): Specifies that when the number of iterations exceeds this value, the weight value takes its minimum (such as 0.4 mentioned above).

%

7)Pdef = [100 2000 24 2 2 0.9 0.4 1500 **1e-25** 250 NaN 0 0];

P(9): The threshold for terminating the algorithm. The algorithm stops when the optimal value of the population in two consecutive iterations is less than this threshold.

8)Pdef = [100 2000 24 2 2 0.9 0.4 1500 1e-25 **250** NaN 0 0];

P(10): The threshold for terminating the algorithm. The iteration exits if the gradient of the function remains unchanged for 250 consecutive iterations.

9)Pdef = [100 2000 24 2 2 0.9 0.4 1500 1e-25 250 **NaN** 0 0];

P(11): Used to indicate the optimization scenario. A value of NaN indicates an unconstrained optimization problem (i.e., there are no additional constraint equations).

10)Pdef = [100 2000 24 2 2 0.9 0.4 1500 1e-25 250 NaN **0** 0];

P(12): Used to specify the type of PSO algorithm to be adopted, where 0 represents the standard PSO algorithm.

%

11)Pdef = [100 2000 24 2 2 0.9 0.4 1500 1e-25 250 NaN 0 **0**];

## P(13): Used to indicate whether seeds are specified manually. A value of 0 means seeds are generated randomly, while 1 means seeds are generated by the user.

 Explanation of Important Parameters in the ToolboxAs mentioned above, among the 13 parameters listed, not all require careful specification by the user; most can simply use their default values.The parameters that truly require careful setting by the user are:The variation range of the parameters to be determined;The maximum allowable value of parameters per iteration, i.e., Max_V. It generally takes 10% to 20% of the variation range. A smaller value results in higher convergence resolution (i.e., it is less likely to skip the optimal value) but slower convergence; a larger value accelerates convergence but may cause the algorithm to jump out of the global optimal value. Therefore, users need to exercise caution.
