# Supplementary material for: Optimizing spatial equity of urban park cooling services: Integrating landscape metrics with K-means and PSO algorithms in Nanchang, China
Source: PLoS One. 2026 Mar 19;21(3):e0344026. doi: 10.1371/journal.pone.0344026 (PMC13001981; doi:10.1371/journal.pone.0344026)
Supplement: Attachment — Submitted filename: Response_to_Reviewers_auresp_2.docx [file pone.0344026.s005.docx]

### Response

### Response to the Editor

Thank you very much for your attention. First, the reviewers have not requested us to cite any specific papers. Second, we have carefully checked all the references cited in the paper, and all are in order.

### Response to Reviewer #2

We thank the reviewer for this suggestion. First, we have streamlined certain narratives in the second and final paragraphs of the Introduction, with greater emphasis on the research gaps and our contributions. Second, we have added explanations for the selection of statistical tests in Section 2.2.4, while the limitations of the data have already been elaborated in detail in Section 4.4. Furthermore, discussions on relevant policies involve certain practical complexities in the Chinese context, and we would appreciate your understanding in this regard.Finally, we have rechecked the consistency of language and formatting per your comments and made partial revisions to the manuscript.
